# Contact-Time and Relaxation Effects on the Normal Response of Polymer-Coated Internal Cylindrical Surfaces with Deterministic Microtexture: A Layered Viscoelastic Contact Model

**DOI:** 10.3390/polym18212638

**Published:** 2026-10-24

**Authors:** Kirill A. Bashmur, Alexander V. Zagulyaev, Ivan S. Nekrasov, Elena V. Tsygankova, Tatiana N. Sviridova

**Affiliations:** 1 Department of Technological Machines and Equipment of Oil and Gas Complex, Siberian Federal University, 660041 Krasnoyarsk, Russia; 2 Department of Foreign Languages for Natural Sciences, Siberian Federal University, 660041 Krasnoyarsk, Russia

**Keywords:** polymer coating, viscoelasticity, bonded coating, oedometric constraint, deterministic microtexture, internal cylindrical surface, contact stiffness, pressure–approach response

## Abstract

Deterministic microtexture and viscoelastic coating deformation affect the normal response of polymer-coated internal cylindrical surfaces. An additive model separates statistical land roughness, deterministic Fourier displacement, and the coated-minus-bare periodic-cell increment. Half-space (HS) and finite-thickness (FT) single-asperity laws describe the same prescribed summit population for isolated imprints (class A) and a connected helical groove (class B). At 1 MPa, the relaxed texture factor exceeds the instantaneous factor for both classes, and class A exceeds class B. Across the sampled open-contact states, secant and tangent stiffness increase sublinearly with pressure; tangent stiffness exceeds secant stiffness, and relaxation reduces both. Substrate confinement lowers the class A instantaneous total normal approach from 1.626 μm in HS to 1.139 μm in FT and its instantaneous-to-relaxed increase from 3.23% to 1.27%. For both texture classes in the FT branch, the total normal approach remains below the residual texture depth at all evaluated states and nominal pressures in the range 0.1–5 MPa. Prescribed azimuthal pressure patterns retain their stiffness-spread ranking across classes and elastic endpoints. For a relaxation time of 250 μs, a fixed-location hold of 0.509 ms develops approximately 84% of the endpoint change in the hereditary FT base approach and increases the total approach by 1.07% relative to its instantaneous value. Local hereditary histories also resolve surface recovery during unloading. With component-level verification, the framework supports comparisons of texture, confinement and loading duration while identifying intercomponent coupling as a question for a fully coupled reference solution.

## 1. Introduction

Polymer coatings are used in sliding contacts, hydraulic components, bearings, seals, and tubes to provide corrosion protection and modify compliance, damping, and tribological behavior. Their normal response is time dependent: loading duration, temperature, and the relaxation spectrum affect normal approach under load and recovery after unloading [1,2,3,4,5]. In internal cylindrical contacts, this time dependence influences local load sharing and the resulting normal stiffness of the coated interface, particularly when deterministic microtexture reduces the load-bearing fraction. Here, deterministic microtexture denotes a prescribed, repeatable surface pattern introduced by design, while statistical land roughness is represented separately. ISO 25178-2 provides the areal-topography terminology, whereas ISO 21920-2 is used for profile terms [6,7]. Recent studies have examined related texture and contact effects in micro-pitted, burnished, grooved, and machined surfaces [8,9,10,11].

Burnishing and related processes based on surface plastic deformation can form such topographies without material removal [12,13,14,15]. In tube-bore burnishing, positive relative curvature and restricted access make the resulting texture geometry dependent on tool compliance. The compliant-crown tool configuration described in [16] provides a means of forming the metallic microtexture. The contact analysis uses a prescribed geometry of a metallic surface textured before coating and subsequently subjected to nominal pressure in service.

Isolated imprints and a helical groove represent disconnected and connected texture topologies, respectively. Experiments on polymer-coated textured substrates show that discrete texture arrays can retain wear debris and support lubricant-film formation, whereas connected groove networks can provide coating interlocking and solid-lubricant replenishment pathways [17,18]. These contrasting functions motivate a quantitative comparison of their coated normal-contact response.

Elastic models link deterministic texture geometry to rough-contact stiffness for regular dimples and broader texture classes [19,20]. Coated-contact formulations account for layer effects through microcontact corrections, indentation-dependent effective properties, finite-thickness Green functions and single-asperity solutions that resolve the transition from coating- to substrate-controlled deformation [21,22,23,24]. Spectral theory shows how layered rough-contact response changes when relevant roughness wavelengths become comparable with coating thickness [25]. Two-scale formulations transfer roughness-induced compliance into nominal contact problems for elastic and viscoelastic bodies [26]. Analyses of nearly incompressible coatings and thin viscoelastic layers describe through-thickness response under lateral restraint using the oedometric modulus [27,28]. These approaches motivate the distinction between statistical land-roughness deformation and the cell-averaged displacement increment produced by adding a bonded coating.

The model quantifies how texture topology, coating–substrate confinement, and loading duration affect normal approach and stiffness. Periodic cells solved by the finite-element method (FEM) provide the coated-minus-bare displacement increment, while half-space (HS) and finite-thickness (FT) single-asperity laws describe the same prescribed summit population. Elastic endpoints, local hereditary loading and unloading histories, and prescribed pressure patterns provide complementary comparisons. Numerical verification and published component-level experiments establish the evidence for individual parts of this framework. Its use for a specific coated cylindrical contact requires corresponding material, topographic, and loading data.

## 2. Materials and Methods

The physical contact pair consists of one deformable coating/substrate stack against a rigid, smooth, frictionless counterface; the rough-contact and through-thickness compliances therefore refer to the same coated surface.

The contact formulation describes a local planar patch of an internal cylindrical surface. Contact and texture lengths are assumed small relative to the local bore radius, $L_{loc}/R_{i}\ll 1$. The computational depth $h_{s}=2.70\;\mathrm{mm}$ defines the local fixed-bottom substrate; macroscopic cylinder deformation requires a separate structural model.

### 2.1. Scope, Prescribed Texture Input, and Parameters

The reference tube-bore process first forms the deterministic microtexture on the bare metallic surface. The polymer coating is then applied, and the coated interface is evaluated under a prescribed nominal pressure $q_{nom}$. The forming force and subsequent contact pressure describe these separate loading stages.

The prescribed numerical range $0.1\leq q_{nom}\leq 5$ MPa spans progressive summit engagement and branch-dependent proximity to residual-depth closure. Within this interval, the HS closure criterion is reached by class A at both elastic endpoints and by relaxed class B; the instantaneous class B HS closure pressure is 5.275 MPa. For both texture classes in the FT branch, the total normal approach remains below the residual texture depth at every evaluated state and pressure in this interval. Values beyond a branch-specific closure limit are explicitly marked as formal continuations of the open-texture law. The forming force defines the texture geometry, whereas $q_{nom}$ specifies the subsequent contact loading. The pressure range is an input to this parametric comparison.

Both texture classes use the same material law and subsequent contact formulation. Class A consists of isolated imprints whose topological union defines the periodic aperture. The zero mode determines the open fraction, and the nonzero modes give the deterministic Fourier displacement increment $\delta_{rmr}$. Class B is a connected helical groove represented by its deepest transverse plane section and checked against a three-dimensional stripe cell, with $\delta_{rmr}=0$ in the adopted decomposition. Both classes use the same prescribed summit population: the HS branch uses Hertz single-asperity contact, and the FT branch uses the layered coating/substrate response.

### 2.2. Prescribed Texture Geometry and Land-Pressure Amplification

**Figure 1:**
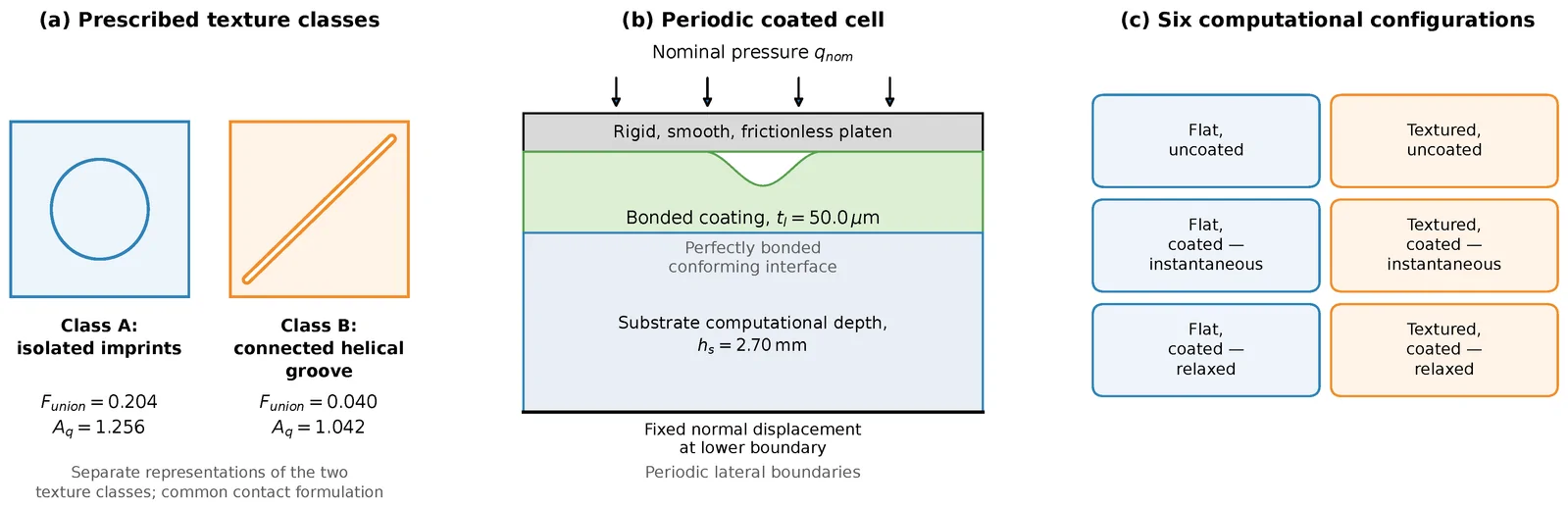
Computational setup. (**a**) Independent representations of class A as isolated imprints and class B as a helical groove. (**b**) Periodic coated cell over the elastic substrate, loaded through a rigid, smooth, frictionless platen at nominal pressure $q_{nom}$. (**c**) Six FEM configurations evaluated at each pressure on the reference and fine FEM discretizations. The substrate depth is $h_{s}=2.70\;\mathrm{mm}$; only normal displacement is fixed at the lower boundary of the periodic cell.

The prescribed texture classes, the periodic coated cell, and the six FEM configurations are summarized in Figure 1. The residual texture depth $h_{res}=2.702\;\mu m$ (2.70μm to three significant figures) and the class-specific topology are prescribed for the reference parametric case. For class A, the open fraction $F_{union}$ is derived from the periodic ellipse mask and cell area, and $A_{q}={\left(1-F_{union}\right)}^{-1}$ is then obtained algebraically. The prescribed geometry parameters and their sources are listed in Supplementary Section S1.5.

For class A, the open area is the union of the residual imprint footprints within a periodic cell. Overlapping regions and features crossing a periodic boundary are counted once. The open fraction and land-pressure amplification factor are

(1) $$F_{union}=\frac{A_{union}}{A_{cell}},\;A_{q}=\frac{1}{1-F_{union}},\;q_{land}=A_{q}q_{nom}.$$

For class A, the same open texture defines the binary aperture used by the deterministic periodic term. For class B, the texture is represented by a connected helical groove rather than by isolated imprints, and the class-specific plane-section formulation is used (Figure 1).

### 2.3. Nominal Material Parameters and Substrate

The coating is represented by an isotropic standard linear solid (SLS) with constant bulk modulus *K* and a single shear-relaxation time,

(2) $$G_{R}\left(t\right)=G_{\infty }+\left(G_{0}-G_{\infty }\right)e^{-t/\tau },\;K=5000\;\mathrm{MPa},\;G_{0}=1380\;\mathrm{MPa},\;G_{\infty }=1210\;\mathrm{MPa}.$$

The relaxation time is τ=250μs=0.250 ms [5]. Together with the stated moduli, it defines a literature-based reference material for the contact calculations. Application to a particular coating requires measured constitutive parameters for that material.

The instantaneous and relaxed elastic constants follow from

(3) $$E\left(G\right)=\frac{9KG}{3K+G},\;\nu \left(G\right)=\frac{3K-2G}{2(3K+G)},\;E^{*}\left(G\right)=\frac{E(G)}{1-\nu {\left(G\right)}^{2}}.$$

Setting $G=G_{0}$ or $G_{\infty }$ gives the corresponding elastic endpoint. Time-dependent use of these algebraic conversions is confined to the quasi-elastic (QE) approximation; the hereditary formulation in Section 2.6 uses operational moduli. The coating thickness is $t_{l}=50\;\mu$m. The perfectly bonded elastic substrate has $E_{s}=72$ GPa, $\nu_{s}=0.33$, and computational depth $h_{s}=2.70$ mm, which defines the local numerical stack.

Other substrate properties and computational depths can be assessed by recomputing the FT response. Perfect bonding in the periodic finite-element cells is enforced by a conforming coating/substrate mesh with shared interface nodes. Table 1 summarizes the prescribed material parameters and derived scales.

### 2.4. Bonded-Coating Model and Texture Factor

Perfect bonding restrains lateral expansion and increases the resistance to through-thickness deformation. At elastic endpoint j∈{0,∞}, $E_{j}/\left(1-\nu_{j}^{2}\right)$ is the one-sided contact modulus, whereas $M_{j}=K+4G_{j}/3$ is the modulus for fully constrained normal strain. The latter provides the local oedometric reference for coating compression. The coated-minus-bare increment also includes the change in substrate displacement induced by the coating. The Navier/Fourier benchmark in Section S1.3, the local asperity kernel, and the macroscopic boundary-condition (BC) matrix concern the distinct elastic configurations specified below.

The local oedometric reference is $M_{oed}\left(G\right)=K+4G/3$; at endpoint *j*, $M_{oed}\left(G_{j}\right)=M_{j}$. The coated-minus-bare increment is written as

(4) $$\delta_{l,j}=\psi_{j}\frac{t_{l}q_{land}}{M_{j}},\;M_{j}=K+\frac{4}{3}G_{j},\;j\in \left\{0,\infty \right\}.$$

The increment $\delta_{l}$ is the difference between coated and bare cell responses under the same nominal load and boundary conditions. It includes the substrate-displacement change caused by adding the coating. Local coating compression measures deformation across the coating thickness, whereas $\delta_{l}$ measures the change in the cell response. The texture factor ψ scales the coated-minus-bare increment relative to the oedometric reference and enters only that contribution.

**Figure 2:**
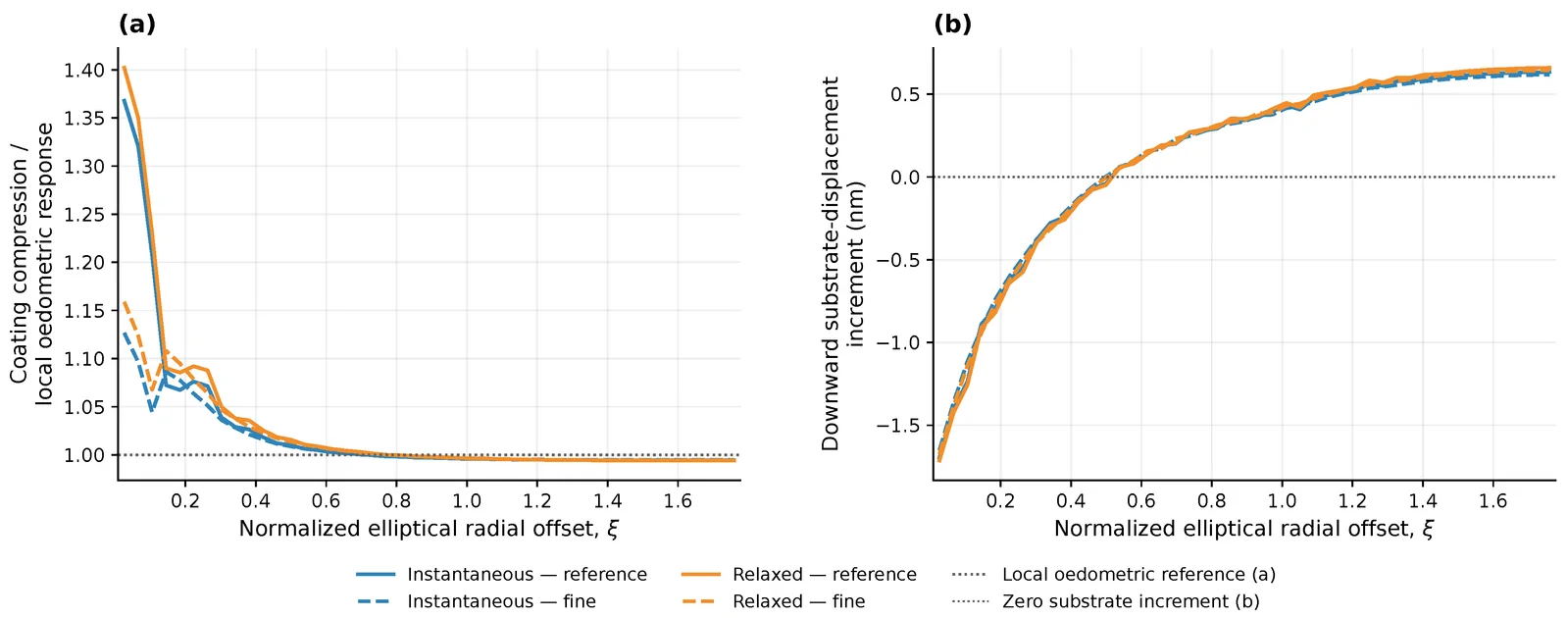
Area-weighted local deformation profiles for class A at $q_{nom}=1\;\mathrm{MPa}$. (**a**) Local coating compression, divided pointwise by $t_{l}p_{loc}/M_{j}$ for the corresponding instantaneous (j=0) or relaxed (j=∞) state and then area averaged within each bin. (**b**) Coated-minus-bare increment in downward substrate displacement under matched nominal loading. The 45 common bins are defined by the elliptical radial offset ξ. Solid and dashed curves denote the reference and fine finite-element discretizations, respectively. Dotted lines indicate the local oedometric reference in (**a**) and zero increment in (**b**). The near-edge extrema remain mesh sensitive.

The reference periodic cell has periodic displacement fluctuations, zero mean lateral normal strains, and a fixed normal displacement at the bottom. A tangential anchor removes rigid translation. In Figure 2a, local coating compression is the difference between the normal displacements of the coating’s top and bottom surfaces. This difference is first divided, at each sampling point, by the local oedometric estimate $t_{l}p_{loc}/M_{j}$ for the corresponding elastic endpoint; the pointwise ratios are then averaged within each bin using area weights. The resulting ordinate describes compression relative to the local reference, whereas absolute compression and the coated-minus-bare increment are separate observables.

The dimensionless coordinate $\xi =\sqrt{{\left[\left(x-x_{c}\right)/a_{ax}\right]}^{2}+{\left[\left(y-y_{c}\right)/a_{circ}\right]}^{2}}-1$, with $\left(x_{c},y_{c}\right)$ at the imprint center and $a_{ax}$ and $a_{circ}$ the axial and circumferential semi-axes, respectively, measures elliptical radial offset from the boundary. The 45 common bins are defined by ξ and combine sampling points at different angular positions, so ξ differs from the shortest distance to the edge. Comparing the binned compression and pressure helps interpret the normalized profile: in the fine instantaneous results, an intermediate rise of about 4.0% in the ordinate accompanies a 1.9% decrease in mean compression and a 5.6% decrease in mean pressure (Section S11.1). Thus, the normalized response can rise while absolute compression falls. Edge restraint and pressure redistribution offer plausible physical contributions to the full decrease–increase–decrease profile, but their individual roles in that sequence remain unresolved. The positions and amplitudes of the extrema are also mesh sensitive. Figure 2b presents the corresponding coated-minus-bare increment in substrate displacement.

### 2.5. Normal-Approach Decomposition and Stiffness Measures

The adopted total normal approach is

(5) $$\delta_{tot}=\delta_{b}+\delta_{l},\;\delta_{b}=\delta_{plateau}+\delta_{rmr}.$$

The *base approach* $\delta_{b}$ combines the statistical land-roughness approach $\delta_{plateau}$ and the deterministic Fourier displacement increment $\delta_{rmr}$. The separate increment $\delta_{l}$ is the coated-minus-bare periodic-cell response defined in Section 2.4. This decomposition makes the effects of summit engagement, texture geometry, and coating confinement individually accessible. In particular, the HS/FT comparison changes the asperity law while retaining the same summit population. Each component has a defined displacement reference and can be assessed under its prescribed boundary conditions. Their sum is an approximation because the associated pressure fields and displacement contributions can interact.

For the contact pair specified at the start of Section 2, the one-sided reduced modulus is $E^{*}=E/\left(1-\nu^{2}\right)$. The land roughness is prescribed through the characteristic inputs $R_{a}=0.28\;\mu$m and $R_{sm}=0.50$ mm. For a zero-mean Gaussian height distribution, the statistical closure gives $\sigma =R_{a}\sqrt{\pi /2}$, $R_{s}=R_{sm}^{2}/\left(4\pi^{2}\sqrt{2}\sigma \right)$, $\eta_{s}=R_{sm}^{-2}$, $z_{min}=-8\sigma$, and $z_{max}=3\sigma$. The variable *z* denotes summit height, and *d* denotes the separation of the counterface from the mean-height reference plane. Inverting the Greenwood–Williamson (GW) load integral [29] at $q_{land}=A_{q}q_{nom}$ gives the statistical land-roughness approach for the same summit population in the HS and FT branches. The pressure and area integrals use the Gaussian density over the truncated interval without renormalizing its tail; the contacting-summit fraction uses the normalization defined below.

For class A, let M be the periodic aperture indicator, $A_{cell}$ the nominal cell area, $\phi =1-F_{union}$, and $k_{mn}=\sqrt{k_{x}^{2}+k_{y}^{2}}$, where $k_{x}=2\pi m/L_{x}$ and $k_{y}=2\pi n/L_{y}$. Here, $L_{x}=p_{x}$ and $L_{y}=p_{y}$ are the axial and circumferential cell periods, respectively. The cell-normalized Fourier coefficients and the retained compliance length are

$${\hat{A}}_{mn}=\frac{1}{A_{cell}}\int_{A_{cell}}M\left(x,y\right)e^{-i(k_{x}x+k_{y}y)}\;dA,\;\Lambda_{N}=\frac{2}{\phi^{2}}\sum_{\begin{array}{c} |m|,|n|\leq N\\ \left(m,n)\neq (0,0\right) \end{array}}\frac{|{\hat{A}}_{mn}{|}^{2}}{k_{mn}}.$$

The sum uses the full signed square lattice, excluding the zero mode. The factor 2 follows from the one-sided normal half-space compliance $2/(E^{*}k)$ and is distinct from the counting of conjugate modes. At the prescribed cutoff, N=6, $\Lambda \equiv \Lambda_{6}=0.06036$ mm. With pressure and modulus in MPa, the displacement in micrometers is

$$\delta_{rmr}\left[\mu m\right]=1000\;\frac{q_{nom}\left[\mathrm{MPa}\right]\;\Lambda_{N}\left[\mathrm{mm}\right]}{E^{*}\left[\mathrm{MPa}\right]}.$$

The factor 1000 converts millimeters to micrometers. The stated compliance length and Fourier results refer to the prescribed finite-mode truncation. The zero mode determines the open-area fraction and land-pressure amplification. Class B uses the stripe-cell representation with $\delta_{rmr}=0$ in the adopted decomposition.

The Fourier increment defined above is retained in both contact branches; their distinction lies in the single-asperity law used for $\delta_{plateau}$. In the FT branch, the Hertz relation used in HS is replaced by the axisymmetric bonded-layer solution. With normal pressure and displacement taken positive into the stack, the transform convention is

(6) $$\tilde{p}\left(k\right)=\int_{0}^{a}p\left(r\right)J_{0}\left(kr\right)r\;dr,\;w\left(r\right)=\int_{0}^{\infty }C_{zz}\left(k\right)\tilde{p}\left(k\right)J_{0}\left(kr\right)k\;dk.$$

Here, $J_{0}$ is the Bessel function of the first kind of order zero, *a* is the radius of one asperity contact, and $a_{c}$ denotes the population-averaged contact radius. The compliance $C_{zz}$ follows from the elastic state-transfer solution of the coating/substrate stack. Displacements and tractions are continuous at the bonded interface, surface tangential traction vanishes, and both displacement components are fixed at the bottom of the local asperity stack. The periodic cell used to calculate $\delta_{l}$ has the separate bottom constraints specified in Section 2.4. For a homogeneous half-space, $C_{zz}\left(k\right)=2/\left(E^{*}k\right)$; for the fixed-bottom stack, $C_{zz}\left(0\right)=t_{l}/M_{l}+h_{s}/M_{s}$, where $M_{l}$ and $M_{s}$ are the constrained-strain moduli of the coating and substrate, respectively.

Explicitly, partition the transfer matrix by the displacement vector (U,W) and traction vector (T,S):

$$T\left(k\right)=e^{A_{l}\left(k\right)t_{l}}e^{A_{s}\left(k\right)h_{s}}=\left(\begin{array}{cc} T_{uu} & T_{ut}\\ T_{tu} & T_{tt} \end{array}\right),\;C_{zz}\left(k\right)=e_{2}^{T}T_{ut}\left(k\right)T_{tt}^{-1}\left(k\right)e_{2},$$

$$A\left(k\right)=\left(\begin{array}{cccc} 0 & k & 1/\mu & 0\\ -\lambda k/D & 0 & 0 & 1/D\\ 4\mu \left(\lambda +\mu \right)k^{2}/D & 0 & 0 & \lambda k/D\\ 0 & 0 & -k & 0 \end{array}\right),\;D=\lambda +2\mu ,\;e_{2}={\left(0,1\right)}^{T}.$$

In each layer, μ=G and λ=K−2G/3. Section S6.1 specifies the state convention, traction sign, and the numerical propagation-depth truncation used to evaluate the wavenumber-dependent kernel.

For a contacting spherical summit of radius $R_{s}$, the interference *s* and load *P* satisfy

(7) $$w\left(r\right)=s-\frac{r^{2}}{2R_{s}}\;\left(0\leq r\leq a\right),\;P=2\pi \int_{0}^{a}p\left(r\right)r\;dr,$$

with nonnegative pressure and no interpenetration outside contact. Section S6.1 specifies the discretization and elastic endpoint checks. Comparison with Yu et al. [30] tests the elastic single-asperity response over the stated modulus ratios.

The additive formulation evaluates the contributions separately under small-deformation, nonadhesive normal contact, perfect bonding, and the specified linear material response. The decomposition assumes limited overlap and weak pressure redistribution. The validity of roughness–texture scale separation remains an open question for this configuration. A fully coupled reference would determine common contact pressure and compatible normal displacements from one equilibrium boundary-value problem with matched geometry, material history, interfaces, external constraints, and displacement references. It would resolve the deformation fields and their scale interactions jointly. Sections S6.1 and S10.3 examine component sensitivities and specified pressure interactions; Section S11.1 examines pressure compatibility on smooth lands. The scope of the resulting coupling assessment is discussed in Section 4.2.

For material screening, the shear share of the instantaneous oedometric modulus is

(8) $$\zeta_{G}=\frac{4G_{0}}{3K+4G_{0}}.$$

The secant and tangent normal stiffnesses per unit nominal area are evaluated for each formulation as follows:

(9) $$K_{s}\left(q,t\right)=\frac{q}{\delta_{tot}\left(q,t\right)},\;K_{t}\left(q_{i},t\right)={\left[\frac{\delta_{tot}\left(q_{i+1},t\right)-\delta_{tot}\left(q_{i-1},t\right)}{q_{i+1}-q_{i-1}}\right]}^{-1}.$$

For the reported tangent values, the central difference uses $q_{\pm }=q\left(1\pm 0.005\right)$, so that $K_{t}=\left(q_{+}-q_{-}\right)/\left[\delta \left(q_{+}\right)-\delta \left(q_{-}\right)\right]$. The open-texture model is used only while

(10) $$\delta_{tot}\left(q_{nom},t\right)<h_{res};$$

the equality defines the class-specific closure pressure $q^{*}$.

For each texture class, elastic endpoint state, and contact branch, the contact regime is described by projected real area, contacting-summit fraction, areal density of contacting summits, and representative contact scales. Integrating the Gaussian summit-height density $f_{z}\left(z\right)$ over the prescribed height interval gives

$$\begin{array}{cccc} \frac{A_{r}}{A_{land}} & =\eta_{s}\int_{\max(d,z_{min})}^{z_{max}}\pi a^{2}\left(z-d\right)f_{z}\left(z\right)\;\;dz, & \frac{A_{r}}{A_{cell}} & =\left(1-F_{union}\right)\frac{A_{r}}{A_{land}},\\ f_{e} & =\frac{\int_{\max(d,z_{min})}^{z_{max}}f_{z}\left(z\right)\;\;dz}{\int_{z_{min}}^{z_{max}}f_{z}\left(z\right)\;\;dz}, & n_{c} & =\eta_{s}\int_{\max(d,z_{min})}^{z_{max}}f_{z}\left(z\right)\;\;dz,\\ a_{c} & =\frac{\int_{\max(d,z_{min})}^{z_{max}}a\left(z-d\right)f_{z}\left(z\right)\;\;dz}{\int_{\max(d,z_{min})}^{z_{max}}f_{z}\left(z\right)\;\;dz}, & \bar{s} & =\frac{\int_{\max(d,z_{min})}^{z_{max}}\left(z-d\right)f_{z}\left(z\right)\;\;dz}{\int_{\max(d,z_{min})}^{z_{max}}f_{z}\left(z\right)\;\;dz}. \end{array}$$

Here, $A_{land}=\left(1-F_{union}\right)A_{cell}$ is the load-bearing land area. The quantities $z_{min}$ and $z_{max}$ are the lower and upper support limits of the truncated summit population, respectively. The contacting-summit fraction is $f_{e}$, $n_{c}$ is the areal density of contacting summits, and $a_{c}$ is their number-weighted mean contact radius. The branch-specific local radius a(z−d) is obtained from the Hertz relation in the half-space branch and from the defined layered axisymmetric response in the finite-thickness branch. The dimensionless contact-regime indicators are

$$\chi_{a}=2a_{c}\sqrt{n_{c}},\;\chi_{s}=\frac{\bar{s}}{R_{s}},\;\chi_{E}=\frac{q_{land}}{E^{*}},\;\Pi_{q}=\frac{q_{nom}}{q_{branch,state}^{*}}.$$

The indicator $\chi_{a}$ compares the mean contact diameter $2a_{c}$ with the characteristic spacing $n_{c}^{-1/2}$ between contacting summits, whereas $\chi_{s}$ and $\chi_{E}$ characterize local indentation and stress. Together with $\Pi_{q}$, these quantities describe the contact population, local deformation scale, and proximity to branch-specific closure.

Additional dimensionless measures describe the coated-minus-bare contribution, finite-thickness effect, contact scale, and loading duration:

$$\Pi_{c}=\frac{\delta_{l}}{\delta_{b}},\;\Phi_{l}=\frac{\delta_{l}}{\delta_{tot}}=\frac{\Pi_{c}}{1+\Pi_{c}},\;\Pi_{h}=1-\frac{\delta_{plateau}^{FT}}{\delta_{plateau}^{HS}},\;\Pi_{a}=\frac{a_{c}}{t_{l}},$$

$$\Theta_{b}=\frac{t_{c}}{\tau_{ret,b}},\;\Theta_{l}=\frac{t_{c}}{\tau_{c,l}}.$$

The closure-proximity ratio $\Pi_{q}$ uses the closure pressure of the same branch and material state.

The indicator $\chi_{rmr}=\delta_{rmr}/\delta_{b}$ gives the deterministic Fourier share of the base approach, while $\Phi_{l}=\delta_{l}/\delta_{tot}$ gives the coated-minus-bare share of the total. Accordingly, $1-\Phi_{l}$ is the complete base share. The time ratio $\Theta_{b}=t_{c}/\tau_{ret,b}$ characterizes the HS quasi-elastic base approximation; the hereditary FT response is governed by the compliance memory defined in Section 2.6.

### 2.6. Characteristic Times and Loading Histories

The prescribed loading duration is $t_{c}=2a_{track}/v_{track}$, where $v_{track}$ is the prescribed speed and $a_{track}=\sqrt{a_{ax}a_{circ}}$ is the radius of a circle with the same area as the reference elliptical footprint. An actual path through that footprint can have a different length. For class A at $v_{track}=1.0$ m/s, $t_{c}=0.5093$ ms. At fixed temperature and material properties, speed changes this duration and the ratios $t_{c}/\tau$, $t_{c}/\tau_{ret,b}$, and $t_{c}/\tau_{c,l}$, while the relaxation and retardation times remain fixed. The histories below use this kinematic scale to prescribe the duration of loading at a fixed contact location.

The shear creep compliance for a held stress step is

(11) $$J_{G}\left(t\right)=\frac{1}{G_{\infty }}-\left(\frac{1}{G_{\infty }}-\frac{1}{G_{0}}\right)e^{-t/\tau_{ret,b}},\;\tau_{ret,b}=\tau \frac{G_{0}}{G_{\infty }}.$$

For the HS quasi-elastic approximation, the effective shear modulus ${\hat{G}}_{b}\left(t\right)=1/J_{G}\left(t\right)$ is converted using the elastic isotropic relations and inserted into the static contact law. The substitution matches both elastic endpoints. Hereditary evolution of the changing contact set is treated separately below. The normalized base progress is $P_{b}\left(t\right)=\left[\delta_{b}\left(t\right)-\delta_{b,0}\right]/\left[\delta_{b,\infty }-\delta_{b,0}\right]$.

The adopted scalar compliance for the coated-minus-bare increment is

(12) $$C_{l}\left(t\right)=C_{l,\infty }+\left(C_{l,0}-C_{l,\infty }\right)e^{-t/\tau_{c,l}},\;C_{l,j}=\frac{t_{l}\psi_{j}}{M_{j}},\;\tau_{c,l}=\tau \frac{M_{0}}{M_{\infty }}.$$

The scalar compliance $C_{l}\left(t\right)$ interpolates between $t_{l}\psi_{0}/M_{0}$ and $t_{l}\psi_{\infty }/M_{\infty }$ for the coated-minus-bare increment $\delta_{l}$, including the coating-induced change in substrate displacement. The single-exponential dependence adopts the constrained-strain retardation time $\tau_{c,l}$ as an approximation for this increment. Its relation to homogeneous constrained compression is given in Section S2; the local hereditary FT contact response is evaluated separately using the layer operator defined below. For a held land-pressure step $q_{land,h}$, $\delta_{l}^{hold}\left(t\right)=q_{land,h}C_{l}\left(t\right)$. Its normalized endpoint progress is

$$P_{l}\left(t\right)=\frac{C_{l}\left(t\right)-C_{l,0}}{C_{l,\infty }-C_{l,0}}=1-\exp\left(-t/\tau_{c,l}\right).$$

For a rectangular land-pressure pulse of duration $t_{c}$, linear superposition gives

(13) $$\delta_{l}^{pulse}\left(t\right)=q_{land,h}\left[C_{l}\left(t\right)-H\left(t-t_{c}\right)C_{l}\left(t-t_{c}\right)\right],$$

where *H* is the Heaviside function. Superposition applies to the adopted linear scalar law for $\delta_{l}$. Unloading of the nonlinear rough-contact population is calculated with the hereditary formulation below.

For the reference material, $\tau_{ret,b}=0.285$ ms and $\tau_{c,l}=0.259$ ms, giving $\Theta_{b}=1.786$ and $\Theta_{l}=1.970$. Table 2 reports the material-relaxation-time windows for which the corresponding exponential creep factor completes 5–95% of its change during the prescribed loading duration. These windows describe the effect of varying τ at fixed contact duration.

A separate local hereditary FT/GW calculation is performed at $q_{nom}=1$ MPa. Let

$${\tilde{G}}_{R}\left(s\right)=L\left\{G_{R}\left(t\right)\right\}\left(s\right)$$

denote the Laplace transform of the shear relaxation modulus, where *s* is the Laplace variable. The operational shear modulus is $\bar{G}\left(s\right)=s{\tilde{G}}_{R}\left(s\right)$:

(14) $$\bar{G}\left(s\right)=G_{\infty }+\left(G_{0}-G_{\infty }\right)\frac{s}{s+1/\tau },\;\bar{K}\left(s\right)=K.$$

These moduli enter the fixed-bottom stack operator $C_{zz}\left(k,s\right)$. The correspondence principle applies to this transformed spatial problem because its linear boundary conditions are fixed. The unilateral normal-contact region evolves at a fixed surface location and is determined in the time domain. A sum of retardation modes with nonnegative weights represents the compliance memory, and annular pressure active sets are solved at each time step. The Greenwood–Williamson load balance determines plane approach during held loading; contact reaction is calculated during prescribed withdrawal. This formulation allows the contact region to recede during unloading. Analytical viscoelastic-layer and rebound solutions provide methodological context [31]; the present calculation uses the coating/substrate properties and constraints specified above.

Two local unloading protocols follow held loading up to $t_{c}$. In protocol P (instantaneous retraction and pressure release), the plane is immediately retracted to −1μm relative to the initial highest summit, causing complete loss of contact and zero contact pressure; constitutive memory is retained. In protocol W (prescribed linear withdrawal), the plane is withdrawn linearly to the same position over 0.5τ=0.125 ms, with the contact reaction calculated throughout. Thus, W is displacement controlled during withdrawal. After detachment, the reported recovery is that of the local surface displacement; the plane follows its prescribed motion. Section S10.1 provides the numerical method and grid-specific event intervals. These histories apply at a fixed location. A translating pressure footprint requires the corresponding spatial and temporal loading history.

### 2.7. Finite-Element Verification Cell

The periodic finite-element cells use multipoint constraints on their lateral faces, fixed normal displacement at the substrate bottom, and a rigid, frictionless loading platen. The area-averaged normal approach ${\bar{u}}_{cell}$ characterizes the cell response used to determine the coated-minus-bare increment. Results are reported on the reference (medium) mesh and compared with a fine mesh; the figures use the labels reference and fine. Class A uses a three-dimensional periodic cell; class B uses a transverse plane section with a separate three-dimensional stripe comparison. For each class, six configurations are evaluated at five nominal pressures on both meshes. Appendix A defines the configurations and reports the mesh comparisons. Supplementary Section S6.1 gives the separate finite-thickness kernel checks, and Section S10.2 reports sensitivity to macroscopic boundary conditions.

These checks distinguish the cell response from the prescribed-traction layer response. The flat coated cell is compared with the oedometric reference, while the Navier/Fourier benchmark compares bonded-layer and one-direction compliances. Mesh comparisons report integral displacement and local pressure separately.

### 2.8. Numerical Implementation and Reproducibility

Monte Carlo propagation and Morris screening [32] use the one-sided HS reference formulation. Local topography sensitivity treats $R_{a}$ and $R_{sm}$ as primary descriptors and recomputes σ, $R_{s}$, $\eta_{s}$, and $z_{max}$ from the statistical closure. Central logarithmic derivatives use h=0.01, with checks at h=0.005 and 0.02. The HS and FT azimuthal comparisons use identical pressure fields, phases, amplitudes, and reference pressure. At the common azimuthal reference pressure, tangent stiffness is evaluated directly at $q_{\pm }=q_{0}\left(1\pm 0.005\right)$. The Supplementary Materials specify the input distributions and report the sensitivity and contact-regime results.

The elastic pressure-basis FT solver and the annular hereditary solver use the same coating/substrate parameters with different spatial discretizations. Their instantaneous and relaxed solutions are compared in Section S10.1. Each hereditary progress curve uses independently computed static endpoints on its own discretization, including the relaxed limit. Unloading retains the material memory and satisfies nonnegative pressure, nonpenetration and pressure–gap complementarity on annulus-averaged fields. Section S10.1 also reports spatial and temporal refinement and comparisons with elastic and viscoelastic analytical solutions.

Monte Carlo propagation uses 4000 samples from the prescribed input distributions, with a mean relaxation time of 250 μs. Morris screening uses 14 trajectories and 126 evaluations over a relaxation-time interval of 210–290 μs. These analyses characterize response variation under the specified input distributions. In the Morris variation of $F_{union}$, the Fourier compliance length is held fixed, so the result measures conditional sensitivity to land fraction. Recomputing aperture geometry would also change its Fourier coefficients. For these Monte Carlo and Morris analyses, the one-second dwell is much longer than the sampled retardation times, so the evaluated contact response is essentially relaxed. This explains the zero elementary effects of τ on the displacement and stiffness outputs in Table S12; time-scale ratios still depend on τ.

The supplementary datasets specify the geometry and Fourier convention defining $\Lambda_{N}$, the cited constitutive inputs, and the numerical comparisons. The accompanying scripts evaluate the reported analytical checks and statistics; the archive index identifies their inputs and outputs.

## 3. Results

### 3.1. Coating Confinement and Texture-Induced Edge Release

The prescribed geometry gives an open fraction $F_{union}=0.204$ and a land-pressure amplification $A_{q}=1.256$ for isolated imprints, compared with $F_{union}=0.0400$ and $A_{q}=1.04$ for the connected groove. The topological union prevents double counting in class A, whereas the connected class B topology is represented by a transverse section and checked by a separate three-dimensional stripe calculation. These class-specific open textures fix the land loading before the bonded-coating correction is evaluated.

For a given nominal pressure, texture geometry sets the land pressure, and coating confinement determines the associated through-thickness response. The flat bonded response is governed by the oedometric modulus. Texture edges locally relax the lateral restraint, and the texture factor ψ measures the resulting change in the coated-minus-bare increment. This response includes coating compression and the induced substrate-displacement change. For the reference geometries, isolated imprints have a larger texture factor than the continuous helical groove.

At a nominal pressure of 1MPa, the reference-discretization texture factors are 1.0297 and 1.0349 for class A and 1.0146 and 1.0151 for class B in the instantaneous and relaxed states, respectively. Relaxation lowers *G* at fixed *K*, raises the effective Poisson ratio, and strengthens the contrast between the constrained interior and the locally released edge. The relaxed factor therefore exceeds the instantaneous factor for both classes.

**Figure 3:**
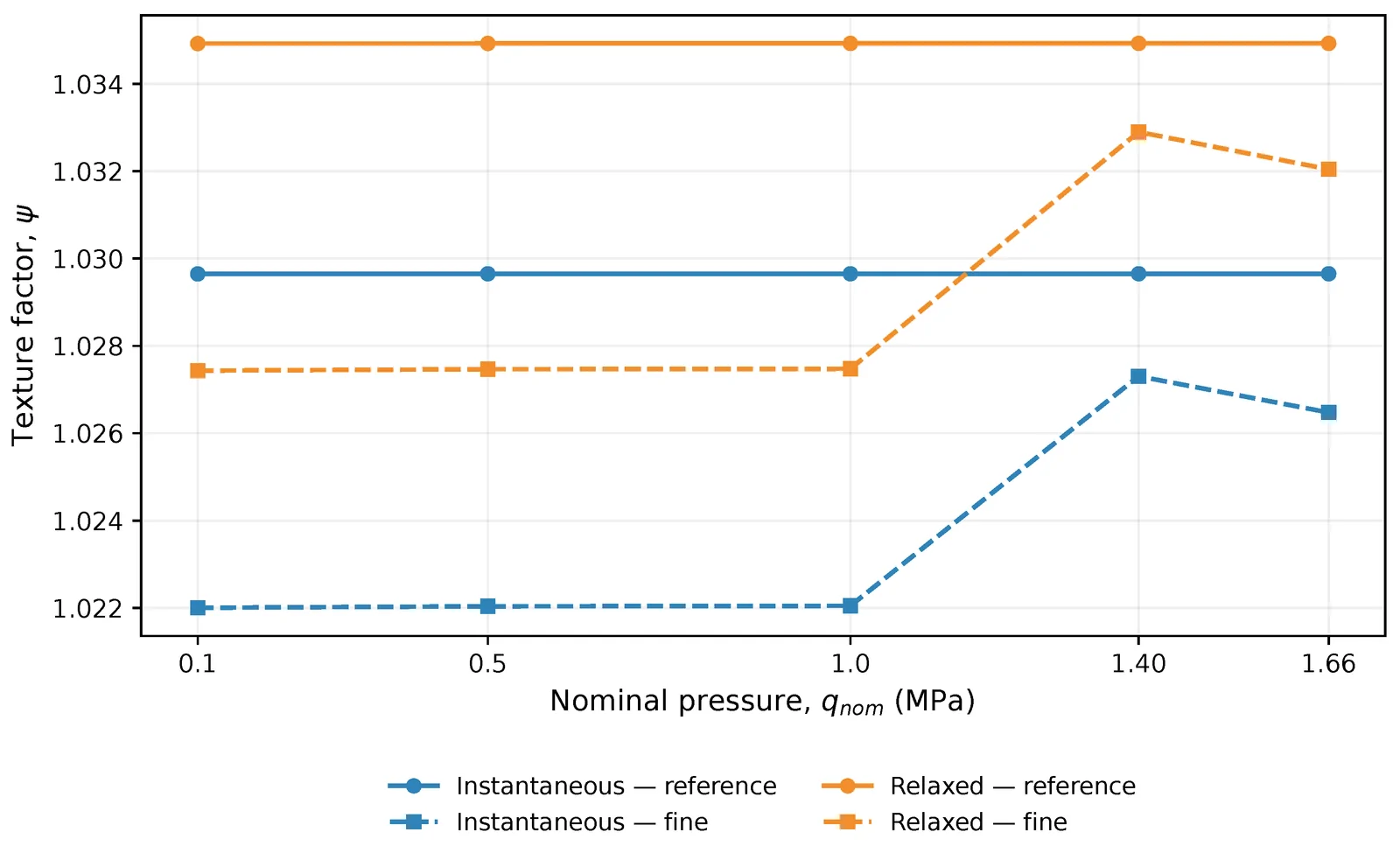
Pressure dependence of the class A texture factor at the sampled nominal pressures. Reference and fine FEM discretizations are shown for the instantaneous and relaxed states. The maximum relative reference-to-fine differences over these samples are 0.742% and 0.723%, respectively.

The maximum reference-to-fine differences in the texture factor over the sampled nominal-pressure levels are 0.742% and 0.723% for class A and 0.187% and 0.183% for class B in the instantaneous and relaxed states, respectively. The fine-mesh texture factors vary slightly and nonmonotonically at the two higher sampling pressures. Figure 3 shows the complete pressure dependence.

**Figure 4:**
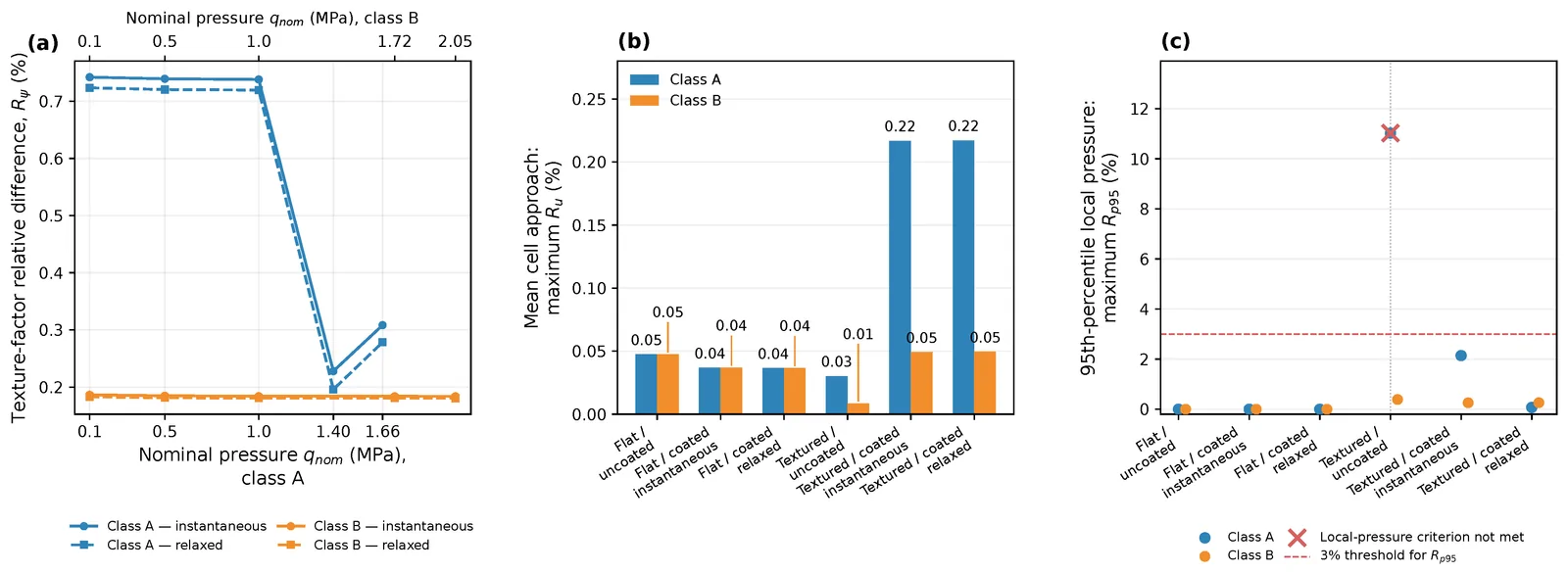
Reference-to-fine differences for the periodic FEM cells. (**a**) Texture-factor difference $R_{\psi }$ at the class-specific nominal pressures; lower and upper ticks identify the class A and class B samples. (**b**) Mean cell-approach difference $R_{u}$ and (**c**) normalized pressure-percentile difference $R_{p95}$, each maximized over the five sampled pressures for the indicated variant. The metrics are $R_{\psi }=100\left|\psi_{ref}-\psi_{fine}\right|/\psi_{ref}$, $R_{u}=100|{\bar{u}}_{cell,ref}-{\bar{u}}_{cell,fine}\left|/\right|{\bar{u}}_{cell,fine}|$, and $R_{p95}=100|{\hat{p}}_{95,ref}-{\hat{p}}_{95,fine}\left|/\right|{\hat{p}}_{95,fine}|$, where ${\hat{p}}_{95}=p_{95}/q_{ref}$. The area-weighted percentile $p_{95}$ is normalized by the mean active-contact pressure for textured cells and by nominal pressure for flat cells. The textured, uncoated class A family exceeds the local-percentile criterion at every sampled pressure.

Integral displacement and local pressure show different mesh sensitivities. Figure 4 compares mesh differences in the texture factor, mean cell approach, and normalized 95th percentile of local contact pressure; Appendix A gives the numerical mesh comparisons. The textured, uncoated class A family exceeds the 3% local-percentile criterion at all five sampled pressures and is marked separately.

### 3.2. Two Texture Classes

At $q_{nom}=1$ MPa, the reference-cell texture factors are 1.0297 and 1.0349 for instantaneous and relaxed class A, compared with 1.0146 and 1.0151 for class B. Their land-pressure amplification factors are 1.256 and 1.042, respectively. The relaxed HS closure pressures are 3.739 MPa for class A and 4.746 MPa for class B; the instantaneous closure pressures are 4.156 and 5.275 MPa. For class B, the signed stripe-minus-plane-section difference in area-averaged coated-cell approach is +0.00686%, with absolute relative error 0.00686%<0.01% under matched conditions (Section S1.4, Table S5).

**Figure 5:**
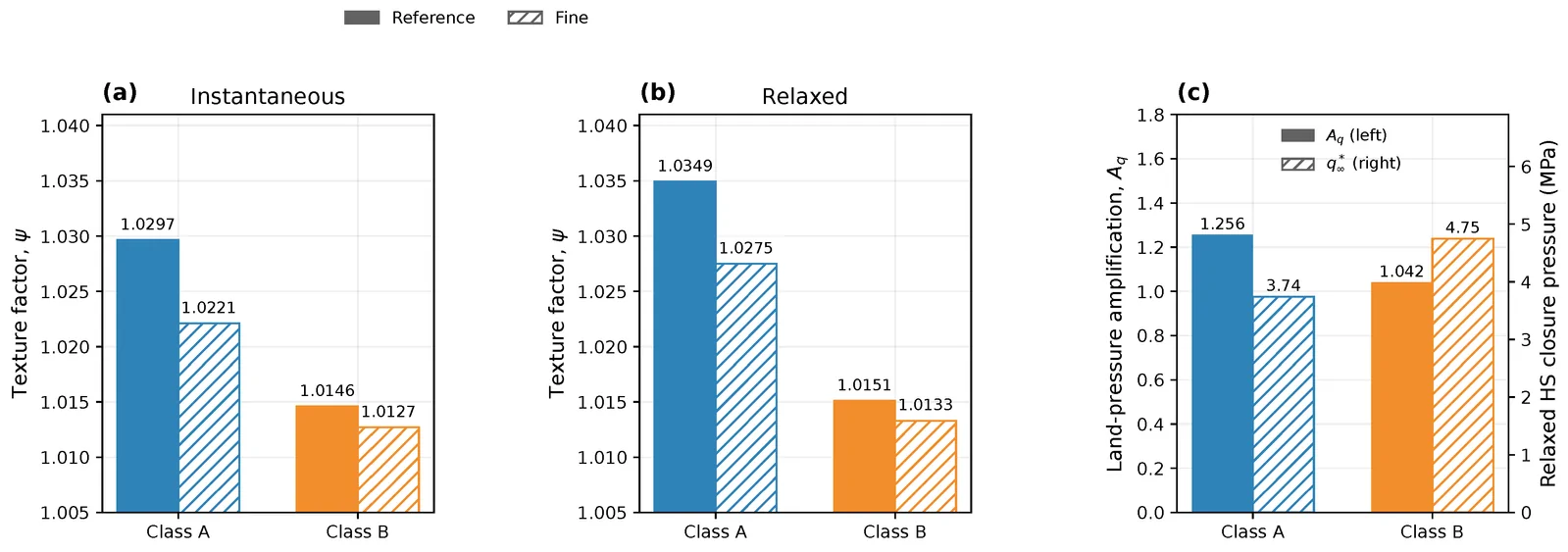
Comparison of texture classes A and B. (**a**,**b**) Instantaneous and relaxed FEM texture factors at $q_{nom}=1\;\mathrm{MPa}$ on the reference and fine discretizations. (**c**) Land-pressure amplification $A_{q}$ (left axis) and relaxed HS closure pressure $q_{\infty }^{*}$ (right axis). The class A and B closure pressures are approximately 3.74 and 4.75MPa, respectively.

Figure 5 compares the two classes, and Table 3 summarizes their reference quantities.

### 3.3. Contributions to the Instantaneous-to-Relaxed Change

We distinguish the relative total endpoint increase from the progress reached during a finite dwell:

$$S_{\delta }=100\frac{\delta_{tot,\infty }-\delta_{tot,0}}{\delta_{tot,0}},\;S_{b}=100\frac{\delta_{b,\infty }-\delta_{b,0}}{\delta_{tot,0}},\;S_{l}=100\frac{\delta_{l,\infty }-\delta_{l,0}}{\delta_{tot,0}}.$$

Thus, $S_{\delta }=S_{b}+S_{l}$, with $S_{\delta }$ expressed in percent and the contributions in percentage points.

**Figure 6:**
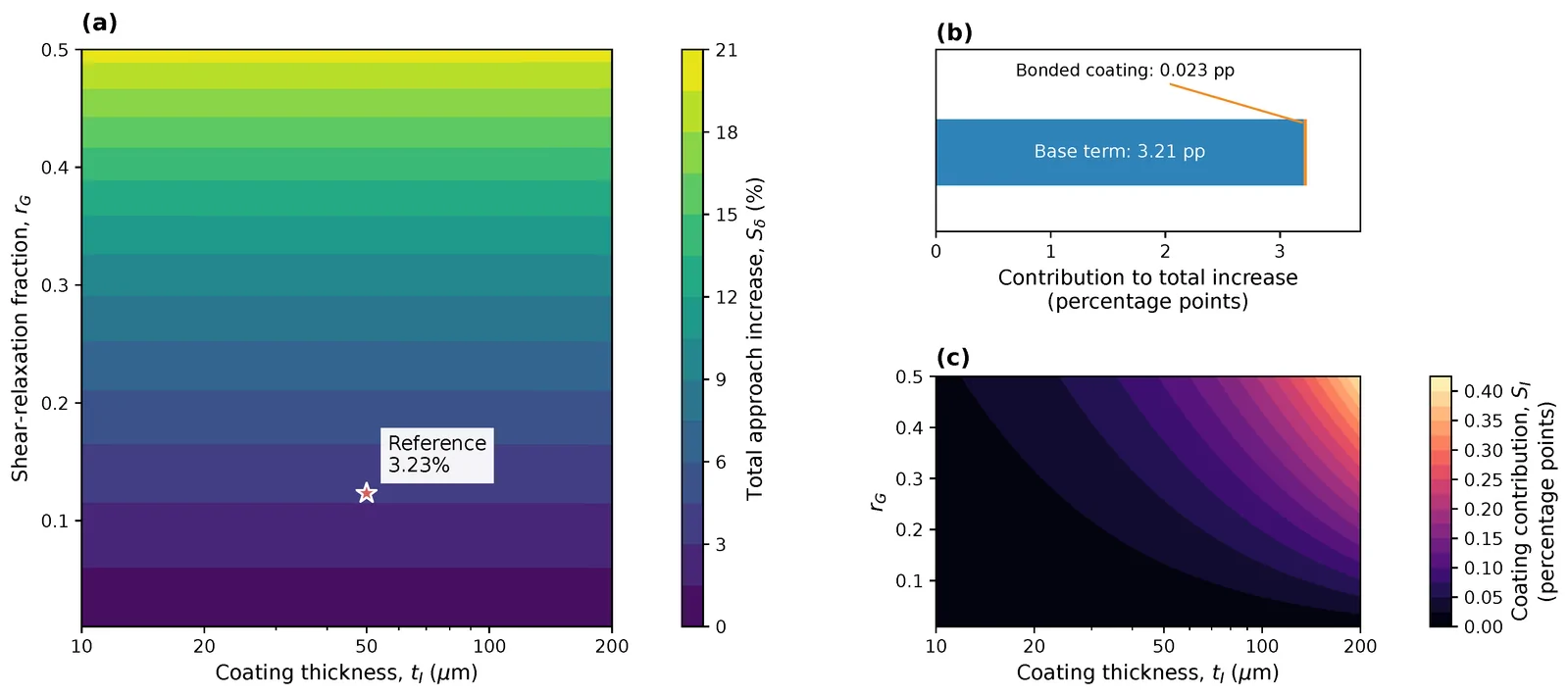
HS endpoint-change map for class A at $q_{nom}=1\;\mathrm{MPa}$ and bulk modulus K=5000MPa. (**a**) Total approach increase $S_{\delta }$ as coating thickness and shear-relaxation fraction vary. (**b**) Reference decomposition: $S_{\delta }=3.23\%$, with 3.21 percentage points from the base term and 0.023 percentage points from the coating. (**c**) Coating contribution over the same scan. All contributions use the initial total approach as denominator; the base is $\delta_{b}=\delta_{plateau}+\delta_{rmr}$. The scan uses the stated constitutive and texture-factor interpolation assumptions. The star in (**a**) marks the reference case, $t_{l}=50\;\mu m$ and $r_{G}=\left(G_{0}-G_{\infty }\right)/G_{0}≃0.123$.

Figure 6 shows the HS endpoint-change map and its component contributions.

**Figure 7:**
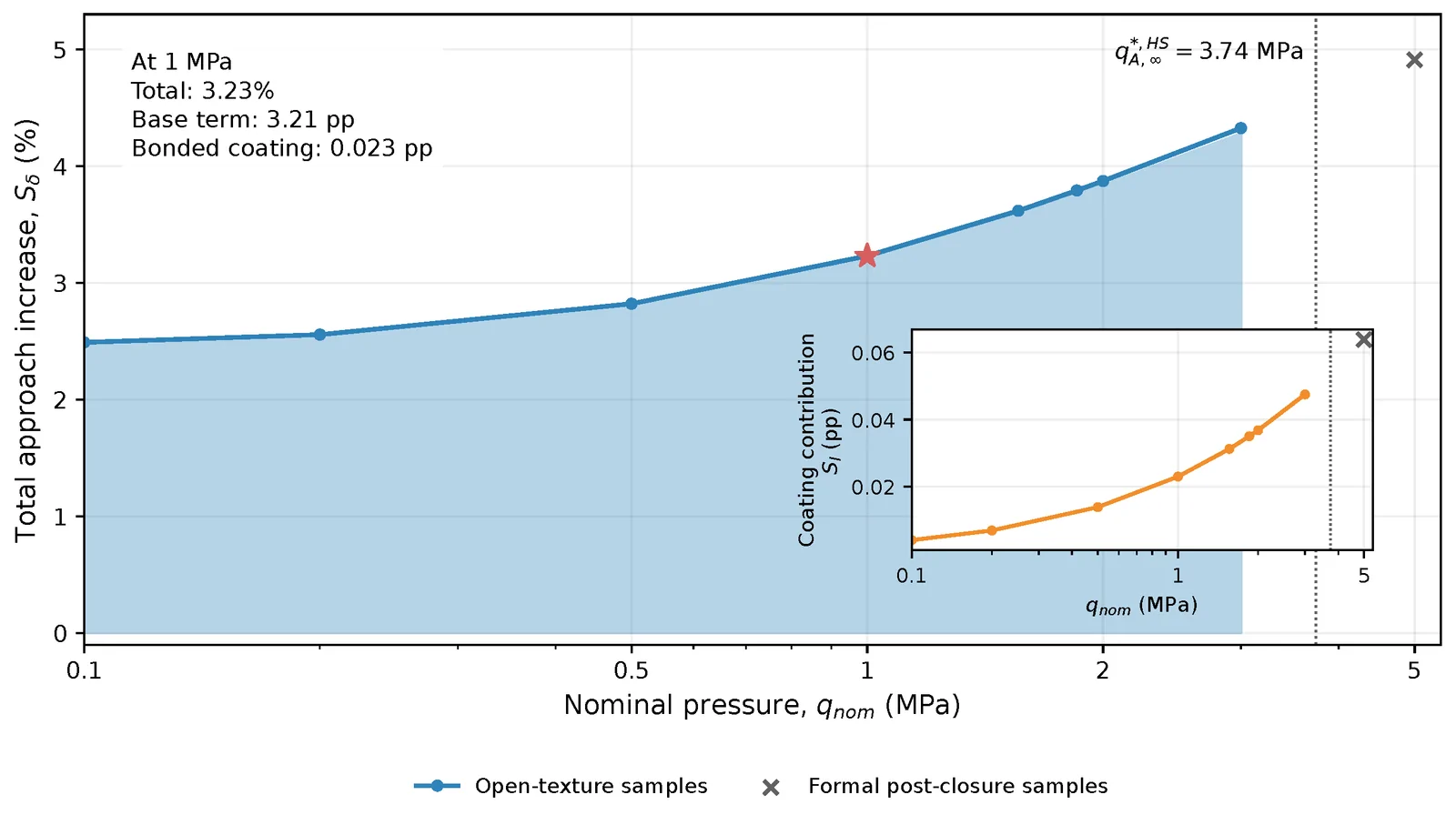
Instantaneous-to-relaxed approach increase for class A in the HS formulation. Symbols denote evaluated pressure samples; connecting lines guide the eye. The shaded contribution is the base term $\delta_{b}=\delta_{plateau}+\delta_{rmr}$, and the inset shows the coating contribution in percentage points, both normalized by the instantaneous total approach. The vertical line marks relaxed closure at $q_{A,\infty }^{*}=3.74\;\mathrm{MPa}$. Crosses denote formal post-closure continuation. The star marks the reference sample at $q_{nom}=1\;\mathrm{MPa}$.

In HS, changing summit engagement gives sublinear statistical approach versus compliance: $\Delta \ln\delta_{plateau}/\Delta \ln\left(1/E^{*}\right)=0.2872$ at 1 MPa with the one-sided modulus convention. This contributes to the weak pressure dependence of the endpoint span in Figure 7, which separates the total and coated-minus-bare increases.

**Figure 8:**
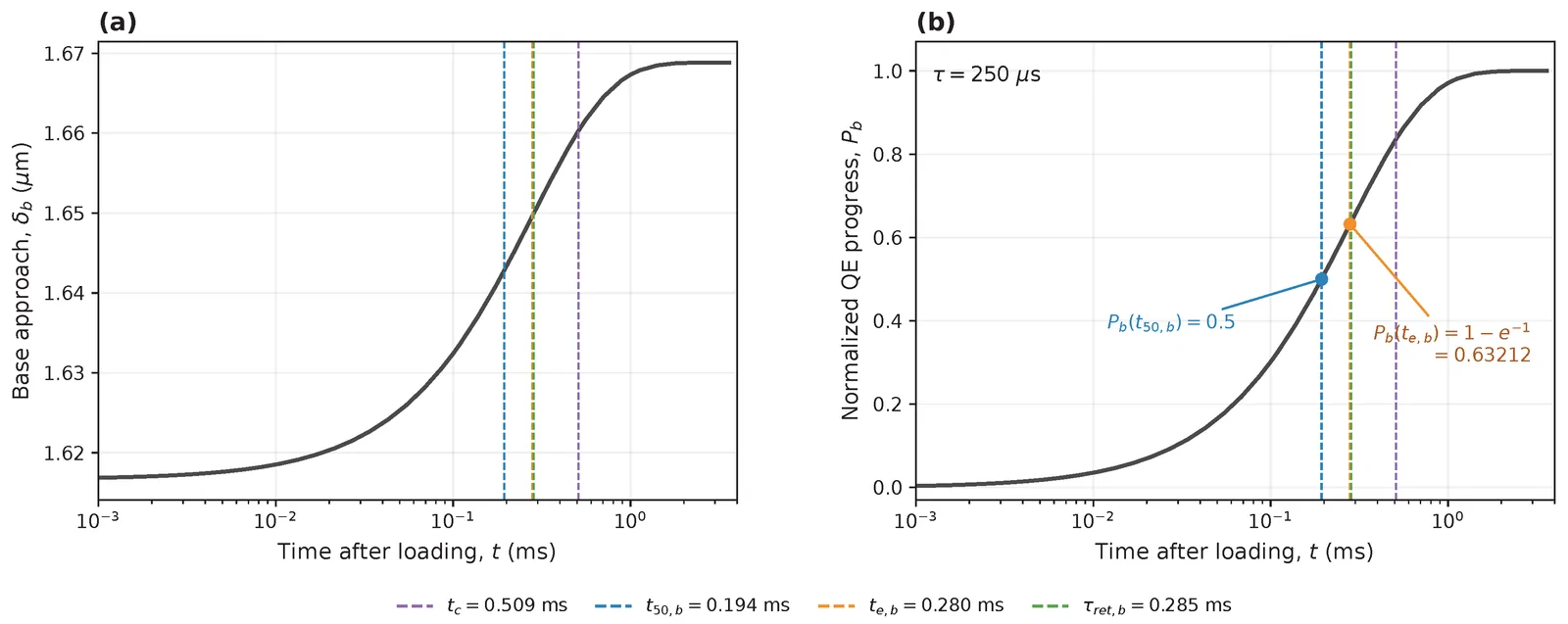
Quasi-elastic HS history for class A at $q_{nom}=1\;\mathrm{MPa}$, Λ=0.0604mm, and τ=250μs. (**a**) Base approach $\delta_{b}=\delta_{plateau}+\delta_{rmr}$. (**b**) Endpoint progress, with blue and orange markers at the half-transition and e-folding times defined in Section 3.3. The nonlinear compliance–approach relation makes the e-folding time differ from the shear-creep retardation time $\tau_{ret,b}=0.2851$ ms.

**Figure 9:**
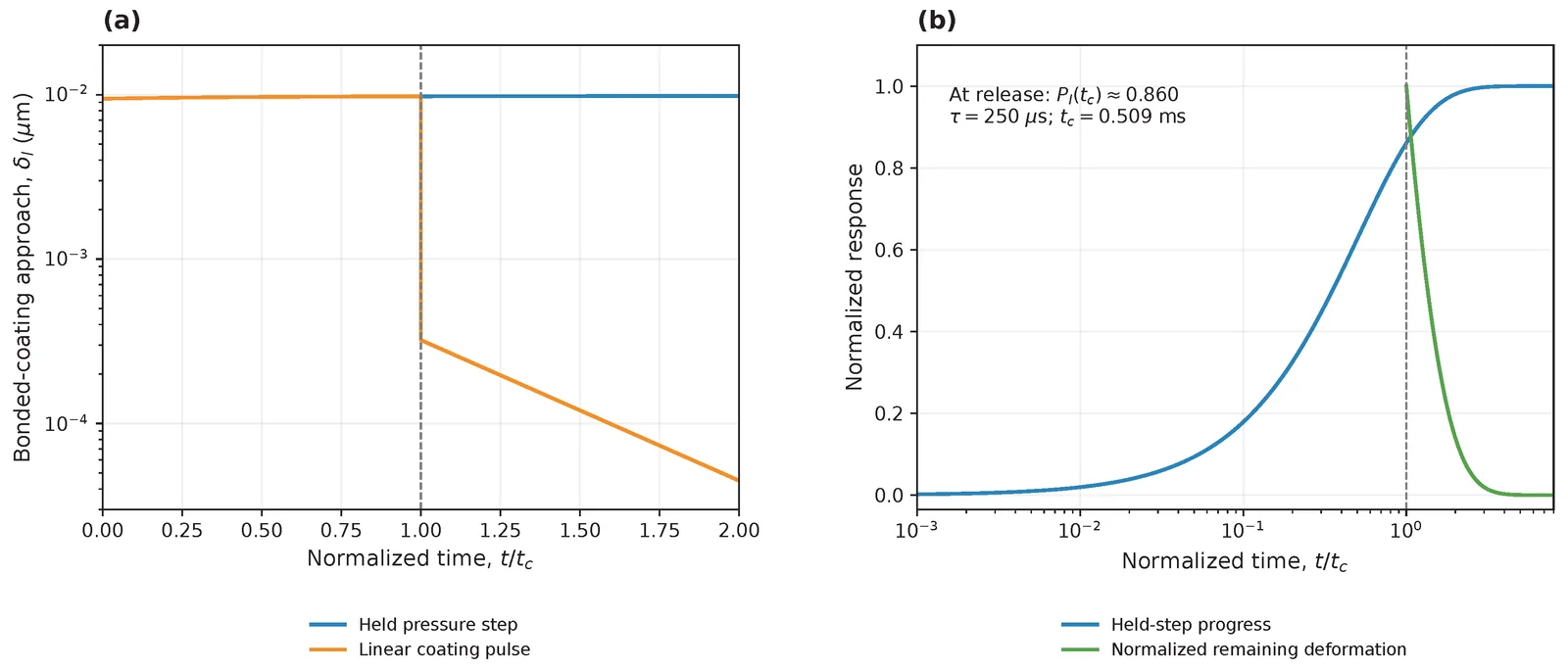
Adopted scalar coated-minus-bare cell response at $q_{nom}=1\;\mathrm{MPa}$ and τ=250μs. (**a**) Increment $\delta_{l}$ under a held land-pressure step and a rectangular land-pressure pulse released at $t_{c}=0.509\;\mathrm{ms}$. The increment includes the coating-induced change in substrate displacement. (**b**) Held-step endpoint progress and remaining pulse deformation normalized by its value immediately after release. The held-step progress at release is approximately 86.0%. The single-exponential law interpolates between the cell endpoints; local hereditary FT contact unloading is evaluated separately.

Pressure samples above the relaxed HS closure pressure of 3.739 MPa are marked as formal continuations; connecting lines guide the eye between evaluated samples. Figure 8 shows the HS quasi-elastic history for τ=250μs. The base half-transition time is $t_{50,b}=0.1937$ ms, defined by $P_{b}\left(t_{50,b}\right)=0.5$; the endpoint-deficit e-folding time is $t_{e,b}=0.2802$ ms, defined by $1-P_{b}\left(t_{e,b}\right)=e^{-1}$. At $t_{c}=0.509$ ms, the base has completed approximately 83.6% of its endpoint change, while total approach has increased by 2.70% from its instantaneous value. The two percentages describe progress through the endpoint interval and growth relative to the initial total, respectively. For the separate coated-minus-bare contribution, Figure 9 shows the held-step and finite-pulse responses obtained from the adopted scalar compliance in Section 2.6, with $\tau_{c,l}=0.2586$ ms.

### 3.4. Finite-Thickness Rough-Contact Check

At 1 MPa, the class A instantaneous total approach is 1.626 μm in the HS branch and 1.139 μm in the elastic FT branch; the relaxed values are 1.679 and 1.154 μm. The relative endpoint increase decreases from 3.23% in HS to approximately 1.27% in FT. Table 4 separates the statistical plateau, deterministic Fourier increment, and coated-minus-bare increment and reports the corresponding secant and tangent stiffnesses.

The base-to-coating endpoint-change ratio is 139.45 in HS and 37.70 in FT. This approximately 3.70-fold reduction quantifies the effect of the single-asperity law within the adopted component partition. In the relaxed state, the coated-minus-bare increment contributes approximately 0.585% of total approach in HS and 0.852% in FT. The Fourier increment contributes approximately 0.915% of the HS base and 1.334% of the FT base. Statistical land roughness therefore accounts for most of the base deformation in this reference case.

For both reference texture classes in the FT branch, the open-texture condition $\delta_{tot}\left(q_{nom},t\right)<h_{res}$ holds at all evaluated states and pressures in 0.1–5 MPa. The pressure samples cover both elastic endpoint states; their largest total approach is 1.741 μm, below $h_{res}=2.702\;\mu$m. Table S17 lists the closure pressures. Continuing the open-texture law gives formal closure pressures above 17 MPa for class A and above 27 MPa for class B. These values mark extrapolated limits of that law; the cavity-closure and post-closure contact problem is not solved. The prescribed multiplier range in Table S18 quantifies sensitivity to the additive Fourier term.

The hereditary FT calculation gives the held-step history at 1 MPa on its annular discretization (Section S10.1). The initial and relaxed adopted totals on that discretization are 1.139 and 1.154 μm. At $t_{c}$, the statistical land-roughness approach is 1.127 μm and the adopted total is 1.151 μm. The base has completed 83.99% of its endpoint change, corresponding to a 1.07% increase in the total. The Supplementary Materials compare the static endpoints obtained with the pressure-basis and annular discretizations; the transient landmarks use the annular endpoints consistently.

### 3.5. Secant and Tangent Stiffness

The evaluated pressure–response samples in the open-texture domain show sublinear growth of both secant and tangent stiffness with nominal pressure. Total approach grows sublinearly with pressure. Tangent stiffness exceeds secant stiffness at each sampled state. Both stiffness measures decrease from the instantaneous to the relaxed endpoint at matched pressure. These trends hold for the two classes in both HS and FT formulations over the reported open-contact samples. Supplementary Table S3 reports the HS values, and Section S6.1 gives the FT comparison.

For relaxed class A in HS at 1 MPa, the local logarithmic sensitivities of $\delta_{tot}$, $K_{s}$, and $K_{t}$ to $R_{a}$ are approximately +0.699, −0.699, and −0.456; those to $R_{sm}$ are +0.286, −0.286, and −0.494. Each perturbation recomputes the dependent summit parameters from the statistical closure. Table S20 and Figure S1 report these six sensitivities and their dependence on perturbation size.

### 3.6. Approach Decomposition, Closure, and Finite Contact Pulse

The open-texture domain requires $\delta_{tot}<h_{res}$. The closure pressure satisfies $\delta_{tot}\left(q^{*}\right)=h_{res}$, with $h_{res}=2.702\;\mu$m for class A. Table 4 (upper block) lists the HS/FT component endpoints at 1 MPa. Supplementary Table S3 gives the pressure-dependent HS values, Table S16 compares HS/FT plateau approaches, and Table S17 identifies closure pressures and formal continuations. Figure 9 shows the held-step and finite-pulse responses of the adopted scalar coated-minus-bare increment, including the change in substrate displacement. At release, the held-step progress is $P_{l}\left(t_{c}\right)=86.0\%$. The remaining pulse deformation is normalized by its value immediately after release, giving a recovery measure specific to the pulse.

The pulse in Figure 9 concerns the scalar coated-minus-bare increment. The local rough-contact response during unloading is obtained separately from the hereditary FT formulation in Section S10.1, which evolves the contact region while retaining material memory. Just before release, at $t_{c}^{-}$, the contact area is 3.816% of nominal area. In protocol P, the maximum local surface displacement is 0.04571 μm immediately after pressure removal and decreases to approximately $1.02\times 10^{-6}\;\mu$m after a further 12τ. In protocol W, contact reaction falls to zero during prescribed withdrawal. On the plotted grid of 384 annuli and 480 summit-height nodes, detachment occurs in (0.571772,0.574897] ms; the Supplementary Materials also report temporal refinement. These histories describe reversible local viscoelastic recovery with prescribed counterface motion.

### 3.7. Azimuthal Pressure-to-Stiffness Response

The azimuthal comparison uses three prescribed harmonic pressure fields,

$$q_{nom}\left(\theta \right)=q_{0}\left[1+\varepsilon \cos\left(n\theta +\alpha \right)\right],\;q_{0}=0.8q_{A,\infty }^{*,HS}=2.991\;\mathrm{MPa}.$$

The labels hub/cam, mandrel-tooth, and three-roller ovality denote illustrative patterns with (ε,n,α)=(0.10,1,0), (0.05,12,0.30), and (0.15,2,0.15), respectively, with phases in radians. The pressure fields are prescribed inputs to a comparative calculation; actual tool or cylinder equilibrium is outside this loading model. The common pressure keeps all branches below the most restrictive closure scale. Even the largest modulation spans only 0.68–0.92 of the relaxed class A HS closure pressure.

The relative stiffness spread is $S_{K}=200\left(K_{s,max}-K_{s,min}\right)/\left(K_{s,max}+K_{s,min}\right)$. For relaxed class A, the three spreads are 11.95%, 5.96%, and 18.00% in HS and 14.70%, 7.34%, and 22.11% in FT. Table S23 extends the comparison to both texture classes and both elastic endpoints. The ordering is unchanged across all eight class/state/branch combinations. For small modulations, the local transfer factor $d\ln K_{s}/d\ln q=1-K_{s}/K_{t}$ quantifies how the nonlinear contact law maps pressure variation into secant-stiffness variation. Figure S2 summarizes the spreads, and Table S26(a) gives the reference transfer quantities.

### 3.8. Material Screening and Prescribed-Distribution Results

**Figure 10:**
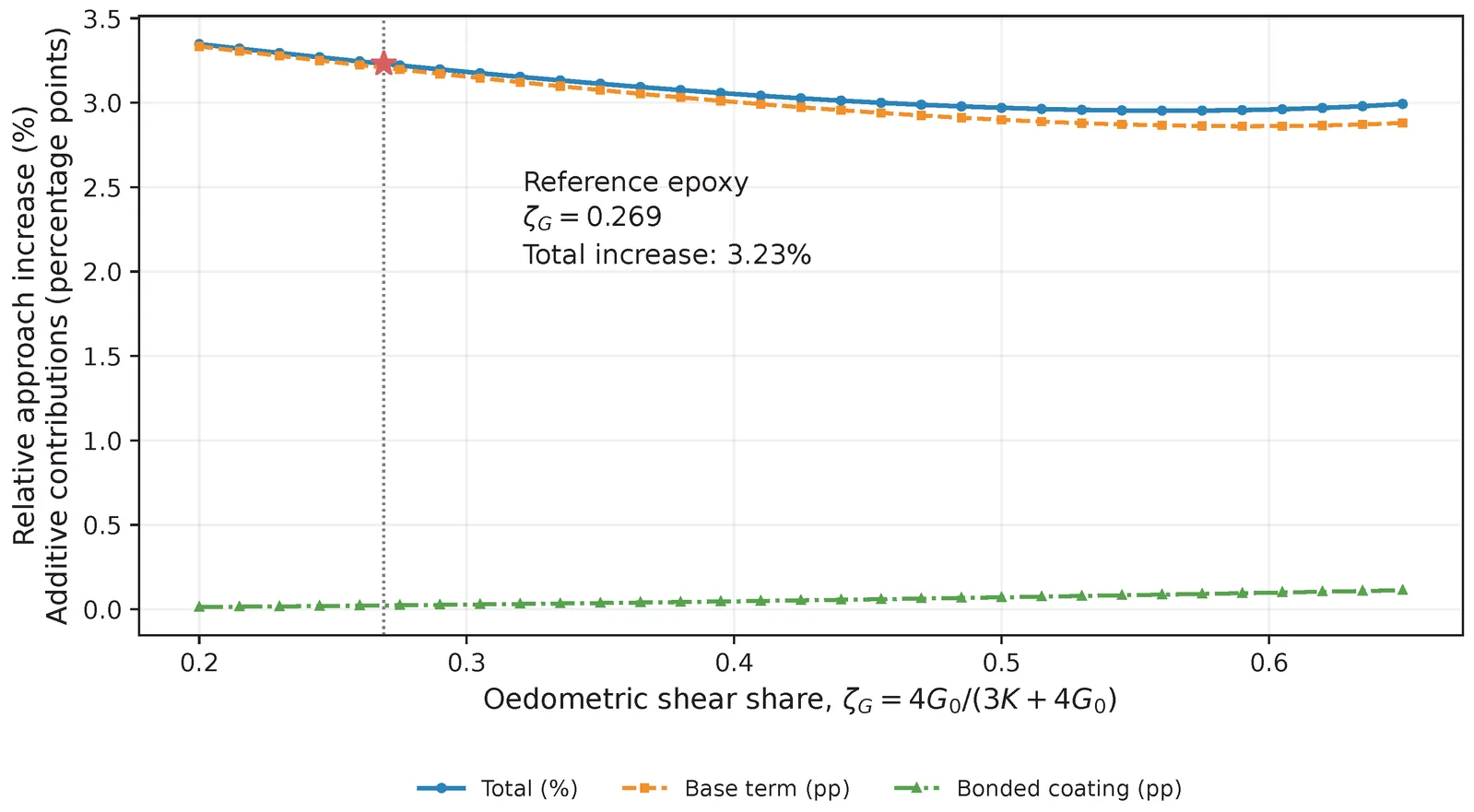
Material-response scan for class A in the HS formulation at $q_{nom}=1\;\mathrm{MPa}$. The total endpoint increase and additive base/coating contributions are shown as bulk modulus *K* varies at fixed $G_{0}=1380\;\mathrm{MPa}$, $G_{\infty }=1210\;\mathrm{MPa}$, and $t_{l}=50\;\mu m$. The horizontal coordinate is $\zeta_{G}=4G_{0}/\left(3K+4G_{0}\right)$. All contributions use the instantaneous total approach as denominator, and the base includes both plateau and Fourier terms. The reference total increase is approximately 3.23%.

Figure 10 shows the HS material scan as bulk modulus varies at fixed shear endpoints and coating thickness. The coordinate $\zeta_{G}=4G_{0}/\left(3K+4G_{0}\right)$ measures the shear share of the instantaneous oedometric modulus. The coated-minus-bare contribution is small over the reference parameter range, while the total endpoint increase and its partition depend on the formulation. Table 2, Figure 8 and Figure 9, and the hereditary FT histories in Figure S3 describe the corresponding time scales and responses.

The scan covers $0.20\leq \zeta_{G}\leq 0.65$. At fixed shear endpoints, varying *K* changes the instantaneous Poisson ratio according to $\nu_{0}=\left(2-3\zeta_{G}\right)/\left(4-3\zeta_{G}\right)$. The nonauxetic range ends at $\zeta_{G}=2/3$, so the selected interval remains within that range.

For the prescribed input distributions, the relaxed HS total-approach median is 1.683 μm, with 5th and 95th percentiles of 1.640 and 1.739 μm. The corresponding secant-stiffness percentiles are approximately 0.575, 0.594, and 0.610 MPa/μm. The median coated-minus-bare share is 0.00585. The 5th, median, and 95th percentiles of $\Theta_{l}$ are approximately 1.51, 1.98, and 2.57. These ranges describe propagation of the specified input variation. Section S4 reports the Morris effects and the parameters held fixed in their interpretation.

For normalized shear-relaxation progress $R_{G}\left(t\right)=\left[G_{0}-G_{R}\left(t\right)\right]/\left(G_{0}-G_{\infty }\right)$, equal Prony weights at τ/3 and 3τ preserve the elastic endpoints and change $t_{50,G}/\tau$ from 0.693 to 0.580. At the reference loading duration, the one-term and two-term spectra complete approximately 87.0% and 74.5% of the shear-relaxation change. These results characterize $R_{G}\left(t\right)$; the hereditary FT contact histories use the stated one-term material law.

## 4. Discussion

### 4.1. Mechanistic Interpretation of the Two Contributions

The component partition distinguishes how summit engagement, deterministic texture, and coating confinement contribute to normal approach. The coated-minus-bare increment includes the induced change in substrate displacement, so its interpretation extends beyond compression of the coating alone.

The class A formulation has the expected limiting responses. As $F_{union}\to 0$, $A_{q}\to 1$ and $\delta_{rmr}$ vanishes, so the base approach reduces to the statistical land-roughness approach. As $t_{l}\to 0$, the coated-minus-bare increment vanishes. At fixed $h_{s}$, the layered asperity kernel tends to that of a homogeneous elastic layer of thickness $h_{s}$ with both displacement components fixed at the bottom and zero tangential traction at the top (Section S6.1). The Hertz half-space additionally requires $h_{s}/a\to \infty$. The instantaneous-to-relaxed changes vanish as $G_{0}\to G_{\infty }$, and the response approaches its instantaneous endpoint as $t_{c}/\tau \to 0$. Supplementary Table S14 assesses the coating-removal limit of the spectral compliance on a fixed wavenumber grid. Table S15 compares load and approach with Hertz at finite homogeneous depth. These comparisons distinguish sampled spectral convergence from the full contact limit as $t_{l}\to 0$, which requires a separate assessment. Lateral restraint makes the bulk-modulus term dominant in $M_{oed}\left(G\right)=K+4G/3$, limiting the fractional change of the constrained coating response as shear modulus relaxes. Texture edges modify the restraint and substrate displacement, producing ψ>1. Figure 2 illustrates the resulting local deformation profiles and their mesh sensitivity. Its intermediate rise concerns the normalized compression defined in Section 2.4; the physical origin of the complete nonmonotonic sequence remains unresolved. Progressive summit engagement stiffens the rough-contact law as pressure rises. The lower base-to-coating endpoint-change ratio in the FT branch (Section 3.4) reflects the effect of substrate confinement on the base approach. This ratio depends on roughness, load, coating/substrate stiffness contrast, contact radius relative to thickness, texture geometry, and the adopted partition.

The component partition in Section 3.4 shows that statistical land roughness supplies most of the deformation, while the Fourier increment contributes only about 1% of the base approach at the class A reference point. Texture affects several contributions through the open fraction, land-pressure amplification, and texture factor. The contacting-summit fractions and spacing indicators in Table S24 place the HS/FT comparison in the context of the same prescribed population and its independent-asperity assumption.

The prescribed loading duration is comparable to the retardation times. The hereditary FT history in Supplementary Section S10.1 progresses rapidly over a small endpoint interval, giving only a small increase in total approach. The unloading histories in Section 3.6 resolve local surface recovery under prescribed plane motion. Speed acts through the duration-to-material-time ratios; translating contact and free counterface motion require the corresponding loading history and equations of motion.

Comparison with Yu et al. tests the elastic single-asperity kernel within the stated conditions. Layered-contact studies such as Dong et al. [33] and analytical viscoelastic-layer solutions such as Argatov [31] provide context for resolving interfaces and memory. Together, these studies motivate the component methods. Predictive assessment of a particular coated surface requires measured coating properties, land topography, and loading conditions.

The laser-pitted joint experiments of Fan and Zhou [34] provide qualitative context for texture-dependent stiffness. Their pressure–approach reference subtracts specimen bulk compression, and remelted rims affect the response. A quantitative comparison with the coated model would require matched material properties and land-roughness data.

### 4.2. Limitations and Scope

Statistical asperity descriptions represent rough-surface separation through a prescribed summit population [29,35]. The contacting-summit fraction and spacing indicator describe that population and help assess the applicability of the independent-asperity approximation, whose neglect of interactions limits their interpretation [36].

The quantitative results apply to a local planar patch with small deformations, an independent-asperity population, perfect bonding, frictionless normal contact, and an open texture. Nominal pressure and statistical roughness are prescribed inputs. Their use for an internal cylinder requires a separate description of macroscopic deformation and service loading.

The macroscopic boundary-condition matrix quantifies sensitivity to lateral restraint. At 1 MPa, freeing both mean lateral strains changes the refined instantaneous platen-to-bottom displacement by 36.2% relative to the matched zero-lateral-strain periodic reference. The coated-minus-bare increment changes by 5.78% in that comparison; its largest change across the evaluated matrix is 5.85%. These sensitivities concern the specified cell observables under paired boundary conditions. Section S10.2 defines the observables, boundary conditions, and meshes.

Intercomponent coupling is the principal unresolved limitation of the additive construction. The possible overlap and pressure redistribution between components are discussed in Section 2.5. The construction is useful for controlled comparisons because statistical summit engagement, deterministic aperture compliance, and coating/substrate confinement have distinct physical roles, and their component formulations have separate numerical checks (Sections S1.3, S6.1, and S8). The Fourier-amplitude study and one-way redistribution calculation address prescribed sensitivities; the two-way split-operator calculation depends on the chosen spectral division and modal resolution. A general coupling-error assessment would require a sufficiently resolved, fully coupled reference satisfying the common-pressure and displacement-compatibility conditions defined in Section 2.5 (Section S10.3). The macroscopic BC matrix concerns a separate sensitivity to lateral restraint.

The hereditary histories describe local FT/GW contact. During held loading, the additive total combines that response with separately specified Fourier and coating operators; after detachment, the calculation reports local surface recovery. The HS time-dependent scans use the quasi-elastic approximation. Mesh comparisons assess integral responses and local pressure-related measures separately. The normalized pressure-percentile mesh differences for uncoated textured class A exceed the stated criterion, limiting conclusions about local pressure convergence. The azimuthal and mechanical-series comparisons are conditional on their prescribed pressure patterns and component inputs; applying them to a particular assembly requires corresponding loading and stiffness data.

The overall model assumes nonadhesive quasistatic contact and omits asperity interactions and frictional heating.

### 4.3. Model Verification and Experimental Comparison

Numerical verification addresses the elastic and hereditary components separately from their additive combination. In the layered spherical-contact benchmark, the normalized contact radius and peak pressure agree with approximate graphical values from Yu et al. [30] within 1.5%. Periodic-cell mesh refinement changes the mean approach by less than 0.22%, while a matched three-dimensional stripe calculation supports the section representation of class B. Local pressure remains more sensitive to resolution. The prescribed-traction Navier benchmark and the closed-form homogeneous-layer solution [23] provide complementary assessments of the displacement operator. Fourier-cutoff comparisons assess the deterministic displacement increment, and local hereditary FT loading and withdrawal tests assess material memory and the evolving contact region. Analytical ramp convolution provides a separate reference for finite loading times.

Pressure redistribution is examined in a smooth-land model with a fixed traction-free aperture. Enforcing constant normal displacement on the lands reduces its deterministic compliance by about 25% relative to uniform land pressure. For the class A elastic endpoints at $q_{nom}=1$ MPa, substituting this component alone changes the total secant stiffness by less than 0.33%. The component contributes little to total approach under these endpoint conditions, limiting its effect on total stiffness. This is a component-substitution sensitivity for the specified aperture model. Supplementary Section S11.1 and Table S31 give the observables, discretizations, and error definitions for these comparisons.

Indentation experiments connect material characterization to contact response through the applied loading history and the quantity inferred from it. Oyen [37] compared indentation-derived shear moduli with independently obtained comparison values. For the stiff-material class comprising PL-1, cured Epon, and dry gelatin, the mean absolute relative difference is approximately 11.4%, with a maximum of 18.2%. Section S11.2 and Table S32 report all four materials, including the physically distinct soft urethane rubber and its larger discrepancy. The comparison values were obtained by different methods; these differences characterize the reported modulus pairs.

Confinement affects both the deformation and the modulus relevant to its interpretation. For the compressible film studied by Gacoin et al. [38], the constrained modulus calculated from the published bulk properties is approximately 2.57 GPa, compared with 2.8 GPa inferred from contact, a descriptive difference of about 8.9%. This agreement supports a constrained-strain reference for local film compression under the stated confinement and interface conditions (Section S11.3 and Table S33). In the present formulation, the coated-minus-bare increment $\delta_{l}$ additionally includes the change in substrate displacement caused by the coating.

A related thickness effect appears in the spherical indentation experiments of Cheneler et al. [39]: thinner polydimethylsiloxane (PDMS) layers required larger forces at matched indentation depth and composition. The HS/FT difference reported in Section 3.4 shows the same direction of stiffening. Differences in material, support conditions, and loading protocol restrict the experimental comparison to the thickness trend (Section S11.4).

Interface conditions also influence thin-film compliance. Johnson and Stafford [40] found that contact-based estimates for cross-linked methacrylate films depended on confinement and interfacial adhesion. Section S11.4 and Table S34 retain the reported geometry, compliance estimates, and uncertainties for lauryl methacrylate with 75% by mass of 1,6-hexanediol dimethacrylate. These observations emphasize the role of the interface alongside the bulk material response. A matched prediction requires the corresponding force and loading history, together with an interface model appropriate to the experiment; reduced adhesion differs from the perfect bonding assumed here.

The influence of loading history is illustrated by the flat-punch measurements of Herbert et al. [41]. For plasticized polyvinyl chloride (PVC), a viscoelastic representation identified from frequency-domain measurements described separately measured creep over approximately 0.1–10 s. This transfer between loading histories supports hereditary evaluation over a specified experimental window. Application to the present submillisecond coating response requires the appropriate material spectrum and contact conditions. Section S11.5 and Table S35 give the published coefficients and the scope of the comparison.

The increase of interface stiffness with load provides a further qualitative comparison. Fuadi [42] observed increasing normal stiffness for four soft-material surfaces, with a surface ranking that changed with load. The rough cast-iron joint measurements reproduced by Yu et al. [43] also show increasing stiffness with load, consistent with progressive contact engagement in the present rough-contact branches. Fuadi used local force–displacement loop slopes, whereas the cast-iron joint stiffness was inferred from resonance measurements. Quantitative comparison with the present nominal-area secant stiffness $K_{s}$ or tangent stiffness $K_{t}$ therefore requires matching the displacement reference, loading protocol, and area normalization (Section S11.6 and Tables S36–S37).

A quantitative interface comparison is available for the saturated normal stiffness of uncoated micropatterned polycarbonate measured by Perris et al. [44]. The present two-body periodic finite-element reduction uses the reported elastic properties and ridge dimensions for all four patterned cases, with their common additive compliance baseline set by a single flat-interface measurement. The model reproduces the increase in stiffness with ridge width, with a mean absolute relative error of approximately 20.1% across all four specimens. The largest error, about 58.9%, occurs for the narrowest ridge, 80.6 μm wide: the prediction is 124.1 kN/mm, compared with the reported 78.1±4.0 kN/mm (Section S11.7 and Table S38).

The Perris comparison depends on how displacement is observed, because digital image correlation (DIC) measures deformation in both bodies over a finite gauge. The numerical reduction approximates the five experimental target pairs by a spatially averaged gauge observation. The experimental stiffness of the narrowest ridge lies below the full range obtained by varying the sampling position; the tested geometric sensitivity leaves its physical cause unresolved. The comparison assesses an uncoated two-body elastic interface under its stated calibration and observation assumptions. Taken together, the literature comparisons support specific material, confinement, and stiffness trends. Quantitative validation of the complete coated rough-contact response requires matched material data, geometry, loading history, and displacement measurements.

## 5. Conclusions

At the class A reference point of 1 MPa, statistical land roughness dominates the normal approach. The deterministic Fourier increment contributes only about 1% of the base approach, and the coated-minus-bare increment is below 1% of the total approach. Finite thickness reduces both absolute approach and its relaxation-induced increase.

Texture topology affects the land-pressure amplification and texture factor as well as the deterministic Fourier increment. Isolated imprints have a larger texture factor than the connected groove, and relaxation increases this factor for both classes at the reference pressure. The pressure dependence of stiffness reflects progressive summit engagement: over the sampled open-contact states, secant and tangent stiffness increase sublinearly with pressure, tangent stiffness exceeds secant stiffness, and relaxation reduces both.

Both texture classes remain within the open-texture domain in the FT branch over the investigated 0.1–5 MPa interval. Under the same prescribed azimuthal fields, FT produces larger stiffness modulation while preserving the ranking of the three illustrative pressure patterns across classes and elastic endpoints.

During a fixed-location hold of 0.509 ms, the hereditary FT base approach develops approximately 84% of its endpoint change. Because that endpoint interval is small, the total approach increases by only about 1% relative to its instantaneous value. The local FT histories resolve surface recovery after instantaneous retraction and during finite withdrawal. These results apply to the prescribed local loading protocols; a translating pressure footprint requires its spatial and temporal loading history.

The contribution of the framework is a component-level comparison of roughness, deterministic texture, and coating confinement, with intercomponent coupling identified as the principal open question. Predictive application to a specific coated cylindrical contact calls for measured topography and constitutive spectra, representative loading and macroscopic constraints. A resolved common-pressure reference would quantify coupling, and matched loading–unloading experiments would assess the combined response.

## Figures and Tables

**Table 1:** Material parameters, isotropic endpoints, and derived scales of the reference model. The parameters define a prescribed, literature-based material idealization.

Quantity	Symbol	Value	Basis
Bulk modulus	*K*	5000 MPa	Constant bulk modulus
Instantaneous shear modulus	$G_{0}$	1380 MPa	Prescribed endpoint
Relaxed shear modulus	$G_{\infty }$	1210 MPa	Prescribed endpoint
Material relaxation time	τ	250 μs	Literature-based one-term idealization
Coating thickness	$t_{l}$	50 μm	Perfectly bonded coating
Substrate parameters	$E_{s};\nu_{s};h_{s}$	72 GPa; 0.33; 2.70 mm	Prescribed isotropic substrate
Instantaneous elastic endpoints	$E_{0};\nu_{0}$	3791.2 MPa; 0.37363	Isotropic conversion
Relaxed elastic endpoints	$E_{\infty };\nu_{\infty }$	3359 MPa; 0.38803	Isotropic conversion
Shear-modulus decrease	ΔG	170 MPa	$G_{0}-G_{\infty }$
Constrained modulus	$M_{\mathrm{oed}}\left(G\right)$	K+4G/3	Constant-K SLS
Coating retardation time	$\tau_{c,l}$	0.2586 ms	$\tau M_{0}/M_{\infty }$
QE base retardation scale	$\tau_{\mathrm{ret},b}$	0.2851 ms	$\tau G_{0}/G_{\infty }$
Class A loading duration	$t_{c}$	0.5093 ms	$2a_{\mathrm{track}}/v_{\mathrm{track}}$ at 1 m/s
Land roughness	$R_{a};R_{sm}$	0.28 μm; 0.50 mm	Prescribed descriptors
Class A geometry factors	$F_{\mathrm{union}};A_{q}$	0.2037; 1.256	Ellipse/cell geometry
Class A Fourier compliance length	Λ	0.06036 mm	Full signed N = 6 lattice; one-sided Green factor 2

**Table 2:** Characteristic times for class A. The base transition follows the stated HS quasi-elastic law; the cell increment follows the adopted scalar law. The material-time windows describe the exponential factors in these two laws.

Quantity	HS Quasi-Elastic Base	Adopted Scalar Increment	Unit
Material relaxation time	0.250	0.250	ms
Prescribed loading duration	0.5093	0.5093	ms
Retardation scale	0.285	0.259	ms
Half-transition time	0.194	0.179	ms
E-folding time	0.28	0.259	ms
$t_{c}$/retardation scale	1.79	1.97	dimensionless
τ window for 5–95% exponential factor	0.149–8.71	0.164–9.6	ms

**Table 3:** Texture-class comparison at 1 MPa. The plane-section/stripe-cell comparison uses matched instantaneous loading and boundary conditions.

Quantity	Class A	Class B	Interpretation
Open fraction $F_{union}$	0.204	0.0400	Topological open texture in the periodic unit cell
Land-pressure amplification $A_{q}$	1.256	1.04	$A_{q}={\left(1-F_{union}\right)}^{-1}$
Cell representation	Three-dimensional periodic cell	Plane section across the groove	Class-specific geometric representations
ψ, instantaneous	1.0297	1.0146	Reference-discretization texture factor
ψ, relaxed	1.0349	1.0151	Reference-discretization texture factor
Relaxed HS closure pressure	3.7386MPa	4.75MPa	$q_{A/B,\infty }^{*,HS}$ of the open-texture model
Signed relative difference of 3D from 2D	—	+0.00686%	$100\left({\bar{u}}_{cell,3D}-{\bar{u}}_{cell,2D}\right)/{\bar{u}}_{cell,2D}$

**Table 4:** Additive class A elastic endpoint responses at 1 MPa (upper block) and instantaneous-to-relaxed changes (lower block). The FT rows use the pressure-basis layered-asperity endpoint discretization. Both endpoint-change contributions use the same instantaneous total as denominator; $S_{\delta }=S_{b}+S_{l}$. Here, pp denotes percentage points.

**Branch**	**State**	$\delta_{\mathrm{plateau}}$ **(** μ **m)**	$\delta_{\mathrm{rmr}}$ **(** μ **m)**	$\delta_{b}$ **(** μ **m)**	$\delta_{l}$ **(** μ **m)**	$\delta_{\mathrm{tot}}$ **(** μ **m)**	$K_{s}$ **(MPa/** μ **m)**	$K_{t}$ **(MPa/** μ **m)**
HS	Inst.	1.603	0.01370	1.617	0.00945	1.626	0.615	2.09
HS	Rel.	1.654	0.01526	1.669	0.00983	1.679	0.596	1.98
FT	Inst.	1.116	0.01370	1.130	0.00945	1.139	0.878	3.87
FT	Rel.	1.128	0.01526	1.144	0.00983	1.154	0.867	3.81
**Branch**		$\Delta \delta_{b}$ **(μm)**	$\Delta \delta_{l}$ **(μm)**	$S_{b}$ **(pp)**	$S_{l}$ **(pp)**	$S_{\delta }$ **(%)**	$\Delta \delta_{b}/\Delta \delta_{l}$	
HS		0.052158	0.00037403	3.2075	0.023001	3.23	139.45	
FT		0.014103	0.00037403	1.2381	0.032837	1.27	37.704	

**Figure A1:**
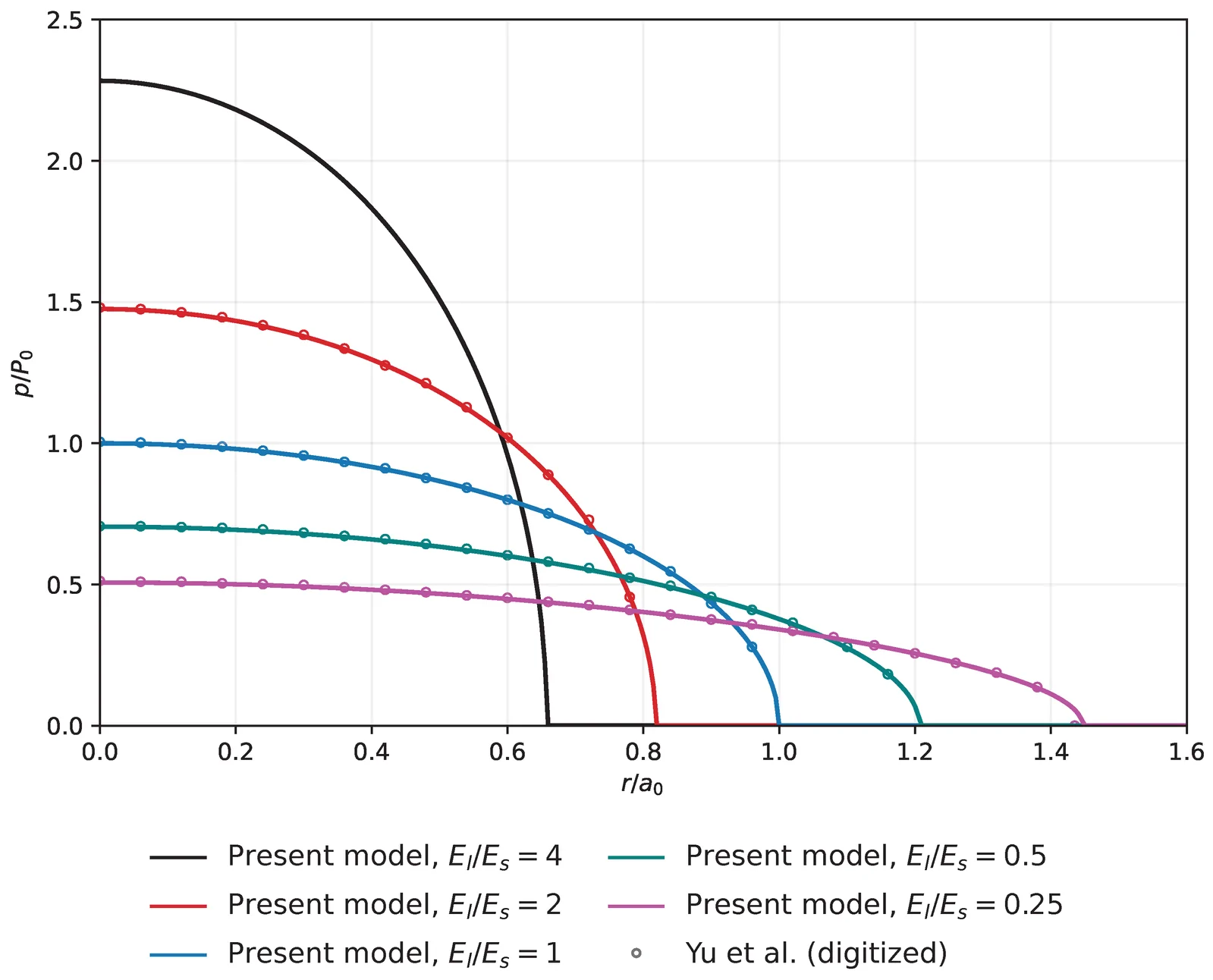
Normalized pressure-profile comparison for the frictionless single-layer cases of Yu et al. (2014) [30]. The Hertz radius of the homogeneous substrate at load *P* is $a_{0}$, and its peak pressure is $P_{0}=2E_{s}^{*}a_{0}/\left(\pi R\right)$, where $E_{s}^{*}=E_{s}/\left(1-\nu_{s}^{2}\right)$. Solid curves show the present calculation over $E_{l}/E_{s}=0.25$–4; open markers approximate Figure 3 of Yu et al. [30] for $E_{l}/E_{s}=0.25$, 0.5, 1, and 2. Table S25 compares contact radius and peak pressure.

## Data Availability

The original contributions presented in this study are included in the article and Supplementary Material. Further inquiries can be directed to the corresponding author.

## Supplementary Materials

The following supporting information can be downloaded at https://www.mdpi.com/article/10.3390/polym18212638/s1: Supplementary Materials: geometry and material inputs, displacement references, numerical methods, verification and sensitivity analyses, and published experimental comparisons (Sections S1–S11, Tables S1–S38, and Figures S1–S4). Supplementary Archive: numerical datasets and eight scripts for analytical checks and statistical summaries. References [21,23,29,30,32,35,36,37,38,39,40,41,42,43,44] are cited in the Supplementary Materials.

## Abbreviations and Nomenclature

Abbreviation		Definition
SLS		Standard linear solid
FEM		Finite-element method
HS		Half-space formulation
FT		Finite-thickness formulation
QE		Quasi-elastic approximation
GW		Greenwood–Williamson statistical contact model
BC		Boundary condition
Symbol	Unit	Definition
$A_{q}$	–	Land-pressure amplification, ${\left(1-F_{union}\right)}^{-1}$
$A_{r}$	mm^2^	Projected real contact area predicted by the defined summit population
$a_{c}$	μm	Number-weighted mean contact radius
$E^{*}$	MPa	One-sided reduced modulus for the coated internal cylindrical surface against a rigid counterface
$f_{e}$	–	Contacting-summit fraction within the truncated population
$F_{union}$	–	Periodic open-area fraction of the deterministic texture
$n_{c}$	mm^−2^	Areal density of contacting summits
$q_{nom},q_{land}$	MPa	Nominal pressure and mean pressure on the load-bearing lands
$q^{*}$	MPa	Branch- and state-specific closure pressure
$q_{0}$	MPa	Common azimuthal evaluation pressure defined from the relaxed class A half-space closure
$R_{a}$	μm	Prescribed arithmetic mean profile height of the load-bearing lands
$R_{sm}$	mm	Prescribed profile-element spacing of the land-roughness closure
$R_{s}$	mm	Mean summit-curvature radius of the prescribed closure
$S_{x}^{y}$	–	Local logarithmic sensitivity of output *y* to primary descriptor *x*
$S_{\delta }$	%	Relative increase in total approach, $100\left(\delta_{tot,\infty }-\delta_{tot,0}\right)/\delta_{tot,0}$
$S_{b},S_{l}$	pp	Base and coated-minus-bare endpoint changes, each divided by $\delta_{tot,0}$ and multiplied by 100
$S_{K}$	%	Relative secant-stiffness spread, $200\left(K_{s,max}-K_{s,min}\right)/\left(K_{s,max}+K_{s,min}\right)$
${\bar{u}}_{cell}$	μm	Area-averaged normal approach of the periodic FEM cell
$\sigma_{EE}$	–	Standard deviation of the normalized Morris elementary effects
$\bar{s}$	μm	Mean summit interference over contacting summits
$t_{l}$	μm	Coating thickness
$\delta_{b}$	μm	Base approach, $\delta_{plateau}+\delta_{rmr}$, combining statistical land roughness and deterministic Fourier displacement
$\delta_{l}$	μm	Coated-minus-bare periodic-cell displacement increment, including the induced substrate-displacement change
$\delta_{tot}$	μm	Total normal approach
$\delta_{plateau}$	μm	Statistical land-roughness approach of the prescribed summit population
$\eta_{s}$	mm^−2^	Areal summit density of the prescribed closure
ϕ	–	Load-bearing land fraction, $1-F_{union}$
ψ	–	Texture factor for the cell-averaged coated-minus-bare displacement increment
ξ	–	Normalized elliptical radial offset from the class A imprint boundary (dimensionless)
σ	μm	Standard deviation of zero-mean Gaussian heights derived from $R_{a}$
$G_{R}\left(t\right)$	MPa	Shear relaxation modulus
$J_{G}\left(t\right)$	MPa^−1^	Shear creep compliance for a held stress step
${\hat{G}}_{b}\left(t\right)$	MPa	Effective creep-derived modulus $1/J_{G}\left(t\right)$ used in the HS quasi-elastic approximation
$G_{0},G_{\infty }$	MPa	Instantaneous and relaxed shear-modulus endpoints
$h_{res}$	μm	Prescribed residual texture depth and geometric closure scale
*K*	MPa	Bulk modulus of the coating parameter set
$M_{oed}\left(G\right)$	MPa	Oedometric modulus at shear modulus *G*, K+4G/3
$t_{c}$	s	Class A loading duration derived from the equivalent-area radius and prescribed speed $v_{track}$
$P_{b},P_{l}$	–	Held-step progress of each contribution relative to its own endpoint change
$t_{50,b},t_{e,b}$	s	HS quasi-elastic base times at $P_{b}=0.5$ and $P_{b}=1-e^{-1}$, respectively
$t_{50,G}$	s	Half-transition time of shear relaxation, $\left[G_{0}-G_{R}\left(t_{50,G}\right)\right]/\left(G_{0}-G_{\infty }\right)=0.5$
$\tau ,\tau_{c,l},\tau_{ret,b}$	s	Material relaxation time, scalar coating retardation time and HS quasi-elastic base retardation scale; displayed in ms or μs where stated
$r_{G}$	–	Shear-relaxation fraction, $\left(G_{0}-G_{\infty }\right)/G_{0}$
$\zeta_{G}$	–	Shear share of the instantaneous oedometric modulus, $4G_{0}/\left(3K+4G_{0}\right)$
$K_{s},K_{t}$	MPa/μm	Secant and tangent normal stiffness per unit nominal area.
$K_{eq}$	N/μm	Incremental secant series stiffness for roller redistribution
$P_{pre}$	N	Preload force in the mechanical-series illustration
$\Pi_{c}$	–	Coated-minus-bare increment relative to the base approach, $\delta_{l}/\delta_{b}$
$\Phi_{l}$	–	Coated-minus-bare share of total approach at a specified state, $\delta_{l}/\delta_{tot}$
$\Pi_{h}$	–	Relative finite-thickness correction, $1-\delta_{plateau}^{FT}/\delta_{plateau}^{HS}$
$\Pi_{a}$	–	Contact-radius-to-coating-thickness ratio, $a_{c}/t_{l}$
$\Pi_{q}$	–	Branch- and state-specific closure proximity, $q_{nom}/q^{*}$
$\Theta_{b},\Theta_{l}$	–	Prescribed loading duration divided by the HS quasi-elastic base and scalar cell-increment retardation scales, respectively
$\chi_{a}$	–	Mean contact diameter relative to contacting-summit spacing, $2a_{c}\sqrt{n_{c}}$
$\chi_{s}$	–	Mean-interference ratio, $\bar{s}/R_{s}$
$\chi_{E}$	–	Land-pressure ratio, $q_{land}/E^{*}$
$R_{G}\left(t\right)$	–	Normalized shear-relaxation progress, $\left[G_{0}-G_{R}\left(t\right)\right]/\left(G_{0}-G_{\infty }\right)$ The comparison of spectra uses this material-response measure
$\delta_{rmr}$	μm	Deterministic Fourier displacement increment
$\delta_{coat}^{loc}$	μm	Local normal compression across the coating thickness
$\Lambda_{N}$	mm	Fourier compliance length of the aperture mask at the stated full signed cutoff *N*
$\chi_{rmr}$	–	Fourier share of base approach, $\delta_{rmr}/\delta_{b}$
$\delta_{pl}$	μm	Downward rigid-platen displacement relative to the fixed normal bottom reference in the boundary-condition comparison
$w_{j}\left(r,t\right)$	μm	Local FT surface displacement for summit *j*, measured from its undeformed surface
$w_{mem}$	μm	Displacement contributed by retardation modes; equal to full local surface displacement after contact pressure vanishes
$\bar{G}\left(s\right)$	MPa	Operational shear modulus, $s{\tilde{G}}_{R}\left(s\right)$, in the Laplace-domain stack operator
$h_{s}$	mm	Computational substrate depth of the local stack

## Appendix A. FEM Configurations and Mesh-Convergence Assessment

For each texture class, six configurations are evaluated at five nominal pressures on two meshes: flat uncoated, flat coated instantaneous, flat coated relaxed, textured uncoated, textured coated instantaneous, and textured coated relaxed. A coarse mesh supplements the 6×5×2 reference/fine evaluations. Table A1 lists $R_{X}=100|X_{ref}-X_{fine}\left|/\right|X_{fine}|$ for $X=({\bar{u}}_{cell},{\bar{c}}_{l},{\hat{p}}_{95})$, denoted $R_{u}$, $R_{c}$, and $R_{p95}$. Here, ${\bar{c}}_{l}$ is the area-weighted top-to-interface coating compression over active contact, distinct from the coated-minus-bare cell increment $\delta_{l}$; ${\hat{p}}_{95}=p_{95}/q_{ref}$ uses the area-weighted active-contact percentile and mean active-contact pressure on each mesh.

At the additional class A FEM sampling pressures of 1.40 and 1.66MPa, the fine-mesh texture factors vary slightly and nonmonotonically. Across the reported configurations, the largest reference-to-fine changes in mean cell approach are approximately 0.217% for class A and 0.050% for class B. The largest tabulated differences in mean local coating compression are approximately 0.875% and 0.150%; these averages do not characterize convergence of the full spatial coating-deformation field (Section S11.1). The normalized pressure-percentile differences are assessed separately against the stated 3% mesh-comparison criterion. Figure 4 marks the cases exceeding that criterion.

## Appendix B. Three-Dimensional Check of the Helical-Groove Section

At $q_{nom}=1\;\mathrm{MPa}$ in the instantaneous state and on the reference discretization, the plane-section and stripe cells used the same contact-penalty setting, periodic lateral conditions, fixed normal displacement at the substrate bottom, and a rigid frictionless platen. The plane section enforced $u_{x}=0$, while the stripe cell resolved the stripe direction. The area-averaged normal approaches are compared through

$$\left|\frac{{\bar{u}}_{cell,3D}-{\bar{u}}_{cell,2D}}{{\bar{u}}_{cell,2D}}\right|<0.01\%.$$

The deviation is below 0.01%, supporting the plane-section representation for the evaluated geometry.

**Table A1:** Reference-to-fine differences for the coated textured cell pairs. The last column lists $R_{u}$, $R_{c}$, and $R_{p95}$, defined above, for mean cell approach, mean local coating compression, and normalized pressure percentile, respectively.

Class	State	$q_{\mathrm{nom}}$ (MPa)	${\bar{u}}_{\mathrm{cell},\mathrm{ref}}$ (μm)	${\bar{u}}_{\mathrm{cell},\mathrm{fine}}$ (μm)	Mesh Differences $R_{u}$/$R_{c}$/$R_{p95}$ (%)
A	Inst.	0.1	0.003541	0.003533	0.208/0.875/1.863
A	Rel.	0.1	0.003578	0.003571	0.208/0.855/0.072
A	Inst.	0.5	0.017703	0.017665	0.213/0.875/1.865
A	Rel.	0.5	0.017890	0.017852	0.213/0.855/0.070
A	Inst.	1.0	0.03541	0.035329	0.217/0.875/1.867
A	Rel.	1.0	0.035779	0.035702	0.217/0.855/0.069
A	Inst.	1.39748	0.049478	0.049437	0.082/0.436/2.141
A	Rel.	1.39748	0.050001	0.049963	0.075/0.401/0.025
A	Inst.	1.65951	0.058755	0.058693	0.105/0.505/2.099
A	Rel.	1.65951	0.059376	0.059317	0.099/0.472/0.009
B	Inst.	0.1	0.003326	0.003325	0.038/0.150/0.254
B	Rel.	0.1	0.003353	0.003352	0.039/0.148/0.259
B	Inst.	0.5	0.016630	0.016623	0.043/0.150/0.255
B	Rel.	0.5	0.016765	0.016757	0.043/0.148/0.260
B	Inst.	1.0	0.033260	0.033245	0.046/0.150/0.255
B	Rel.	1.0	0.033529	0.033514	0.046/0.148/0.261
B	Inst.	1.72400	0.057340	0.057312	0.048/0.150/0.256
B	Rel.	1.72400	0.057804	0.057776	0.049/0.148/0.261
B	Inst.	2.04725	0.068091	0.068058	0.049/0.150/0.256
B	Rel.	2.04725	0.068642	0.068608	0.050/0.148/0.261

## Appendix C. Independent Verification Summaries

### Appendix C.1. Navier/Fourier Bonded-Layer Benchmark

The Navier/Fourier solution evaluates bonded-layer compliance independently of the finite-element cells. For the instantaneous and relaxed coating states, the calculated compliance ratios differ from the reference values by less than 0.05%. Supplementary Table S4 summarizes this comparison for the prescribed lateral constraints. The calculation concerns the elastic bonded-layer response.

Supplementary Section S11.1 and Table S31 bring together this prescribed-traction comparison, the homogeneous-layer modal benchmark, and the checks of the elastic contact response, periodic-cell discretization, Fourier cutoff, local hereditary contact, and finite-ramp convolution. The separate fixed-aperture calculation examines pressure compatibility on smooth lands.

### Appendix C.2. Elastic Single-Asperity Literature Cross-Check

The frictionless single-coating spherical-contact cases of Yu et al. (2014) [30] test the elastic layered single-asperity kernel. Across $E_{l}/E_{s}=$ 0.25–4, normalized contact radius and peak pressure agree with graphical digitizations of the published curves within 1.5%, and the pressure-profile ordering is preserved. Figure A1 compares the calculated profiles with those digitizations. Because the published cases use a substrate half-space, the comparison is confined to local contact quantities. The modulus-ratio range also lies above the approximately 0.05 contrast of the present reference stack.
