# Supplementary material for: Contact-Time and Relaxation Effects on the Normal Response of Polymer-Coated Internal Cylindrical Surfaces with Deterministic Microtexture: A Layered Viscoelastic Contact Model
Source: Polymers (Basel). 2026 Oct 24;18(21):2638. doi: 10.3390/polym18212638 (PMC13766197; doi:10.3390/polym18212638)
Supplement: Supplementary file 1 [file polymers-18-02638-s001.zip › polymers-4623753-supplementary.pdf]

Article

Supplementary Materials: Contact-Time and Relaxation Effects on the Normal Response of Polymer-Coated Internal Cylindrical Surfaces with Deterministic Microtexture: A Layered Viscoelastic Contact Model

Kirill A. Bashmur [1,*], Alexander V. Zagulyaev [1], Ivan S. Nekrasov [1], Elena V. Tsygankova [2] and Tatiana N. Sviridova [2]

[1] Department of Technological Machines and Equipment of Oil and Gas Complex, Siberian Federal University, 660041 Krasnoyarsk, Russia
[2] Department of Foreign Languages for Natural Sciences, Siberian Federal University, 660041 Krasnoyarsk, Russia
* Correspondence: bashmur@bk.ru

Supplementary Materials

These supplementary materials present the model inputs, derivations, numerical checks and sensitivity analyses. Sections S1–S8 develop the component models and their numerical assessment. Section S9 illustrates mechanical components in series and a prescribed profile–pressure mapping. Section S10 examines local hereditary histories, macroscopic boundary conditions and coupling, followed in Section S11 by numerical verification and comparisons with published experiments. The Supplementary Archive provides the associated data and scripts for analytical checks and tabulated-data summaries.

Displacement references and additive decomposition

Displacement references. The local finite-thickness (FT) interference s is measured from the unloaded remote mean plane of the coated land. The statistical land-roughness approach $\delta_{plateau}$ is referenced to the upper retained-summit level z_{max}. The deterministic Fourier contribution δ_{rmr} is obtained from the nonzero, zero-cell-mean aperture-mask modes; the zero mode is excluded from this term and determines F_{union} and A_q. The increment δ_l is the cell-averaged displacement difference between corresponding coated and bare periodic cells under the same nominal load and boundary conditions, including the coating-induced change in substrate displacement. These quantities use distinct displacement-reference conventions and reference subtractions; they should not be interpreted as displacements measured from a single common reference plane.

Decomposition and scope. The adopted additive approximation is

$$\delta_{tot} = \delta_{plateau} + \delta_{rmr} + \delta_l = \delta_b + \delta_l, \qquad \delta_b = \delta_{plateau} + \delta_{rmr}.$$

At elastic endpoint $j \in \{0, \infty\}$, the texture factor ψ_j normalizes the coated-minus-bare increment by the corresponding oedometric reference,

$$\delta_{l,j} = \psi_j \frac{t_l q_{land}}{M_j}, \qquad M_j = K + \frac{4}{3} G_j.$$

For class B, $\delta_{rmr} = 0$ in the adopted decomposition. Section S6.1 examines the FT kernel and sensitivity to the Fourier contribution, while Section S10.3 addresses pressure redistribution

and coupling. The additive sum supports component-level comparisons and is not claimed to be the exact solution of a single fully coupled contact problem. Such a reference solution would enforce equilibrium and normal-displacement compatibility through a common contact-pressure field under matched geometry, constitutive history, boundary conditions and displacement references.

S1. Rough-Contact Base and Endpoint Results

S1.1. Rough-Contact Base

For one deformable coating/substrate stack against a rigid, smooth, frictionless counterface, the reduced modulus used throughout the rough-contact contribution is

$$E^* = \frac{E}{1-\nu^2}. \tag{S1}$$

The half-space branch combines Greenwood–Williamson statistical summation [1] with the Hertz single-asperity law. The reference case prescribes $R_a = 0.28$ µm and $R_{sm} = 0.50$ mm for the land roughness. R_{sm} denotes profile-element spacing in the statistical closure; the deterministic texture has separately specified periods. Let z denote summit height relative to the mean-height reference plane. The zero-mean Gaussian height model gives $\sigma = R_a\sqrt{\pi/2} = 0.351$ µm and the summit-height density

$$f_z(z) = \frac{1}{\sigma\sqrt{2\pi}} \exp\left(-\frac{z^2}{2\sigma^2}\right). \tag{S2}$$

The retained summit-height range is $z_{min} = -8\sigma \le z \le z_{max} = 3\sigma$. Force and area integrals use the Gaussian density on this range without renormalization. The isotropic, independent-asperity model uses the following prescribed statistical closure relations for the mean summit-curvature radius R_s and areal summit density η_s:

$$R_s = \frac{R_{sm}^2}{4\pi^2\sqrt{2}\,\sigma}, \qquad \eta_s = \frac{1}{R_{sm}^2}. \tag{S3}$$

The factor $\sqrt{2}$ follows from the RMS-to-peak convention $A = \sqrt{2}\sigma$ for the adopted harmonic proxy. One equivalent summit is assigned to each $R_{sm} \times R_{sm}$ statistical cell. The stated closure gives $R_s = 12.76$ mm and $\eta_s = 4.0\,\mathrm{mm}^{-2}$. Here R_s is the mean summit-curvature radius, distinct from the load-dependent contact radius. The counterface separation from the mean-height reference plane is denoted by d. At local land pressure q_{land}, d is obtained by inverting

$$q_{land} = \frac{4}{3}E^*\sqrt{R_s}\,\eta_s \int_{\max(d,z_{min})}^{z_{max}} (z-d)^{3/2} f_z(z)\,\mathrm{d}z, \qquad \delta_{plateau} = 10^3(z_{max} - d), \tag{S4}$$

In the elastic half-space (HS) and pressure-basis FT calculations, lengths inside the integral are in millimeters and $\delta_{plateau}$ is reported in micrometers. Summit-height integration uses an 80-point Gauss–Legendre rule with lower limit $\max(d, z_{min})$. A bracketed scalar root search inverts the monotone load–separation relation. The FT single-asperity load is obtained from the tabulated layered response. The hereditary discretization is specified separately in Section S10.1.

The statistical land-roughness approach is measured from the upper summit reference: in a common length unit, $\delta_{plateau} = z_{max} - d$. Force and area integrals use the Gaussian

density between $\max(d, z_{min})$ and z_{max}, without renormalization. The contacting-summit fraction is normalized by the retained population:

$$f_e(d) = \frac{\int_{\max(d,z_{min})}^{z_{max}} f_z(z)\,\mathrm{d}z}{\int_{z_{min}}^{z_{max}} f_z(z)\,\mathrm{d}z}, \qquad d < z_{max},$$

with $f_e = 0$ for $d \geq z_{max}$. The HS and FT branches share the same $R_a, R_{sm}, \sigma, R_s, \eta_s$ and summit-height convention.

For class A, $\mathcal{M}(x, y)$ is the fixed periodic binary aperture mask on the 1 mm × 1 mm cell, with $F_{union} = 0.2037$ and land fraction $\phi = 1 - F_{union}$. Its area is $A_{cell} = p_x p_y$, where p_x and p_y are the axial and circumferential periods, respectively. For integer Fourier indices m, n, define $k_x = 2\pi m/p_x$ and $k_y = 2\pi n/p_y$. Let $\widehat{A}_{mn}$ be the cell-normalized Fourier coefficient and

$$k_{mn} = \sqrt{\left(\frac{2\pi m}{p_x}\right)^2 + \left(\frac{2\pi n}{p_y}\right)^2}. \tag{S5}$$

The deterministic class A aperture is represented by the cell-normalized coefficients

$$\widehat{A}_{mn} = A_{cell}^{-1} \int_{A_{cell}} \mathcal{M}(x, y) e^{-i(k_x x + k_y y)}\, dA, \qquad \Lambda_N = \frac{2}{(1 - F_{union})^2} \sum_{\substack{|m|,|n| \leq N \\ (m,n) \neq (0,0)}} \frac{|\widehat{A}_{mn}|^2}{k_{mn}}. \tag{S6}$$

The signed square cutoff is $N = 6$; $\Lambda \equiv \Lambda_6 = 0.06036$ mm for the reference geometry. The factor 2 belongs to the one-sided half-space compliance; both signs of each wavevector are already included in the sum. Using nominal pressure, the displacement is $\delta_{rmr} = 1000 q_{nom} \Lambda_N / E^*$ in micrometers for mm and MPa inputs. The zero mode determines the aperture area and land-pressure amplification, while the nonzero modes determine the Fourier displacement increment. Table S1 checks numerical stability under larger square cutoffs using the same geometry and normalization. The $N = 6$ value is 0.6756% below the $N = 192$ value, and the difference between $N = 96$ and 192 is 0.002205% of the latter. All reported contact results retain $N = 6$. The comparison quantifies stability over the sampled finite cutoffs.

For the ellipse, $\widehat{A}_{mn} = F_{union}\, 2J_1(\rho_{mn})/\rho_{mn}$ up to a centre phase, with $\rho_{mn} = [(a_{ax}k_x)^2 + (a_{circ}k_y)^2]^{1/2}$. Here J_1 is the Bessel function of the first kind of order one. The axial and circumferential semi-axes are $a_{ax} = 0.2466$ mm and $a_{circ} = 0.2629$ mm, respectively, and both periods are 1 mm. The 168 nonzero coefficients at $N = 6$ agree with the analytical expression to the precision of the numerical data.

Table S1. Fourier compliance length for the prescribed ellipse. Differences use $100(\Lambda_N/\Lambda_{192} - 1)$.

N	Nonzero modes	Λ_N (mm)	Difference (%)
6	168	0.060355363	-0.675629
12	624	0.060659104	-0.175774
24	2400	0.060738753	-0.044700
48	9408	0.060759283	-0.010914
96	37248	0.060764575	-0.002205
192	148224	0.060765915	0.000000

S1.2. Endpoint and Pressure-Dependent Values

Table S2 gives four algebraic combinations of base and coating endpoint states. The common instantaneous and common relaxed combinations are the material endpoints; the two mixed combinations isolate the component contributions. Table S3 reports pressure-

dependent HS results. Both 5 MPa rows for class A exceed their state-specific HS closure limits and represent formal continuations outside the open-texture domain. Beyond these limits, the continued Gaussian constitutive relation describes a formal extrapolation of the open-contact branch.

Table S2. Four algebraic combinations of the HS base and coated-minus-bare increment endpoint states for class A at q_{nom} = 1 MPa.

Base state	Coated-minus-bare increment state	δ_{tot} (μm)
Inst.	Inst.	1.6261
Inst.	Rel.	1.6265
Rel.	Inst.	1.6783
Rel.	Rel.	1.6787

Table S3 gives the pressure-dependent approaches and secant and tangent stiffnesses used in Section 3.5 of the main text. Tangent stiffness is evaluated separately for each physical branch with $q_{\pm} = q(1 \pm 0.005)$ and $K_{\mathrm{t}} = (q_{+} - q_{-})/[\delta(q_{+}) - \delta(q_{-})]$. The pressure-response samples in Table S3 and the FT comparison points in Table S16 are distinct from the class-specific FEM sampling pressures in Table A1 of the main text.

Table S3. Class A HS approach and stiffness at the nominal-pressure samples. † denotes a formal continuation beyond the closure pressure of the indicated branch and endpoint state.

q_{nom} (MPa)	State	δ_b (μm)	δ_l (μm)	δ_{tot} (μm)	K_s (MPa/μm)	K_t (MPa/μm)
0.1	Inst.	0.9163	0.000945	0.9173	0.109	0.479
0.2	Inst.	1.074	0.00189	1.076	0.186	0.796
0.5	Inst.	1.34	0.00473	1.345	0.372	1.45
1	Inst.	1.617	0.00945	1.626	0.615	2.09
2	Inst.	2.013	0.0189	2.032	0.984	2.8
5†	Inst.	2.882	0.0473	2.93	1.71	3.81
0.1	Rel.	0.9391	0.000983	0.9401	0.106	0.466
0.2	Rel.	1.101	0.00197	1.103	0.181	0.771
0.5	Rel.	1.378	0.00491	1.383	0.362	1.38
1	Rel.	1.669	0.00983	1.679	0.596	1.98
2	Rel.	2.091	0.0197	2.11	0.948	2.62
5†	Rel.	3.024	0.0491	3.073	1.63	3.55

S1.3. Independent Fourier-Series Benchmark for a Bonded Layer

The analytical benchmark uses a separate 0.50 × 0.50 mm periodic cell with a centered area-equivalent circular opening of area fraction 0.392. The displacement field is obtained from a Fourier-series solution of the Navier equations. A homogeneous 50 μm layer spans the periodic cell and is perfectly bonded to a rigid support. The opening denotes an unloaded region of the upper surface, rather than a through-hole in the layer. The lateral boundaries are periodic, the lower surface is fully fixed, the upper surface is shear free, and a uniform normal traction is applied only to the land. The periods of this benchmark

cell are L_x and L_y, analogous to p_x and p_y for the class A geometry. For $k_x = 2\pi m/L_x$, $k_y = 2\pi n/L_y$, and $k = (k_x^2 + k_y^2)^{1/2}$, the pressure coefficient is

$$\widehat{p}_{mn} = \frac{1}{L_x L_y} \int_{A_{cell}} p(x,y) e^{-i(k_x x + k_y y)} \, \mathrm{d}A, \qquad A_{cell} = L_x L_y,$$

where the physical pressure $p(x,y)$ is positive in compression. For each nonzero mode, $x_\parallel$ is the local in-plane coordinate along its wavevector and z increases from the bonded lower surface to the upper surface. With the phase convention

$$u_\parallel = -iUe^{ikx_\parallel}, \qquad u_z = We^{ikx_\parallel}, \qquad \sigma_{\parallel z} = -iTe^{ikx_\parallel}, \qquad \sigma_{zz} = Se^{ikx_\parallel},$$

the phase-transformed state obeys

$$\mathbf{y}_{mn} = [U,\, W,\, T,\, S]^{\mathsf{T}}, \qquad \frac{\mathrm{d}\mathbf{y}_{mn}}{\mathrm{d}z} = \mathbf{A}(k)\mathbf{y}_{mn}, \tag{S7}$$

where, with Lamé constants λ_L and μ and $D_L = \lambda_L + 2\mu$,

$$\mathbf{A}(k) = \begin{bmatrix} 0 & k & 1/\mu & 0 \\ -\lambda_L k/D_L & 0 & 0 & 1/D_L \\ 4\mu(\lambda_L + \mu)k^2/D_L & 0 & 0 & \lambda_L k/D_L \\ 0 & 0 & -k & 0 \end{bmatrix}. \tag{S8}$$

The modal propagator is $\mathbf{T}(k) = \exp[\mathbf{A}(k)t_l]$. The amplitude boundary conditions are $U = W = 0$ at the bonded lower surface and $T = 0$, $S = -\widehat{p}_{mn}$ at the loaded upper surface. The zero mode uses the uniform bonded-layer compliance t_l/M, where the constrained modulus is $M = E(1-\nu)/[(1+\nu)(1-2\nu)]$.

With the in-plane origin at the opening centre, an elliptical opening with semi-axes a and b has the nonzero opening-indicator coefficient

$$\widehat{\chi}^{o}_{mn} = \frac{2\pi ab}{L_x L_y} \frac{J_1(\rho_{mn})}{\rho_{mn}}, \qquad \rho_{mn} = \left[(ak_x)^2 + (bk_y)^2\right]^{1/2}, \tag{S9}$$

with $\widehat{\chi}^{o}_{00} = \pi ab/(L_x L_y) = 0.392$. For the circular opening used in this benchmark, $a = b = \sqrt{0.392 L_x L_y/\pi}$. Thus $\widehat{\chi}^{l}_{00} = \phi$ and $\widehat{\chi}^{l}_{mn} = -\widehat{\chi}^{o}_{mn}$ for the nonzero land-indicator modes, where $\phi = 1 - 0.392$. For uniform pressure on the land and zero pressure on the opening, $p = (q_{nom}/\phi)\chi^l$, so

$$\widehat{p}_{mn} = \frac{q_{nom}}{\phi} \widehat{\chi}^{l}_{mn}, \qquad \widehat{p}_{00} = q_{nom}.$$

If $C_{zz}(k) = -W(t_l)/\widehat{p}_{mn}$ denotes the modal normal compliance, the square-cutoff approximation is

$$C^{(N)}_{bonded} = \frac{1}{\phi^2} \sum_{|m|,|n| \le N} C_{zz}(k_{mn}) \left| \widehat{\chi}^{l}_{mn} \right|^2. \tag{S10}$$

The reported C_{bonded} is therefore the land-averaged normal approach per unit nominal pressure. The comparison value and normalized ratio are

$$C_{1D} = \frac{t_l}{\phi\,[E/(1-\nu^2)]}, \qquad r_N = \frac{C_{bonded}}{C_{1D}}. \tag{S11}$$

Table S4 compares the independently calculated compliances and ratios with the reference values for the stated geometry and material inputs.

Table S4. Independent Fourier-series calculation for a bonded layer. Compliance is reported per unit nominal pressure; the final square cutoff is $|m|, |n| \leq 128$.

State	C_{bonded} (μm/MPa)	C_{1D} (μm/MPa)	Calculated r_N	Reference r_N	Difference (%)
Instantaneous	0.0122608	0.0186634	0.656942	0.657251	−0.0470
Relaxed	0.0128813	0.0207960	0.619410	0.619186	+0.0362

The ratio changes by 0.0017% and 0.0018% from cutoff 96 to 128 in the instantaneous and relaxed states, respectively. The full cutoff convergence data and computational inputs are provided in the supplementary data archive.

S1.4. Class B Three-Dimensional Check

The class B check used $q_{nom} = 1\,\text{MPa}$, the instantaneous coating state, the medium-mesh discretization, and the same contact-penalty setting in both cells. Both cells use periodic lateral boundary conditions, zero normal displacement at the substrate bottom, and a rigid frictionless platen. The plane-section representation additionally imposes $u_x = 0$, while the stripe cell resolves displacement in the stripe direction. The response metric was the area-averaged normal approach $\bar{u}_{cell}$ of the periodic FEM cell. Table S5 compares the plane-section and three-dimensional stripe representations under the same loading and boundary conditions.

Table S5. Class B plane-section and three-dimensional stripe comparison.

Quantity	Value	Definition
$\bar{u}_{cell,2D}$	0.03325999 μm	Plane section, $u_x = 0$
$\bar{u}_{cell,3D}$	0.03326227 μm	Three-dimensional stripe cell
Signed relative difference	+0.00686%	$100(\bar{u}_{cell,3D} - \bar{u}_{cell,2D})/\bar{u}_{cell,2D}$
Absolute relative error	0.00686% < 0.01%	$100\lvert\bar{u}_{cell,3D} - \bar{u}_{cell,2D}\rvert/\bar{u}_{cell,2D}$

The matched calculations give a signed relative difference of +0.00686% and an absolute relative error of 0.00686% < 0.01%. The observable is the area-averaged normal approach of the coated periodic cell. This comparison supports the plane-section representation under the stated conditions.

The matched constraints in this class B comparison complement the prescribed-traction layer benchmark in Section S1.3. Their influence on the macroscopic cell response is examined in Section S10.2.

S1.5. Model Inputs: Sources, Derivations, and Assumptions

Tables S6–S8 organize the material and roughness inputs, reference geometry and calculated outputs, distinguishing literature values, prescribed assumptions and derived quantities. The material idealization uses a relaxation time of 250 μs together with prescribed roughness descriptors, while the aperture geometry determines F_{union}, A_q and Λ_N through the definitions above. These inputs also define the common azimuthal pressure, $q_0 = 0.8 q^{*,HS}_{A,\infty} = 2.991$ MPa, relative to the relaxed class A HS closure pressure.

Table S6. Sources, derivations and assumptions for material and rough-contact inputs.

Quantity	Status	Value / rule	Basis and interpretation
One-term SLS model form	Prescribed reference input	Shear-relaxation law in Section 2.3 of the main text	Prescribed one-term shear-relaxation law with constant bulk modulus K
K, G_0, G_∞, τ	Prescribed reference input	5000, 1380, 1210 MPa; 250 μs	Literature epoxy-matrix parameter set reported by deBotton and Tevet-Deree, adopted as a reference idealization.
E_0, ν_0, E_∞, ν_∞, ΔG	Derived	Isotropic conversions; $\Delta G = 170$ MPa	Direct algebra from the four reference material parameters
t_l, E_s, ν_s, h_s	Prescribed reference input	50μm; 72 GPa; 0.33; 2.70 mm	Prescribed bonded-coating geometry and idealized isotropic substrate
R_a, R_{sm}	Prescribed reference input	0.28μm; 0.50 mm	Prescribed statistical descriptors of the reference land roughness
σ	Derived	$R_a\sqrt{\pi/2} = 0.350928\ \mu$m	Requires zero-mean Gaussian surface heights
R_s, η_s	Derived under the prescribed statistical closure	the stated summit closure	Mean summit-curvature radius and areal summit density calculated from the prescribed roughness descriptors using the stated closure relations

Table S7 records the prescribed geometry and Fourier convention.

Table S7. Sources, derivations and assumptions for geometry and mechanical scenarios.

Quantity	Status	Value / rule	Basis and interpretation
Compliant-crown architecture	Source-supported	Patent cited in the main text	Supports the general process architecture; residual geometry is a prescribed model input
h_{res}, ellipse semi-axes, pitch, topology	Prescribed reference input	Prescribed class A/class B reference geometry	Prescribed dimensions and topology define the reference texture geometry
F_{union}, ϕ, A_q	Derived	$F_{union} = A_{union}/A_{cell}$; $\phi = 1 - F_{union}$; $A_q = 1/\phi$	The prescribed periodic ellipse/cell geometry gives $F_{union} = 0.2037$ for class A
Contact pair and finite-thickness comparison pressures	Prescribed reference input	One coated stack/rigid counterface; 0.1, 1.0, 1.852 MPa	Contact-pair idealization and finite-thickness comparison points
Crown/spring component inputs	Prescribed reference input	98.304 and 100.0 N/μm reference components	Prescribed reference mechanical inputs
Added mechanical interference δ_0	Prescribed reference input	20μm	Defines the reference mechanical state for the contact-zone/crown/spring series combination and kinematic profile-range screening; distinct from the residual texture depth h_{res}
Azimuthal amplitudes/modes and inverse threshold	Prescribed reference input	20%, 10%, 30% peak-to-peak nominal-pressure ranges; 5.0μm threshold	Illustrative prescribed hub/cam, tooth, and ovality patterns with a chosen profile-range screening threshold
Fourier-term multipliers	Prescribed reference input	$0\times, 1\times, 2\times\delta_{rmr}$	Prescribed multipliers for assessing sensitivity to possible roughness–texture overlap
Class A Λ	Derived	0.06036 mm	Full signed N=6 ellipse Fourier lattice; one-sided Green factor 2 and land-mean normalization.
Common angular reference pressure	Prescribed margin	$q_0 = 0.8q^*_{A,\infty,\mathrm{HS}} = 2.991$ MPa	Prescribed common sub-closure pressure for branch comparisons.

Table S8 identifies the definitions and derivations of the reported outputs.

Table S8. Definitions and sources of derived computational outputs.

Quantity	Status	Value / rule	Basis and interpretation
Navier/Fourier compliances and ratios	Derived	Fourier-series solution with the specified inputs and convergence assessment	Forward solution is independent of the reference ratios; equations, BCs, normalization, and ratio are stated in Section S1.3
Class B section/stripe check	Derived	Underlying 2D/3D outputs and $100(\bar{u}_{\text{cell,3D}} - \bar{u}_{\text{cell,2D}})/\bar{u}_{\text{cell,2D}}$	$q_{nom} = 1$ MPa, instantaneous state, medium-mesh discretization, matched penalty and documented BCs
K_{eq} measures	Derived	Force–approach components used in the calculation	Incremental secant, tangent, and total-force/interference definitions remain distinct
Azimuthal stiffness spreads and kinematic profile range	Derived	Paired half-space and finite-thickness pressure-to-stiffness responses; kinematic mapping $e(\theta) = \delta_0 m(\theta)$.	The same prescribed pressure fields are used in both formulations. A ±10% pressure modulation corresponds to a 4.0 μm profile range, compared with a prescribed 5.0 μm screening threshold.
Finite-thickness results	Derived	Layered single-asperity integration with the prescribed summit distribution	Approach, secant stiffness, and tangent stiffness defined in Section S6.1
Monte Carlo and Morris outputs	Derived	Output percentiles under the prescribed input distributions	Normalized elementary effects over the specified parameter bounds
δ_b	Defined	$\delta_{\text{plateau}} + \delta_{\text{rmr}}$	Base term combines the statistical land-roughness approach and deterministic Fourier displacement increment.
χ_{rmr}	Derived	$\delta_{\text{rmr}}/\delta_b$	Fourier fraction of the base approach.
Φ_l	Derived	$\delta_l/\delta_{\text{tot}}$	Coated-minus-bare fraction of total approach; its complement is the base fraction.

S2. Characteristic Times and Transition Measures

For class A, $a_{track} = 254.6\,\mu$m and $t_c = 2a_{track}/v_{track} = 0.5093$ ms at the prescribed speed $v_{track} = 1$ m/s. The material relaxation time is $\tau = 0.250$ ms, or 250 μs. The retardation times are $\tau_{ret,b} = \tau G_0/G_\infty = 0.2851$ ms and $\tau_{c,l} = \tau M_0/M_\infty \simeq 0.25857$ ms, giving $\Theta_b = 1.786$ and $\Theta_l \simeq 1.970$. Here $a_{track} = \sqrt{a_{ax}a_{circ}}$ is the radius of a circle with the area of the reference elliptical footprint. At fixed temperature and material properties, speed rescales the prescribed local loading duration and these time ratios. The local histories remain fixed in position; a translating footprint requires a spatially and temporally resolved loading history.

$$t_c = \frac{2a_{track}}{v_{track}} = \frac{2(254.6\,\mu\text{m})}{1.000 \times 10^6\,\mu\text{m/s}} = 0.509\,\text{ms}. \tag{S12}$$

The shear relaxation modulus is

$$G_R(t) = G_\infty + (G_0 - G_\infty)\exp\left(-\frac{t}{\tau}\right). \tag{S13}$$

For a held shear-stress step, the corresponding creep compliance, retardation time, and effective creep-derived modulus are

$$J_G(t) = \frac{1}{G_\infty} - \left(\frac{1}{G_\infty} - \frac{1}{G_0}\right)\exp\left(-\frac{t}{\tau_{ret,b}}\right), \qquad \tau_{ret,b} = \tau\frac{G_0}{G_\infty}, \qquad \widehat{G}_b(t) = \frac{1}{J_G(t)}. \tag{S14}$$

The HS time curve inserts the effective creep modulus $\widehat{G}_b(t) = 1/J_G(t)$ into the static branch, giving a quasi-elastic approximation. Its base half-transition and e-folding times are approximately 0.194 and 0.280 ms. The adopted scalar coated-minus-bare law has $P_l(t) = 1 - e^{-t/\tau_{c,l}}$, with $t_{50,l} = \tau_{c,l} \ln 2 \simeq 0.179$ ms. At the reference loading duration, the HS quasi-elastic base approach and coated-minus-bare increment reach approximately 83.6% and 86.0% of their respective endpoint changes.

For a held land-pressure amplitude q_h, the scalar compliance below specifies the adopted time dependence of the coated-minus-bare periodic-cell increment. The endpoint factors ψ_j include both coating deformation and the associated change in substrate displacement under matched loading and boundary conditions:

$$C_l(t) = C_{l,\infty} + (C_{l,0} - C_{l,\infty}) \exp\left(-\frac{t}{\tau_{c,l}}\right), \qquad C_{l,0} = \frac{t_l \psi_0}{M_0}, \qquad C_{l,\infty} = \frac{t_l \psi_\infty}{M_\infty}. \tag{S15}$$

For the stated constant-K material law, the exact homogeneous constrained-strain creep compliance is

$$J_M(t) = \frac{1}{M_\infty} + \left(\frac{1}{M_0} - \frac{1}{M_\infty}\right) e^{-t/\tau_{c,l}}, \qquad \tau_{c,l} = \tau \frac{M_0}{M_\infty}, \qquad M_j = K + \frac{4}{3} G_j.$$

A flat layer under uniform constrained compression therefore has compliance $t_l J_M(t)$. Equation (S15) retains this retardation time and interpolates between the full cell endpoints $t_l \psi_j / M_j$. Assigning this single mode to the heterogeneous cell is a modeling approximation. Its full transient redistribution requires the corresponding periodic boundary-value solution. The endpoint coefficients ψ_j characterize the elastic cell states. Writing $C_l(t) = t_l \psi_{eff}(t) J_M(t)$ defines ψ_{eff} as an algebraic ratio of the adopted compliance to the homogeneous-layer response. For constant $\psi_0 = \psi_\infty = \psi$, the adopted law reduces to $\psi t_l J_M(t)$.

The held amplitude is $q_h = q_{land} = q_{nom}/(1 - F_{union})$. At $q_{nom} = 1\,\mathrm{MPa}$ for class A, $q_h = 1.256\,\mathrm{MPa}$, $M_0 = 6840\,\mathrm{MPa}$, $M_\infty = 6613\,\mathrm{MPa}$ and $\tau_{c,l} = 0.2586\,\mathrm{ms}$. The cell-increment endpoints are 0.009452 μm and 0.009826 μm. At the prescribed duration $t_c = 0.5093\,\mathrm{ms}$, $1 - e^{-t_c/\tau_{c,l}} = 0.8605$. The held-step and finite-duration rectangular-pulse curves are

$$\delta_l^{step}(t) = q_h C_l(t), \tag{S16}$$

$$\delta_l^{pulse}(t) = \begin{cases} q_h C_l(t), & 0 < t \le t_c, \\ q_h [C_l(t) - C_l(t - t_c)], & t > t_c. \end{cases} \tag{S17}$$

At removal, the left and right limits are $q_h C_l(t_c)$ and $q_h [C_l(t_c) - C_l(0)]$, respectively. The data for Figure 9 of the main text include both limits. Subsequent pulse recovery is normalized by the right-limit value. The tabulated history values follow linear superposition for the adopted scalar coated-minus-bare law and verify its numerical evaluation. For held loading in the additive FT model, the local plateau approach is obtained from the hereditary contact solution. The deterministic Fourier displacement increment uses the exact constant-K reduced creep compliance,

$$J_{E^*}(t) = \frac{1}{4}[J_G(t) + 3J_{3K+G}(t)],$$

where J_{3K+G} is the creep compliance associated with the operational modulus $3K + \overline{G}(s)$. Thus $\delta_{rmr}(t) = 1000 q_{nom} \Lambda_N J_{E^*}(t)$. The coated-minus-bare increment follows the endpoint-matched scalar compliance $C_l(t)$. This additive held-load response combines separately

defined operators. The hereditary contact solution describes the local FT contribution under its prescribed loading; texture interactions are considered in Section S10.3.

For a fixed t_c, the material relaxation-time interval corresponding to 5–95% completion of an exponential factor follows from $1 - e^{-t_c/\tau_{ret}} = 0.05$ and 0.95, using the relevant retardation-to-material-time ratio. The HS base and scalar coated-minus-bare windows are approximately 0.149–8.71 ms and 0.164–9.60 ms, respectively. These fixed-duration windows describe the named exponential factors; the nonlinear GW response has its own transition measures.

Linear step superposition gives the rectangular-pulse response of the adopted scalar coated-minus-bare increment. Local FT unloading requires the hereditary unilateral-contact solution of Section S10.1, which evolves the contact plane and active asperity set while retaining the memory state. Superposition of total step responses is not valid for this nonlinear contact problem.

S3. Propagation of Prescribed Parameter Distributions

The Monte Carlo calculation propagates prescribed parameter distributions for class A at 1 MPa using 4000 samples and seed 20260730. The lognormal material relaxation time has arithmetic mean 250 μs and coefficient of variation 0.16. Resampling enforces $0 < G_\infty < G_0$. These prescribed distributions define the input-variation study. Table S9 defines the inputs.

Table S9. Monte Carlo input distributions and constraints for 4000 samples of the reference model.

Input	Distribution / specification	Constraint
τ	Lognormal; mean 250 μs; CV 0.16	Prescribed variation around the literature reference
G_0	Normal; mean 1380 MPa; SD 103.5 MPa	$G_\infty < G_0$
G_∞	Normal; mean 1210 MPa; SD 90.75 MPa	$0 < G_\infty < G_0$
K	Normal; mean 5000 MPa; SD 200 MPa	Positive modulus
t_l	Normal; mean 50.0 μm; SD 3.5 μm	Positive thickness
F_{union}; q_{nom}	Fixed 0.2037; 1.00 MPa	Area fraction calculated from the prescribed aperture geometry at full precision

For the relaxed HS branch, the 5th percentile, median and 95th percentile of total approach are approximately 1.640, 1.683 and 1.739 μm. The corresponding coated-minus-bare increment percentiles are 0.00861, 0.00985 and 0.0112 μm. The median coating share is 0.00585. The coating time-ratio percentiles are 1.51, 1.98 and 2.57, expressing the prescribed loading duration relative to the scalar cell-increment retardation time. Table S10 gives all outputs with their units and normalization.

Table S10. Relaxed class A HS output percentiles at q_{nom} = 1 MPa, with Λ = 0.0604 mm and the relaxation-time distribution centered at 250 μs.

Output	P5	Median	P95
K_s (MPa/μm)	0.575	0.594	0.610
Base approach (μm)	1.630	1.673	1.729
Coated-minus-bare increment (μm)	0.00861	0.00985	0.0112
Total approach (μm)	1.640	1.683	1.739
Φ_l (dimensionless)	0.00513	0.00585	0.00659
Θ_l (dimensionless)	1.51	1.98	2.57

S4. Morris Screening

Morris screening [2] uses four normalized levels, a step of 2/3, 14 trajectories and seed 20260731, giving 126 evaluations. Each trajectory satisfies $0 < G_\infty < G_0$, and $A_q = (1 - F_{union})^{-1}$ is evaluated for each sampled open fraction. The material-time bounds are 210–290 μs. The elementary effect of each input is normalized by its midpoint and by the response at the joint midpoint of all bounds, as defined in the accompanying equation. The summaries are the mean absolute effect μ^* and the standard deviation σ_{EE}.

Table S11 lists the input bounds.

Table S11. Input bounds for conditional Morris screening using a normalized parameter design.

Input	Lower	Upper
K (MPa)	4800	5200
G_0 (MPa)	1276.5	1483.5
G_∞ (MPa)	1119.25	1300.75
τ (μs)	210	290
t_l (μm)	46.5	53.5
q_{nom} (MPa)	0.8	1.2
F_{union}	0.1935	0.2139
h_{res} (μm)	2.567	2.837

$$EE_i = \frac{(y_1 - y_0)/y_{mid}}{(x_{i,1} - x_{i,0})/x_{i,mid}}, \tag{S18}$$

The outputs are the relaxed response, the endpoint span and the quasi-elastic response at a one-second dwell. The relaxed response and endpoint span are independent of τ. At the reference material time, the one-second dwell corresponds to $t/\tau = 4000$; its quasi-elastic response is saturated over the sampled parameter range, giving zero computed elementary effects for τ. Time dependence near the prescribed loading duration is characterized separately in Section S2. The Fourier compliance length Λ_N is held fixed while F_{union} varies. This conditional screening captures land-fraction effects without recomputing the aperture-dependent Fourier contribution. A geometry-consistent variation would also require recalculating the aperture Fourier coefficients and Λ_N. The residual depth acts as a domain filter and therefore has no response effect when all compared states remain open. Each reported input/output pair has 14 valid elementary effects. The spread σ_{EE} describes the variation of the 14 elementary effects.

Table S12 summarizes the normalized elementary effects.

Table S12. Normalized Morris elementary effects, shown as ordered pairs $(\mu^*, \sigma_{\mathrm{EE}})$, for the reference model; each entry uses 14 valid effects. A pair $(0, 0)$ denotes zero mean absolute effect and zero standard deviation across the 14 effects. The Fourier compliance length Λ_N is held fixed during this conditional screening.

Input; $(\mu^*, \sigma_{\mathrm{EE}})$	K_s, relaxed	δ_{tot} at 1 s	δ_{tot}, relaxed	Endpoint increase	Φ_l, relaxed
K	$(0.0545, 0.00747)$	$(0.0541, 0.00426)$	$(0.0541, 0.00426)$	$(0.105, 0.0516)$	$(0.697, 0.0663)$
G_0	$(0.00023, 4.05 \times 10^{-5})$	$(0.000225, 3.14 \times 10^{-5})$	$(0.000225, 3.14 \times 10^{-5})$	$(7.6, 0.31)$	$(0.0373, 0.00403)$
G_∞	$(0.25, 0.037)$	$(0.249, 0.0239)$	$(0.249, 0.0239)$	$(8.02, 0.442)$	$(0.0306, 0.0129)$

Table S12 (continued)

Input; $(\mu^*, \sigma_{\mathrm{EE}})$	K_s, relaxed	δ_{tot} at 1 s	δ_{tot}, relaxed	Endpoint increase	Φ_I, relaxed
τ	(0, 0)	(0, 0)	(0, 0)	(0, 0)	(0, 0)
t_I	(0.00565, 0.001)	(0.00573, 0.000778)	(0.00573, 0.000778)	(0.00159, 0.000764)	(0.977, 0.085)
q_{nom}	(0.696, 0.0241)	(0.302, 0.0124)	(0.302, 0.0124)	(0.284, 0.151)	(0.703, 0.0481)
F_{union}	(0.0754, 0.0105)	(0.0749, 0.00731)	(0.0749, 0.00731)	(0.0663, 0.0359)	(0.185, 0.0187)
h_{res}	(0, 0)	(0, 0)	(0, 0)	(0, 0)	(0, 0)

S5. Relaxation-Spectrum-Shape Check

The spectrum-shape comparison uses a one-term shear-relaxation law and an equal-weight two-term spectrum with times $\tau/3$ and 3τ, at fixed G_0 and G_∞. For $R_G(t) = [G_0 - G_R(t)]/(G_0 - G_\infty)$, the normalized half-transition time changes from $t_{50,G}/\tau = 0.693$ to 0.580. At $t_c = 0.5093$ ms and $\tau = 250\,\mu$s, the respective progress values are approximately 87.0% and 74.5%, a decrease of about 12.5 percentage points. This comparison characterizes shear relaxation between identical endpoints. A two-term FT contact history would require a separate hereditary contact solution.

Table S13 compares the two prescribed shear-relaxation spectra.

Table S13. Shear-relaxation spectrum-shape comparison at the reference relaxation time τ = 250 μs and t_c = 0.509 ms.

Shear relaxation spectrum	$R_G(t_c)$ (%)	$t_{50,G}$ (ms)
One term	87	0.173
Two equal terms at $\tau/3$ and 3τ	74.5	0.145

S6. Finite-Thickness Calculations and Reproducibility

S6.1. Finite-Thickness Rough Contact and Fourier-Term Sensitivity

The FT formulation uses the prescribed truncated-Gaussian summit distribution and an axisymmetric single-asperity solution for a locally planar coated substrate against a rigid, smooth, frictionless counterface. This local approximation represents the coated internal cylindrical surface and uses the one-sided contact convention defined in Section S1.1. The reference stack consists of a 50 µm coating perfectly bonded to a 2.70 mm isotropic substrate with $E_s = 72\,$GPa and $\nu_s = 0.33$. The thickness and substrate properties are prescribed parameters, perfect bonding is a model assumption, and the lower substrate boundary is fixed. For asperity interference s and contact radius a, the displacement in the contact region is

$$w(r) = s - \frac{r^2}{2R_s}, \qquad 0 \le r \le a. \tag{S19}$$

For a Fourier mode of magnitude k, choose z upward from the bottom and write $u_x = -iUe^{ikx}$, $u_z = We^{ikx}$, $\sigma_{xz} = -iTe^{ikx}$ and $\sigma_{zz} = Se^{ikx}$. The elastic state $\mathbf{y} = (U, W, T, S)^{\mathsf{T}}$ obeys

$$\frac{d\mathbf{y}}{dz} = \mathbf{A}\mathbf{y}, \quad \mathbf{A} = \begin{pmatrix} 0 & k & 1/\mu & 0 \\ -\lambda k/D & 0 & 0 & 1/D \\ 4\mu(\lambda+\mu)k^2/D & 0 & 0 & \lambda k/D \\ 0 & 0 & -k & 0 \end{pmatrix}, \qquad D = \lambda + 2\mu.$$

Here $\mu = G$ and $\lambda = K - 2G/3$ are the Lamé moduli of the relevant layer, while $\Lambda \equiv \Lambda_6$ denotes the adopted Fourier compliance length. The transfer matrix is $\mathsf{T} = e^{\mathbf{A}_l t_l} e^{\mathbf{A}_s h_s}$. With displacement/traction blocks, bottom conditions $U = W = 0$, and upper tractions $(T, S) = (0, -\widehat{p})$, the downward compliance is

$$C_{zz}(k) = \mathbf{e}_2^{\mathsf{T}} \mathsf{T}_{ut} \mathsf{T}_{tt}^{-1} \mathbf{e}_2, \qquad \mathbf{e}_2 = (0, 1)^{\mathsf{T}}.$$

Perfect bonding enforces continuity of all state components at the interface. The positive-wavenumber compliance enters the Hankel pair in the main text; its homogeneous half-space and $k \to 0$ limits are $2/(E^* k)$ and $t_l/M_l + h_s/M_s$, respectively. Here M_l and M_s are the constrained moduli of the coating and substrate, respectively.

The elastic solver uses the edge-weighted pressure expansion

$$p(r) = \sqrt{1 - (r/a)^2} \sum_{j=0}^{3} c_j P_j\Big(2(r/a)^2 - 1\Big),$$

where P_j are Legendre polynomials, with four coefficients, 700 spectral integration nodes and 120 contact-radius samples for the single-asperity relation. The composite Hankel quadrature contains 420 nodes. For each layer of nominal thickness h, the matrix exponential at $k > 0$ uses the effective propagation thickness $\min(h, 18/k)$. Here $h = h_s$ for the substrate and $h = t_l$ for the coating. This numerical truncation limits exponential growth at large kh. The analytical homogeneous-slab reference in the spectral comparison below is evaluated at the full nominal substrate depth. The GW integration uses the same prescribed population as the HS comparison.

The pressure-basis solution gives the single-asperity load $P_{layered}(s)$. Integrating this load over the Gaussian summit population gives the land pressure,

$$q_{land} = \eta_s \int_{\max(d, z_{min})}^{z_{max}} P_{layered}(z - d) f_z(z)\, \mathrm{d}z, \tag{S20}$$

which is inverted using the same separation convention as the HS branch. The local interference is measured from the unloaded remote mean plane of the coated land and uses the positive-wavenumber response of one bonded coating/substrate stack. The separate cell-averaged increment δ_l is the coated-minus-bare periodic-cell response, including the ψ edge-release correction. For the elastic endpoint calculations, the characteristic contact scale is the number-weighted mean radius of contacting summits.

The analytical limit as $t_l \to 0$ at fixed h_s is a homogeneous elastic layer of thickness h_s with the same clamped bottom and shear-free upper surface. Its normal compliance for $k > 0$ is

$$C_{zz}^{\mathrm{slab}}(k) = \frac{(\beta_s + 1)[\beta_s \sinh(2kh_s) - 2kh_s]}{2\mu_s k[\beta_s^2 + 1 + 2\beta_s \cosh(2kh_s) + 4(kh_s)^2]}, \tag{S21}$$

where $\beta_s = 3 - 4\nu_s$, $\mu_s = E_s/[2(1 + \nu_s)]$ and $E_s^* = E_s/(1 - \nu_s^2)$. This expression follows from the homogeneous elastic boundary-value problem with $U = W = 0$ at the bottom and $(T, S) = (0, -\widehat{p})$ at the top, using downward displacement $w = -W$ and the Hankel normalization stated in the main text. It has units of length per pressure and satisfies

$$C_{zz}^{\mathrm{slab}}(0) = \frac{h_s}{M_s}, \qquad C_{zz}^{\mathrm{slab}}(k) \longrightarrow \frac{2}{E_s^* k} \quad \text{as } kh_s \to \infty,$$

with $M_s = E_s(1 - \nu_s)/[(1 + \nu_s)(1 - 2\nu_s)]$. Removing the coating at fixed depth therefore gives a finite-layer contact problem; recovering Hertz contact additionally requires

$h_s/a \to \infty$. These local FT boundary conditions differ from the normal bottom constraint with a tangential anchor used for the periodic-cell coating increment.

Table S14 compares the layered spectral compliance with an independent evaluation of Equation (S21). Material properties and nominal substrate depth are fixed: $E_s = 72.00$ GPa, $\nu_s = 0.33$, $h_s = 2.70$ mm, $E_l = 3791$ MPa and $\nu_l = 0.3736$. The 244 fixed modes comprise $k = 0$, 241 logarithmically spaced points from 0.01 to 100 mm^{-1}, and two additional points near 6.67 and 7.41 mm^{-1}; the exact grid and material inputs are provided in the supplementary archive. The signed relative error is $e(k, t_l) = 100[C_{zz}^{\mathrm{FT}}(k, t_l)/C_{zz}^{\mathrm{slab}}(k) - 1]$.

The comparison requires positive finite compliance, a final grid maximum $\max_k |e| <$ 1%, at least fivefold error reduction per thickness decade at each sampled k over $t_l = 0.5, 0.05, 0.005\,\mu$m (with a 10^{-6}% roundoff floor), and sensitivity to the propagation-depth limit below 0.2% of the analytical compliance at the final thickness. All criteria are satisfied. The closed form agrees with an independent 80-digit Airy boundary solution to within 1.12×10^{-15} relative error on this grid. The final three maximum errors are 27.96%, 2.798% and 0.2798%. The propagation-depth limits of 18 and 20 give identical values at the reported precision.

The thickness sequence gives a decreasing maximum spectral-compliance error over the sampled fixed modes. At $t_l = 5\,\mu$m, the errors at $k = 0$, 1 and 10 mm^{-1} are 2.888%, 3.190% and 27.96%, respectively. The smallest thickness is a mathematical limit probe. This check evaluates the spectral kernel. Load, approach, pressure and contact-set convergence require contact calculations with their own discretization checks.

Table S14. Signed spectral-compliance errors e (%) at selected fixed modes (k in mm^{-1}), and the maximum absolute error over all 244 modes. Material properties and nominal substrate depth are fixed.

t_l (μm)	$k = 0$	$k = 1$	$k = 10$	$k = 100$	$\max_k \lvert e \rvert$
50	+28.88	+31.89	+299.1	+1727	1727
5	+2.888	+3.190	+27.96	+299.1	299.1
0.5	+0.2888	+0.3191	+2.798	+27.96	27.96
0.05	+0.02888	+0.03191	+0.2798	+2.798	2.798
0.005	+0.002888	+0.003191	+0.02798	+0.2798	0.2798

The finite-bottom comparison in Table S15 uses identical coating and substrate properties, $E = 3791$ MPa and $\nu = 0.3736$, with thicknesses 0.05 and 2.70 mm (total homogeneous depth 2.75 mm). Both layers thus have the instantaneous coating properties. The bottom is fully clamped and the top is frictionless; the summit radius is $R_s = 12.76$ mm. At the same contact radius, the Hertz reference is $P_H = 4E^* a^3/(3R_s)$ and $\delta_H = a^2/R_s$, where $E^* = E/(1 - \nu^2)$. The maximum absolute differences are 0.01681% in load and 4.075% in approach, illustrating the different depth sensitivity of these two observables. This comparison concerns the specified finite homogeneous geometry.

Table S15. Homogeneous finite-bottom/Hertz comparison at 2.75 mm total depth. Differences are $100(X_{\text{calc}}/X_H - 1)$ for load P and approach δ separately. Percentages use the unrounded values.

a (mm)	P_{calc} (N)	P_H (N)	ΔP (%)	δ_{calc} (μm)	δ_H (μm)	$\Delta\delta$ (%)
0.06	0.099454291	0.099453667	+0.0006268	0.278286	0.282134	-1.364
0.10	0.460446976	0.460433645	+0.002895	0.765905	0.783705	-2.271
0.18	2.68570045	2.68524902	+0.01681	2.43574	2.53920	-4.075

Three additional homogeneous comparisons use the same material with a 5 mm coating and 5 mm substrate. At contact radii 0.06, 0.10 and 0.18 mm, the maximum absolute differences from Hertz are 0.0003525% in load and 1.125% in approach. These comparisons characterize this fixed total depth of 10 mm.

Table S16. Class A elastic plateau approaches from HS and FT at matched nominal pressures. † marks pressure samples beyond the state-specific HS closure limit; the corresponding FT results remain within the open-texture domain.

q_{nom} (MPa)	State	$\delta^{\text{HS}}_{\text{plateau}}$ (μm)	$\delta^{\text{FT}}_{\text{plateau}}$ (μm)	FT-minus-HS (%)
0.1	Inst.	0.915	0.6878	-24.8
0.2	Inst.	1.071	0.7974	-25.6
0.5	Inst.	1.333	0.9655	-27.6
1	Inst.	1.603	1.116	-30.4
2	Inst.	1.985	1.295	-34.8
5†	Inst.	2.814	1.598	-43.2
0.1	Rel.	0.9376	0.6969	-25.7
0.2	Rel.	1.098	0.8073	-26.5
0.5	Rel.	1.37	0.9767	-28.7
1	Rel.	1.654	1.128	-31.8
2	Rel.	2.06	1.309	-36.5
5†	Rel.	2.948	1.616	-45.2

At $q_{nom} = 1$ MPa for class A, the instantaneous HS and FT total approaches are 1.626 and 1.139 µm; the relaxed values are 1.679 and 1.154 µm. The corresponding instantaneous secant stiffnesses are 0.615 and 0.878 MPa/μm, and the relaxed values are 0.596 and 0.867 MPa/μm. Branch-specific central differences give instantaneous/relaxed tangent stiffnesses of 2.09/1.98 for HS and 3.87/3.81 MPa/μm for FT. Total approach increases by 3.23% between endpoints in HS and 1.27% in FT. The ratio of base to coated-minus-bare endpoint increments, $\Delta\delta_b/\Delta\delta_l$, is 139.45 for HS and 37.70 for FT. These elastic pressure-basis results are summarized in Table 4 of the main text.

For the six refinement-test cases combining class A nominal pressures of 0.1, 1.0 and 1.852 MPa with the two elastic endpoint states, the fine discretization changes the plateau approach by at most 0.0028% relative to the pressure-basis discretization defined above, and the coarse discretization by at most 0.0186%. The coarse and fine settings use, respectively, 3 and 5 pressure-basis coefficients, 294 and 560 composite Hankel nodes, 500 and 900 spectral nodes, and 90 and 150 contact-radius samples. Their dimensionless propagation-depth limits are 16 and 20, compared with 18 for the reference discretization.

Table S16 compares plateau approaches at matched pressures; Table 4 of the main text gives the component and total endpoints. The spectral and finite-depth comparisons above complement the boundary-condition study in Section S10.2 and the single-asperity literature comparison in Section S8, each with its stated observable and reference.

Table S17 lists closure pressures for each branch and endpoint state. FT values beyond 5 MPa are formal continuations; the contact model applies while the cavity remains open. Table S18 varies the multiplier j of the deterministic Fourier term, $\delta_{tot}^{(j)} = \delta_{plateau}^{FT} + j\delta_{rmr} + \delta_{l}$, with $j = 0, 1, 2$. The normalized range is $W = 100[\delta_{tot}^{(2)} - \delta_{tot}^{(0)}]/\delta_{tot}^{(1)}$. This range quantifies conditional component sensitivity; Section S10.3 addresses the corresponding pressure interactions.

Table S17. Branch- and state-specific closure pressures and their status relative to the prescribed pressure range.

Class	State	Branch	Closure (MPa)	Domain status
A	Inst.	HS	4.16	Within 0.1–5 MPa
A	Inst.	FT	18.3	Formal continuation above 5 MPa
A	Rel.	HS	3.74	Within 0.1–5 MPa
A	Rel.	FT	17.4	Formal continuation above 5 MPa
B	Inst.	HS	5.28	Formal continuation above 5 MPa
B	Inst.	FT	28.9	Formal continuation above 5 MPa
B	Rel.	HS	4.75	Within 0.1–5 MPa
B	Rel.	FT	27.9	Formal continuation above 5 MPa

Table S18. Prescribed Fourier-term multiplier sensitivity for relaxed classes A and B in the additive FT formulation.

Class	q_{nom} (MPa)	Fourier-term multiplier	δ_{tot} (μm)	K_s (MPa/μm)	K_t (MPa/μm)
A	0.1	0	0.6979	0.143	0.662
A	0.1	1	0.6994	0.143	0.656
A	0.1	2	0.7009	0.143	0.649
A	1	0	1.138	0.879	4.05
A	1	1	1.154	0.867	3.81
A	1	2	1.169	0.856	3.6
A	1.85	0	1.305	1.42	6.21
A	1.85	1	1.334	1.39	5.67
A	1.85	2	1.362	1.36	5.22
B	0.1	0	0.6701	0.149	0.684
B	0.1	1	0.6701	0.149	0.684
B	0.1	2	0.6701	0.149	0.684
B	1	0	1.093	0.915	4.26
B	1	1	1.093	0.915	4.26
B	1	2	1.093	0.915	4.26
B	1.85	0	1.251	1.48	6.6
B	1.85	1	1.251	1.48	6.6
B	1.85	2	1.251	1.48	6.6

Table S18 reports the sensitivity to a prescribed multiplier of the deterministic Fourier displacement increment, with the other contributions fixed. Class B is unchanged because $\delta_{rmr} = 0$ in this formulation; the base approach remains dominant in the class A cases

shown. This conditional component sensitivity complements the pressure-interaction comparisons in Section S10.3 and the fixed-aperture smooth-land calculation in Section S11.1.

Table S19 collects the reference geometries and the kinematic definition of the loading duration.

Table S19. Reference texture geometries and the kinematic definition of the loading duration.

Case	Geometry	Derived / prescribed quantities
Class A	$h_{res} = 2.702\,\mu$m; $a_{ax} = 246.627\,\mu$m; $a_{circ} = 262.905\,\mu$m; $p_x = p_y = 1000\,\mu$m	$a_{track} = \sqrt{a_{ax}a_{circ}} = 254.636\,\mu$m; $F_{union} = 0.2037$; $A_q = 1.256$
Class B	$h_{res} = 2.702\,\mu$m; circumferential pitch 13.090 mm; axial period 85 μm	$F_{union} = 0.03997$; $A_q = 1.042$
Class A loading duration	$v_{track} = 1.0\,$m/s	$t_c = 2a_{track}/v_{track} = 0.509$ ms for class A

S6.2. Software and Reproducibility

The statistical calculations used Python 3.13.5, NumPy 2.3.5 and SciPy 1.17.0. Monte Carlo propagation used 4000 samples and seed 20260730; Morris screening used four levels, 14 trajectories and seed 20260731. The Navier/Fourier and finite-thickness calculations are deterministic. The Supplementary Archive contains numerical results, verification datasets, Monte Carlo samples and Morris elementary effects, together with input and source information. Its eight reproduction programs evaluate the specified analytical quantities and tabulated-data statistics and were tested using Python 3.12.4, NumPy 2.1.3 and SciPy 1.15.1. The README and scientific index map each program to its inputs and outputs.

The elastic pressure-basis and hereditary annular-force formulations use the same layer/substrate operator. Their elastic endpoints, time histories, spectral error records and numerical comparisons are supplied with the corresponding displacement references and reference subtractions. Section S10.3 defines the pressure-interaction comparisons. Additional finite-element model files are available from the corresponding author upon reasonable request.

S7. Local Sensitivity, Regime Criteria, and Paired Azimuthal Response

S7.1. Local Topography Sensitivity

The local analysis treats R_a and R_{sm} as the primary descriptors. Each perturbed pair (R_a, R_{sm}) determines a corresponding set of σ, R_s, η_s, z_{min} and z_{max} through the same statistical closure:

$$R_a, R_{sm} \longrightarrow \quad \sigma = R_a\sqrt{\pi/2}, \qquad R_s = \frac{R_{sm}^2}{4\pi^2\sqrt{2}\,\sigma},$$
$$\eta_s = R_{sm}^{-2}, \qquad z_{min} = -8\sigma, \qquad z_{max} = 3\sigma.$$

For an output y and primary descriptor x, the logarithmic sensitivity is

$$S_x^y = \frac{\ln y[x(1+h)] - \ln y[x(1-h)]}{\ln(1+h) - \ln(1-h)}.$$

Local logarithmic sensitivities are evaluated by perturbing each primary descriptor, R_a or R_{sm}, and recomputing σ, R_s, η_s, z_{min} and z_{max} from the same statistical closure. The reference is relaxed class A in the HS branch at 1 MPa. The reported step is $h = 0.01$, with

$h = 0.005$ and 0.02 used for comparison. Across the six derivatives of δ_{tot}, K_s and K_t, the maximum relative change is approximately 0.0055%. Table S20 and Figure S1 summarize these sensitivities.

Table S20. Local logarithmic sensitivities of total approach and normal stiffnesses to prescribed roughness descriptors for relaxed class A at 1 MPa in the HS formulation. The central step is h=0.01; comparisons use h=0.005 and 0.02.

Descriptor	Output	Log sensitivity	Maximum step variation (%)
R_a	Total approach (μm)	+0.699	0.0019
R_a	K_s (MPa/μm)	-0.699	0.0019
R_a	K_t (MPa/μm)	-0.456	0.0051
R_{sm}	Total approach (μm)	+0.286	0.0027
R_{sm}	K_s (MPa/μm)	-0.286	0.0027
R_{sm}	K_t (MPa/μm)	-0.494	0.0055

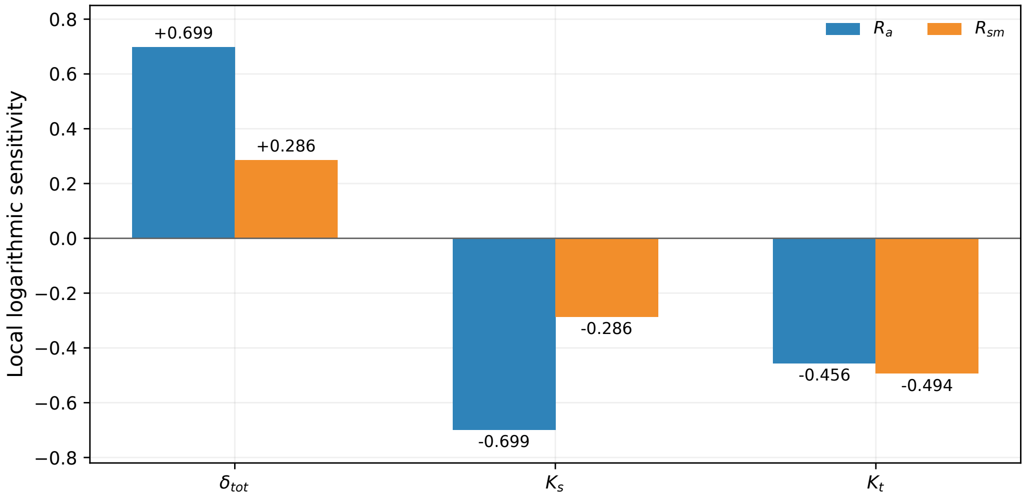

Figure S1. Local logarithmic sensitivities of total approach δ_{tot}, secant stiffness K_s and tangent stiffness K_t to R_a and R_{sm} for relaxed class A in the HS formulation at $q_{nom} = 1$ MPa. The dependent summit parameters are recomputed from the prescribed statistical closure for each perturbation. Bars use relative step $h = 0.01$; Table S20 reports the values and checks at $h = 0.005$ and 0.02.

S7.2. Engineering Regime Criteria

The dimensionless criteria distinguish the coating contribution, finite-thickness effect, contact scale, proximity to closure and relevant time scales using the prescribed model parameters. HS and FT values at the same geometry and material state compare substrate-confinement effects within the independent-asperity framework. Table S21 defines the criteria, and Table S22 gives the class A values.

Each regime ratio uses the same pressure, material state and contact branch. In particular, $\Pi_c = \delta_l/\delta_b$ compares the coated-minus-bare increment with the full base approach, while $\chi_{rmr} = \delta_{rmr}/\delta_b$ gives the Fourier share of that base. The reference time ratios are $\Theta_b = 1.786$ and $\Theta_l = 1.970$; Θ_b characterizes the HS quasi-elastic base approximation. Values $\Pi_q > 1$ denote formal continuation outside the open-texture domain. The contact-regime indicators characterize the assumed independent-asperity response; quantifying interaction error requires an interacting-asperity reference solution.

Table S21. Dimensionless regime criteria evaluated at the same pressure, material state and contact branch unless stated otherwise.

Criterion	Definition	Interpretation
Π_c	δ_l/δ_b	Ratio of the coated-minus-bare increment to the full base approach.
Φ_l	$\delta_l/\delta_{tot} = \Pi_c/(1+\Pi_c)$	Coated-minus-bare fraction of total approach at the specified endpoint state.
Π_h	$1 - \delta^{FT}_{plateau}/\delta^{HS}_{plateau}$	Relative finite-thickness correction to the plateau approach.
Π_a	a_c/t_l	Ratio of the characteristic asperity-contact radius to coating thickness.
Π_q	q_{nom}/q^*	Proximity to closure for the same branch and endpoint state.
Θ_b	$t_c/\tau_{ret,b}$	Ratio of the prescribed loading duration to the HS quasi-elastic base retardation scale.
Θ_l	$t_c/\tau_{c,l}$	Ratio of the prescribed loading duration to the scalar cell-increment retardation time.
χ_{rmr}	$\delta_{\mathrm{rmr}}/\delta_b$	Fourier fraction of the base approach.

Table S22. Dimensionless regime indicators. The pressure-independent time ratios are $\Theta_b \approx 1.79$ and $\Theta_l \approx 1.97$ at τ = 250 μs.

Class	State	Branch	q_{nom} (MPa)	Π_c	Φ_l (%)	Π_h	Π_a	Π_q	Domain
A	Inst.	HS	0.1	0.00103	0.103	0.248	0.997	0.0241	Open
A	Inst.	FT	0.1	0.00137	0.137	0.248	1.08	0.00545	Open
A	Inst.	HS	1	0.00585	0.581	0.304	1.67	0.241	Open
A	Inst.	FT	1	0.00837	0.83	0.304	1.46	0.0545	Open
A	Inst.	HS	1.85	0.00893	0.885	0.342	2.08	0.446	Open
A	Inst.	FT	1.85	0.0135	1.33	0.342	1.66	0.101	Open
A	Inst.	HS	3	0.0121	1.2	0.382	2.49	0.722	Open
A	Inst.	FT	3	0.0194	1.91	0.382	1.86	0.164	Open
A	Inst.	HS	5	0.0164	1.61	0.432	2.98	1.2	Formal post-closure
A	Inst.	FT	5	0.0284	2.76	0.432	2.15	0.273	Open
A	Rel.	HS	0.1	0.00105	0.105	0.257	1.01	0.0267	Open
A	Rel.	FT	0.1	0.00141	0.14	0.257	1.09	0.00575	Open
A	Rel.	HS	1	0.00589	0.585	0.318	1.73	0.267	Open
A	Rel.	FT	1	0.00859	0.852	0.318	1.49	0.0575	Open
A	Rel.	HS	1.85	0.00894	0.886	0.359	2.17	0.495	Open
A	Rel.	FT	1.85	0.0138	1.36	0.359	1.69	0.107	Open
A	Rel.	HS	3	0.0121	1.2	0.4	2.59	0.802	Open
A	Rel.	FT	3	0.0199	1.95	0.4	1.9	0.173	Open
A	Rel.	HS	5	0.0162	1.6	0.452	3.1	1.34	Formal post-closure
A	Rel.	FT	5	0.029	2.82	0.452	2.2	0.288	Open

For relaxed class A at $q_{nom} = 1$ MPa, the coated-minus-bare share of total approach is below 1% in both branches. The plateau correction is 31.8%, and the characteristic contact radius is of the same order as the 50 μm coating thickness. The corresponding HS quasi-elastic and scalar coated-minus-bare transition measures are defined in Section S2.

S7.3. Paired Azimuthal Response

The paired comparison evaluates $q_{nom}(\theta) = q_0[1 + \varepsilon\cos(n\theta + \alpha)]$ at the common pressure $q_0 = 2.991$ MPa for every class, endpoint state and branch. The hub/cam, mandrel-tooth and three-roller ovality patterns use amplitudes 0.10, 0.05 and 0.15, modes 1, 12 and 2, and phases 0, 0.30 and 0.15 rad, respectively. These names identify illustrative prescribed pressure fields. Actual device loads require a contact-equilibrium solution for the corresponding mechanical system.

Table S23 gives all eight combinations of class A/B, instantaneous/relaxed state and HS/FT branch, with three stiffness spreads for each combination. All 24 comparisons therefore use matched imposed pressure fields, whose largest excursion remains within the open domain of every branch and state. Figure S2 summarizes relaxed class A. The spreads increase from the mandrel-tooth pattern through the hub/cam pattern to ovality, and the FT value exceeds the corresponding HS value in every case.

The spread uses the midpoint of the angular extrema, $S_K = 200(K_{s,max} - K_{s,min})/(K_{s,max} + K_{s,min})$. For small ε, the local relation $d\ln K_s/d\ln q = 1 - K_s/K_t$ gives $S_K \simeq 200\varepsilon(1 - K_s/K_t)$. The reported spreads follow from the full nonlinear response, with the local-slope estimate providing their small-amplitude interpretation.

Table S23. Azimuthal secant-stiffness spreads for identical prescribed pressure patterns at $q_0 = 2.991$ MPa. Each spread is $S_K = 200(K_{s,\max} - K_{s,\min})/(K_{s,\max} + K_{s,\min})$. The peak-to-peak pressure ranges are 20%, 10% and 30%, respectively.

Class	State	Branch	Hub/cam S_K (%)	Mandrel S_K (%)	Three-roller S_K (%)
A	Inst.	HS	12.17	6.07	18.32
A	Inst.	FT	14.75	7.37	22.18
A	Rel.	HS	11.95	5.96	18.00
A	Rel.	FT	14.70	7.34	22.11
B	Inst.	HS	12.76	6.37	19.21
B	Inst.	FT	15.30	7.64	22.99
B	Rel.	HS	12.56	6.26	18.90
B	Rel.	FT	15.29	7.64	22.98

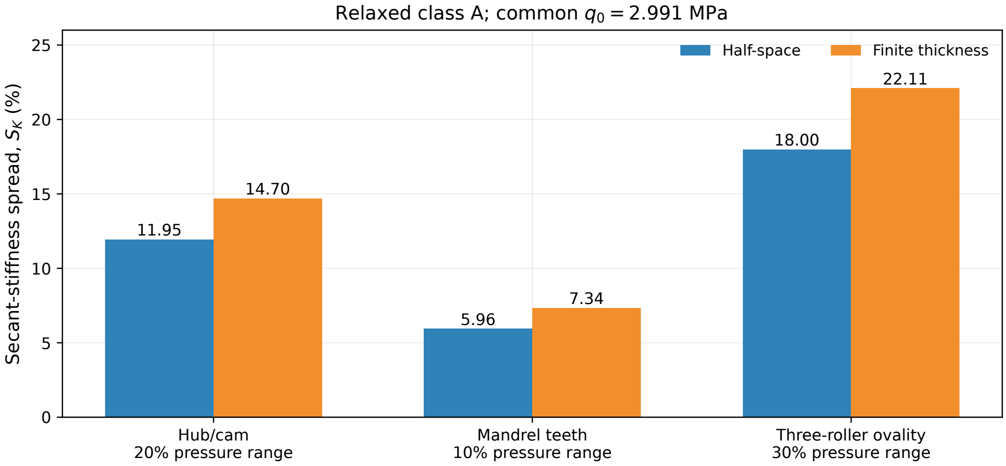

Figure S2. Secant-stiffness spreads for three prescribed nominal-pressure patterns in relaxed class A. HS and FT branches use identical pressure fields with common $q_0 = 2.991$ MPa and peak-to-peak pressure ranges of 20%, 10% and 30% for the hub/cam, mandrel-tooth and three-roller patterns. The spread is $S_K = 200(K_{s,max} - K_{s,min})/(K_{s,max} + K_{s,min})$. Table S23 includes both texture classes and both endpoint states. The patterns are illustrative prescribed loads.

S7.4. Contact-Regime Characterization of the Half-Space and Finite-Thickness Formulations

The contact-regime quantities follow the definitions in Section 2.5 of the main text. Table S24 reports the full matrix for both texture classes, both endpoint states, and both rough-contact branches at $q_{nom} = 0.1, 1.0, 1.852$, and $5.0\,\mathrm{MPa}$.

Table S24. (a) Contact-population and real-area measures at the prescribed pressure samples. † denotes a formal continuation beyond the closure pressure of the indicated branch and endpoint state.

Class	State	Branch	q_{nom} (MPa)	A_r/A_{land} (%)	A_r/A_{cell} (%)	f_e (%)	n_c (mm^{-2})
A	Inst.	HS	0.100	1.289	1.026	34.64	1.384
A	Inst.	HS	1.000	8.926	7.108	94.15	3.761
A	Inst.	HS	1.852	14.115	11.240	99.40	3.971
A	Inst.	HS	5.000†	28.175	22.436	100.00	3.995
A	Inst.	FT	0.100	0.669	0.533	14.80	0.591
A	Inst.	FT	1.000	4.559	3.630	57.08	2.280
A	Inst.	FT	1.852	7.344	5.848	73.46	2.934
A	Inst.	FT	5.000	14.934	11.892	93.98	3.754
A	Rel.	HS	0.100	1.419	1.130	37.05	1.480
A	Rel.	HS	1.000	9.695	7.720	95.65	3.821
A	Rel.	HS	1.852	15.253	12.146	99.67	3.981
A	Rel.	HS	5.000†	30.323	24.146	100.00	3.995
A	Rel.	FT	0.100	0.715	0.569	15.41	0.616
A	Rel.	FT	1.000	4.829	3.845	58.48	2.336
A	Rel.	FT	1.852	7.750	6.171	74.76	2.986
A	Rel.	FT	5.000	15.651	12.463	94.56	3.777
B	Inst.	HS	0.100	1.091	1.047	30.74	1.228
B	Inst.	HS	1.000	7.721	7.412	90.91	3.632
B	Inst.	HS	1.852	12.321	11.829	98.58	3.938
B	Inst.	HS	5.000	24.801	23.810	100.00	3.995
B	Inst.	FT	0.100	0.570	0.547	13.06	0.522
B	Inst.	FT	1.000	3.928	3.771	52.23	2.086
B	Inst.	FT	1.852	6.370	6.115	68.53	2.738
B	Inst.	FT	5.000	13.150	12.624	91.20	3.643
B	Rel.	HS	0.100	1.202	1.154	32.95	1.316
B	Rel.	HS	1.000	8.400	8.064	92.88	3.710
B	Rel.	HS	1.852	13.334	12.801	99.12	3.959
B	Rel.	HS	5.000†	26.703	25.636	100.00	3.995
B	Rel.	FT	0.100	0.610	0.586	13.61	0.544
B	Rel.	FT	1.000	4.165	3.999	53.61	2.141
B	Rel.	FT	1.852	6.731	6.462	69.89	2.792
B	Rel.	FT	5.000	13.800	13.248	91.94	3.673

Table S24. (b) Contact length and spacing measures for the same samples. † denotes a formal continuation beyond the closure pressure of the indicated branch and endpoint state.

Class	State	Branch	q_{nom} (MPa)	a_c (μm)	a_c/R_{sm}	χ_a
A	Inst.	HS	0.100	49.83	0.100	0.117
A	Inst.	HS	1.000	83.30	0.167	0.323
A	Inst.	HS	1.852	104.05	0.208	0.415
A	Inst.	HS	5.000†	149.07	0.298	0.596
A	Inst.	FT	0.100	53.84	0.108	0.083
A	Inst.	FT	1.000	73.16	0.146	0.221
A	Inst.	FT	1.852	82.88	0.166	0.284
A	Inst.	FT	5.000	107.50	0.215	0.417
A	Rel.	HS	0.100	50.60	0.101	0.123
A	Rel.	HS	1.000	86.45	0.173	0.338
A	Rel.	HS	1.852	108.35	0.217	0.432
A	Rel.	HS	5.000†	154.76	0.310	0.619
A	Rel.	FT	0.100	54.54	0.109	0.086
A	Rel.	FT	1.000	74.44	0.149	0.228
A	Rel.	FT	1.852	84.48	0.169	0.292
A	Rel.	FT	5.000	109.86	0.220	0.427
B	Inst.	HS	0.100	48.59	0.097	0.108
B	Inst.	HS	1.000	78.35	0.157	0.299
B	Inst.	HS	1.852	97.05	0.194	0.385
B	Inst.	HS	5.000	139.64	0.279	0.558
B	Inst.	FT	0.100	52.86	0.106	0.076
B	Inst.	FT	1.000	70.78	0.142	0.204
B	Inst.	FT	1.852	79.58	0.159	0.263
B	Inst.	FT	5.000	101.78	0.204	0.389
B	Rel.	HS	0.100	49.30	0.099	0.113
B	Rel.	HS	1.000	81.14	0.162	0.313
B	Rel.	HS	1.852	101.03	0.202	0.402
B	Rel.	HS	5.000†	145.03	0.290	0.580
B	Rel.	FT	0.100	53.54	0.107	0.079
B	Rel.	FT	1.000	71.98	0.144	0.211
B	Rel.	FT	1.852	81.08	0.162	0.271
B	Rel.	FT	5.000	103.99	0.208	0.399

Table S24. (c) Deformation, stress and closure indicators. Π_q uses the unrounded closure pressure of the same branch and endpoint state. † denotes a formal continuation beyond the closure pressure of the indicated branch and endpoint state.

Class	State	Branch	q_{nom} (MPa)	$10^5\chi_s$	$10^4\chi_E$	Π_q	Domain
A	Inst.	HS	0.100	1.82	0.285	0.0241	Open
A	Inst.	HS	1.000	4.64	2.850	0.2406	Open
A	Inst.	HS	1.852	6.95	5.278	0.4456	Open
A	Inst.	HS	5.000†	13.79	14.250	1.2030	Formal

Table S24 (c, continued)

Class	State	Branch	q_{nom} (MPa)	$10^5\chi_s$	$10^4\chi_E$	Π_q	Domain
A	Inst.	FT	0.100	1.38	0.285	0.0055	Open
A	Inst.	FT	1.000	2.37	2.850	0.0545	Open
A	Inst.	FT	1.852	2.94	5.278	0.1010	Open
A	Inst.	FT	5.000	4.61	14.250	0.2726	Open
A	Rel.	HS	0.100	1.87	0.318	0.0267	Open
A	Rel.	HS	1.000	4.96	3.176	0.2675	Open
A	Rel.	HS	1.852	7.49	5.881	0.4954	Open
A	Rel.	HS	5.000†	14.84	15.878	1.3374	Formal
A	Rel.	FT	0.100	1.39	0.318	0.0058	Open
A	Rel.	FT	1.000	2.41	3.176	0.0575	Open
A	Rel.	FT	1.852	3.00	5.881	0.1065	Open
A	Rel.	FT	5.000	4.72	15.878	0.2876	Open
B	Inst.	HS	0.100	1.74	0.236	0.0190	Open
B	Inst.	HS	1.000	4.16	2.364	0.1896	Open
B	Inst.	HS	1.852	6.12	4.378	0.3511	Open
B	Inst.	HS	5.000	12.14	11.820	0.9478	Open
B	Inst.	FT	0.100	1.33	0.236	0.0035	Open
B	Inst.	FT	1.000	2.23	2.364	0.0346	Open
B	Inst.	FT	1.852	2.74	4.378	0.0641	Open
B	Inst.	FT	5.000	4.19	11.820	0.1730	Open
B	Rel.	HS	0.100	1.78	0.263	0.0211	Open
B	Rel.	HS	1.000	4.43	2.634	0.2107	Open
B	Rel.	HS	1.852	6.58	4.878	0.3902	Open
B	Rel.	HS	5.000†	13.07	13.170	1.0534	Formal
B	Rel.	FT	0.100	1.35	0.263	0.0036	Open
B	Rel.	FT	1.000	2.27	2.634	0.0358	Open
B	Rel.	FT	1.852	2.79	4.878	0.0664	Open
B	Rel.	FT	5.000	4.29	13.170	0.1792	Open

Table S24 evaluates contact regimes at the pressure samples specified in this section, independently of the common azimuthal pressure q_0. Each closure ratio uses the corresponding branch- and state-specific closure pressure. The two contact-area normalizations satisfy $A_r/A_{cell} = (1 - F_{union})A_r/A_{land}$. The transient area in Section S10.1 is evaluated at the stated time and discretization. Area fractions and the normalized contacting-summit fraction f_e describe different population measures, and each is reported with its own denominator.

The small values of χ_s and χ_E are consistent with the small-deformation assumptions of the elastic single-asperity formulations. The contacting-summit fractions and the ratio of mean contact diameter to contacting-summit spacing, χ_a, characterize the potential for asperity interaction in the reference case, consistent with the established limits of narrow-scale Greenwood–Williamson representations [3–5]. They do not quantify the interaction error. The branch and state trends therefore provide a comparative characterization of the prescribed reference case.

S8. Elastic Single-Asperity Literature Cross-Check

To isolate the layered single-asperity kernel from the statistical rough-contact summation, the frictionless single-coating spherical-contact cases of Yu et al. [6] were reproduced with coating thickness $h = a_0$ (denoted t_l in the present model), sphere radius $R = 200a_0$, Poisson's ratio $\nu = 0.3$, and modulus ratios $E_l/E_s = 0.25$–4. The load P was selected so that the homogeneous substrate has the Hertz contact radius a_0. The normalizing pressure $P_0 = 2E_s^* a_0/(\pi R)$ is the corresponding Hertz peak pressure, with $E_s^* = E_s/(1 - \nu_s^2)$. The normalized pressure-profile comparison is shown in Appendix C.2 of the main text; Table S25 provides the corresponding radius and peak-pressure values. Global approach is not compared because Yu et al. use a substrate half-space, whereas the present implementation retains a finite fixed-bottom substrate.

Table S25. Elastic single-asperity literature cross-check against approximate digitizations of Yu et al. (2014) [6], Figure 3. The comparison is restricted to normalized contact radius and peak pressure; Yu values are graphical estimates.

E_l/E_s	Yu a/a_0	Present a/a_0	Δa (%)	Yu $p_{\max}/P_0$	Present $p_{\max}/P_0$	$\Delta p_{\max}$ (%)
4.00	0.650	0.6581	+1.24	2.271	2.2835	+0.55
2.00	0.807	0.8185	+1.42	1.479	1.4755	-0.24
1.00	1.000	1.0000	-0.00	1.000	1.0000	+0.00
0.50	1.196	1.2066	+0.88	0.700	0.7047	+0.67
0.25	1.435	1.4469	+0.83	0.507	0.5072	+0.03

Across the five modulus ratios in Table S25, the normalized contact radius and peak pressure agree with the approximate graphical reference values within 1.5%. This comparison concerns these two scalar observables. The pressure-profile comparison contains four groups labeled $E_l/E_s = 0.25$, 0.5, 1 and 2. Their mean absolute differences, normalized by the respective source peak, are approximately 0.758%, 0.656%, 0.291% and 0.643%; the largest pointwise differences are 11.16%, 12.48%, 1.38% and 16.98%. The homogeneous case recovers the Hertz radius and peak pressure within 0.004%, while discretization and substrate-depth checks change these scalar observables by less than 0.024%. These pointwise profile differences accompany the scalar contact comparison summarized in Section S11.1.

The comparison assesses elastic single-asperity contact over $E_l/E_s = 0.25$–4 against approximate graphical digitizations of published results. The reference coating contrast, approximately 0.05, is addressed separately by limiting-case and discretization checks. Agreement in normalized contact radius and pressure supports this elastic component of the formulation. Section S10.1 assesses local hereditary contact, and Section S11 discusses comparisons with published experiments.

S9. Illustrative Mechanical-Series Response and Kinematic Profile Mapping

This section presents the azimuthal pressure-to-stiffness transfer and a separate mechanical-series illustration. For relaxed class A at the common $q_0 = 2.991$ MPa, the local pressure-to-secant-stiffness transfer factors are approximately 0.596 in HS and 0.734 in FT. The exact identity is

$$\frac{d\ln K_s}{d\ln q} = 1 - \frac{q\delta'(q)}{\delta(q)} = 1 - \frac{K_s}{K_t}. \tag{S22}$$

Table S26(a) gives the paired responses and refers to the prescribed fields defined in Section S7.3. Figure S2 summarizes the corresponding stiffness spreads.

The roller–crown–spring example uses prescribed secant coefficients of 31.87, 98.30 and 100.0 N/μm at a preloaded reference state. The additional mechanical interference is $\Delta = \delta_0 = 20\,\mu$m, measured from that equilibrium and distinct from the residual texture depth h_{res}. The incremental secant coefficient is $K_{eq} = (P_{tot} - P_{pre})/\Delta = 19.4$ N/μm, the tangent series stiffness is 20.98 N/μm, and the total-force/interference ratio is $P_{tot}/\Delta =$ 20.40 N/μm. The incremental relation $\Delta P_i = K_{eq}\Delta g_i$ uses the preload-subtracted coefficient, while P_{tot}/Δ includes the preload. Converting nominal-area contact stiffness to these force-based coefficients requires a specified mechanical contact area.

For the kinematic illustration, $q(\theta) = q_0[1 + m(\theta)]$ and $e(\theta) = \delta_0 m(\theta)$ prescribe equal relative variations in nominal pressure and imposed interference. The profile is defined by this assumed mapping; the contact law $\delta_{tot}(q)$ is evaluated separately. A single harmonic with amplitude $m_0 = 0.10$ gives the peak-to-peak profile range $2m_0\delta_0 = 4\,\mu$m. The assumed $5\,\mu$m screening threshold is exceeded when $m_0 > 0.125$. Table S26(b) summarizes this illustrative mechanical scenario.

Table S26. (a) Relaxed class A local transfer at the common $q_0 = 2.991$ MPa. $K_s = q/\delta_{\text{tot}}$, $K_t = (\text{d}\delta_{\text{tot}}/\text{d}q)^{-1}$. Pattern spreads are listed in Table S23.

Branch	Quantity	Value
HS	Total approach (μm)	2.46
HS	K_s (MPa/μm)	1.21
HS	K_t (MPa/μm)	3.01
HS	1 - K_s/K_t	0.596
FT	Total approach (μm)	1.51
FT	K_s (MPa/μm)	1.98
FT	K_t (MPa/μm)	7.46
FT	1 - K_s/K_t	0.734

Table S26. (b) Illustrative mechanical-series and kinematic profile quantities. Force stiffnesses in N/μm are distinct from pressure stiffnesses in MPa/μm.

Quantity	Value	Definition
Incremental secant series stiffness	$K_{eq} = 19.4\,$N/μm	$(P_{tot} - P_{pre})/\Delta$; $\Delta P_i = K_{eq}\Delta g_i$
Tangent series stiffness	20.98 N/μm	K_t^{series} at the reference state
Total-force/interference coefficient	20.40 N/μm	P_{tot}/Δ, including the preload
Component secant stiffnesses	31.87, 98.30, 100.0 N/μm	Contact zone, crown, and spring, respectively
Kinematic hub/cam profile	4.00 μm range	$\pm$10% nominal-pressure modulation; 5.0 μm screening threshold, exceeded for $m_0 > 0.125$.

S10. Additional Histories, Boundary Conditions and Model Scope

S10.1. Hereditary Local FT Loading and Unloading

The local FT/GW history uses a 50 μm coating, a 2.70 mm substrate and material relaxation time $\tau = 250\,\mu$s. The stack compliance is evaluated with the operational moduli defined in the main text and approximated by a positive retardation expansion using 768 wavenumber quadrature nodes and ten retardation rates. The largest relative compliance error over the 99 test evaluations at Laplace arguments excluded from fitting is 5.39×10^{-8}. Section S11.1 describes the fits at additional wavenumbers and defines the two normalized

compliance errors; the individual spectral records are provided in the Supplementary Archive. This assessment concerns the compliance approximation; contact-discretization errors are evaluated separately.

With dimensionless time $x = t/\tau$, annular-force memory variables satisfy

$$\frac{d\mathbf{z}_l}{dx} + r_l\mathbf{z}_l = r_l\mathbf{f}, \qquad \mathbf{w} = \mathbf{Q}_0\mathbf{f} + \sum_l \mathbf{Q}_l\mathbf{z}_l.$$

Here l indexes the retardation modes, $r_l > 0$ is a dimensionless retardation rate, and τ/r_l is its dimensional retardation time. The vector $\mathbf{f}$ contains annular normal forces in N, and $\mathbf{z}_l$ is the corresponding force-memory vector, also in N. The matrices $\mathbf{Q}_0$ and $\mathbf{Q}_l$ are the instantaneous and modal delayed annular compliances, respectively, both in mm/N; $\mathbf{w}$ contains the annular normal displacements. Geometry and displacements are computed in mm and reported in micrometers. Over a step with $h = r_l\Delta x$, exact integration for a piecewise-linear force history gives

$$\mathbf{z}_{l,n} = e^{-h}\mathbf{z}_{l,n-1} + a_l\mathbf{f}_n + b_l\mathbf{f}_{n-1}, \quad a_l = 1 - \frac{1-e^{-h}}{h}, \quad b_l = \frac{1-e^{-h}}{h} - e^{-h}.$$

At a prescribed plane approach, the contact problem enforces $\mathbf{f} \geq 0$, $\mathbf{g} = \mathbf{w} + \mathbf{profile} - \delta_{plane}\mathbf{1} \geq 0$ and $f_i g_i = 0$ on annulus-averaged quantities. Under held loading, the common plane approach is adjusted so that the Gaussian population transmits the prescribed land pressure. During prescribed withdrawal, the active set can contain arbitrary subsets of annuli. The calculated histories retain contiguous active sets.

The fine spatial grid has 384 annuli and 480 summit-height nodes, with radial domain $r \leq 0.18$ mm and summit-height interval $[3\sigma - 1.30\,\mu\text{m}, 3\sigma]$. Lower summits remain out of contact over the reported approach range, so this numerical truncation preserves the prescribed Gaussian contact population. The time step is $\Delta t/\tau = 0.0125$, or 3.125 μs, with nodes placed explicitly at changes in the loading protocol.

On this grid, the two loading protocols start from an unstressed stack at a fixed contact location and maintain a nominal pressure of 1 MPa until $t_c = 0.5093$ ms. In protocol P (instantaneous retraction and pressure release), the plane is immediately retracted to $\delta_{plane} = -1\,\mu$m relative to the initial highest summit, causing complete loss of contact and zero contact pressure. Memory states remain continuous and subsequently decay as $\mathbf{z}_l(t) = \mathbf{z}_l(t_c)e^{-r_l(t-t_c)/\tau}$. In protocol W (prescribed linear withdrawal), the plane moves linearly from $\delta_{plane}(t_c)$ to the same final position over $0.5\tau = 0.125$ ms, with the contact reaction determined throughout withdrawal. Both histories describe quasistatic surface recovery under prescribed plane motion. Translating contact requires its spatial and temporal loading history, and free counterface rebound requires equations of motion including inertia.

Table S27(a) gives the held response and static endpoints on the same 384/480 grid. The plateau approaches at 0^+, t_c and ∞ are 1.116, 1.127 and 1.129 μm. Combining this hereditary plateau with the separately defined Fourier and scalar coated-minus-bare operators gives total approaches of 1.139, 1.151 and 1.154 μm. At t_c, base endpoint progress is 83.99% and total-approach growth is 1.07%. The instantaneous and relaxed plateau endpoints differ from the elastic pressure-basis results by approximately 0.00487% and 0.00523%, respectively. Each history is normalized by the static endpoints of its own discretization.

Immediately before unloading, $A_r/A_{cell} = 3.816\%$, where A_{cell} is the nominal cell area. Immediately after the pressure release in P, the maximum surface displacement is 0.045708 μm; after another 12τ, it is $1.018 \times 10^{-6}\,\mu$m. Table S27(b) reports contact reaction, contact area and surface recovery for each protocol. During W, contact disappears within (0.571772, 0.574897] ms on the plotted grid. The time-refined 192/240 grid with

$\Delta t/\tau = 0.00625$ gives $(0.571772, 0.573334]$ ms. These intervals bracket the event at the resolution of their respective grids. After detachment, surface recovery replaces total contact approach and stiffness as the reported observable.

The fine 384/480 history has minimum pressure zero, minimum annulus-averaged gap approximately -4.77×10^{-15} μm and maximum pressure–gap product 5.10×10^{-13} MPa μm. Refinement from 192/240 to 384/480 gives maximum displacement and reaction differences of 3.83×10^{-5} μm and 7.25×10^{-6} MPa. The 192/240 time-step refinement gives 3.60×10^{-6} μm and 1.14×10^{-6} MPa. Maximum absolute refinement differences are reported; their sequence is nonmonotonic. An independent nonnegative least-squares solution checks the active-set implementation for a selected contact state. Separate elastic receding Hertz/GW and analytical flat-punch pulse checks have maximum relative errors of approximately 9.31×10^{-5} and 4.44×10^{-16}, respectively. The flat-punch test isolates memory integration at fixed contact area; the receding-contact checks address the evolving contact set.

Figure S3(a) uses the 192/240 held curve, normalized by its own endpoints, while panels (b–d) use the 384/480 unloading histories. The annotation in (a) denotes plateau progress relative to the endpoints of that grid. The annulus-averaged memory-displacement vector at each summit-height node is $\mathbf{w}_{\mathrm{mem}}(t) = \sum_l \mathbf{Q}_l \mathbf{z}_l(t)$; panel (d) shows its maximum over all annuli and summit-height nodes against time from the start of unloading. During contact in W, the surface displacement also includes the instantaneous elastic pressure contribution. Once pressure vanishes, the memory contribution equals the full displacement. These local, quasistatic histories assume independent asperities and exclude feedback from the deterministic texture.

Table S27. (a) Hereditary FT held-step landmarks at 1 MPa and $\tau = 250$ μs. The discretization uses 384 annuli and 480 summit-height nodes with its own static endpoints. The base includes the exact reduced-creep Fourier displacement increment; the additive total also includes the separately defined endpoint-matched coated-minus-bare increment.

State	$\delta_{\mathrm{plateau}}$ (μm)	δ_b (μm)	δ_{tot} (μm)	Base progress (%)	Total growth (%)
Inst.	1.11597	1.129665	1.13912	0.0000	0.00000
t_c	1.12651	1.14151	1.15129	83.9871	1.06838
Rel.	1.12851	1.14377	1.15360	100.000	1.27127

Table S27. (b) Local FT unloading landmarks for protocol P (instantaneous retraction and pressure release) and protocol W (prescribed linear withdrawal). Grid entries list the numbers of annuli and summit-height nodes, respectively; the time step is $\Delta t/\tau$. Contact area is normalized by the nominal cell area A_{cell}. P retracts the plane immediately to -1 μm at $t_c = 0.5093$ ms, causing complete loss of contact; W prescribes retraction to the same plane over 0.125 ms. Event brackets are reported at the resolution of their respective grids. Post-detachment recovery is quantified by local surface displacement under prescribed plane motion.

Protocol	Event	Observable	Value	Unit	Grid; step
P and W	t_c^-	q_{nom}	1.00000	MPa	384/480; 0.0125
P and W	t_c^-	A_r/A_{cell}	3.81599	%	384/480; 0.0125
P	t_c^+	$q_{\mathrm{nom}}; A_r/A_{cell}$	0; 0	MPa; 1	384/480; 0.0125
P	t_c^+	$\max w$	0.04570825	μm	384/480; 0.0125
P	$t_c + 12\tau$	$\max w$	1.01776×10^{-6}	μm	384/480; 0.0125

Table S27 (b, continued)

Protocol	Event	Observable	Value	Unit	Grid; step
W	Detachment bracket	Last positive / first zero contact	(0.571772, 0.574897]	ms	384/480; 0.0125
W	Time-refined bracket	Last positive / first zero contact	(0.571772, 0.573334]	ms	192/240; 0.00625

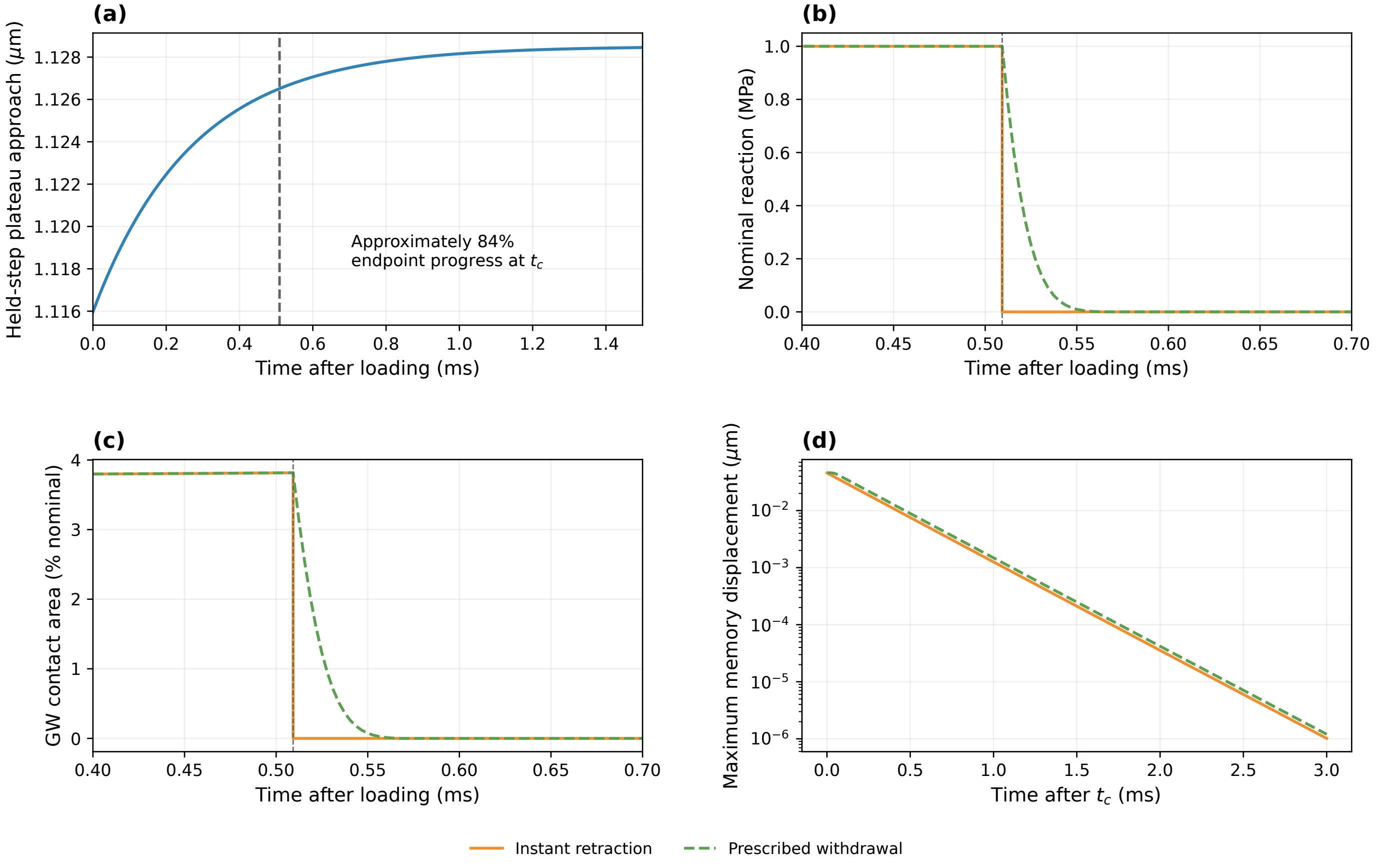

Figure S3. Hereditary local FT/GW loading and unloading for the class A reference with $\tau = 250\,\mu$s. (a) Plateau approach under a held nominal pressure of 1 MPa; progress at $t_c = 0.5093$ ms is normalized by the static endpoints of that grid. (b) Nominal reaction and (c) contact area as a percentage of the nominal cell area A_{cell} for protocol P (instantaneous retraction and pressure release) and protocol W (prescribed linear withdrawal), sharing the held loading up to t_c. P retracts the plane immediately to $-1\,\mu$m relative to the initial highest summit, causing complete loss of contact; W reaches that position over $0.5\tau = 0.125$ ms. (d) Maximum annulus-averaged displacement contributed by the retained memory modes over all annuli and summit-height nodes, measured after unloading begins; it equals the full surface displacement once contact pressure vanishes. Panel (a) uses 192 annuli and 240 summit-height nodes; panels (b–d) use 384 annuli and 480 summit-height nodes, all with $\Delta t/\tau = 0.0125$. Progress in (a) uses its own grid endpoints. The histories describe local quasistatic contact and surface recovery under prescribed plane motion.

S10.2. Macroscopic Boundary-Condition Matrix

The macroscopic boundary-condition study uses the deterministic class A cell with the prescribed geometry, material endpoints, coating thickness and substrate depth. It evaluates the rigid-platen displacement relative to the fixed normal bottom reference, δ_{pl}, and the matched coated-minus-bare increment, $\delta_l^{BC} = \delta_{pl,coated}^{BC} - \delta_{pl,bare}^{BC}$. The mean local

coating compression is defined separately by the top-to-interface displacement difference. Statistical land roughness is represented by the separate contact model.

Five BC variants are defined. OED is the reference with periodic displacement fluctuations, zero mean lateral normal strains, fixed bottom normal displacement and one tangential anchor. PS retains periodic fluctuations and $\bar{\varepsilon}_{yy} = 0$, while the mean x strain is free and satisfies $\bar{\sigma}_{xx} = 0$. FREE allows both mean lateral strains to vary under $\bar{\sigma}_{xx} = \bar{\sigma}_{yy} = 0$, while retaining periodic fluctuations. The FREE conditions apply to mean stresses, rather than pointwise tractions on lateral faces. CLAMP adds zero tangential displacement over the bottom of the OED cell, with redundant constraints removed. ROLLER replaces lateral periodicity by zero normal lateral displacement on the corresponding opposing side faces and retains the OED bottom reference.

For any observable R, the signed deviation is

$$D_R(BC) = 100\left[\frac{R_{BC}}{R_{OED}} - 1\right],$$

with the same load, material state, mesh and displacement reference in numerator and denominator. The primary five-BC comparison is at 1 MPa in the instantaneous state on coarse and medium meshes; the fine-mesh comparison covers the OED/FREE pair. The matrix contains 40 force-controlled cases. Additional pressures and the relaxed endpoint extend selected comparisons; the combinations of pressure, state, mesh and boundary condition are not fully factorial.

On the medium mesh, δ_{pl} is 0.03541 μm for OED, 0.04387 μm for PS and 0.04814 μm for FREE. The associated changes are 23.91% and 35.96%. CLAMP and ROLLER coincide numerically with OED under the tested symmetry and loading. In the fine-mesh OED/FREE comparison the platen change is 36.16%, while the matched coated-minus-bare increment changes by 5.78%. The maximum increment change over the evaluated matrix is 5.85% in the relaxed medium case. Each percentage refers to its stated observable, material state and discretization.

The fine mesh used in the BC study has 36 by 36 in-plane divisions, 18 substrate divisions and eight coating divisions; it is distinct from the fine discretization used in Table A1 of the main text. The medium-to-fine-mesh OED/FREE comparison satisfies tolerances of 1% for platen displacement and 2% for the coated-minus-bare increment. These tolerances are not met throughout the coarse-to-medium comparison. Independent flat-cell solutions for OED, PS and FREE agree with the corresponding analytical compliances to within approximately 0.0034%, and the maximum relative force-balance residual $|F_{\text{bottom}}/(q_{nom}A_{cell}) - 1|$ is 3.49×10^{-8}, where F_{bottom} is the total normal bottom reaction and $q_{nom}A_{cell}$ is the applied normal force. Contact pressure is nonnegative in the force-controlled cases. An auxiliary displacement-controlled calculation with tensile limit 10^{-8} MPa changes the OED/FREE displacements by less than 0.002%. These numerical checks support the specified cell comparisons; the supplementary archive records the mesh and contact settings.

The matrix quantifies the sensitivity of deterministic-cell observables to explicit boundary conditions. In the additive rough-contact formula, changing only δ_l while holding the local FT base fixed isolates the coated-minus-bare contribution. The full boundary-condition dependence would also require the corresponding response of the rough-contact base. Boundary-condition sensitivity and error from coupling between deformation fields are separate questions.

Table S28 summarizes the boundary-condition comparison; Figure S4 shows the associated displacement deviations.

Table S28. Platen displacement and matched coated-minus-bare increment for class A at 1 MPa in the instantaneous state. Here δ_{pl} is the coated-cell platen displacement and δ_l^{BC} is the matched coated-minus-bare increment. Deviations are $100(R_{BC}/R_{OED} - 1)$, using the same mesh, state and load. Medium rows cover five variants; fine-mesh rows cover OED/FREE on the fine mesh used in the BC study.

BC	Mesh	δ_{pl} (μm)	δ_l^{BC} (μm)	$D_{\delta_{pl}}$ (%)	D_{δ_l} (%)
OED	medium	0.03541	0.009452	0.0	0.0
PS	medium	0.04387	0.00981	23.9	3.8
FREE	medium	0.04814	0.00999	36.0	5.7
CLAMP	medium	0.03541	0.009452	0.0	0.0
ROLLER	medium	0.03541	0.009452	0.0	0.0
OED	fine	0.0352	0.009303	0.0	0.0
FREE	fine	0.04794	0.009841	36.2	5.8

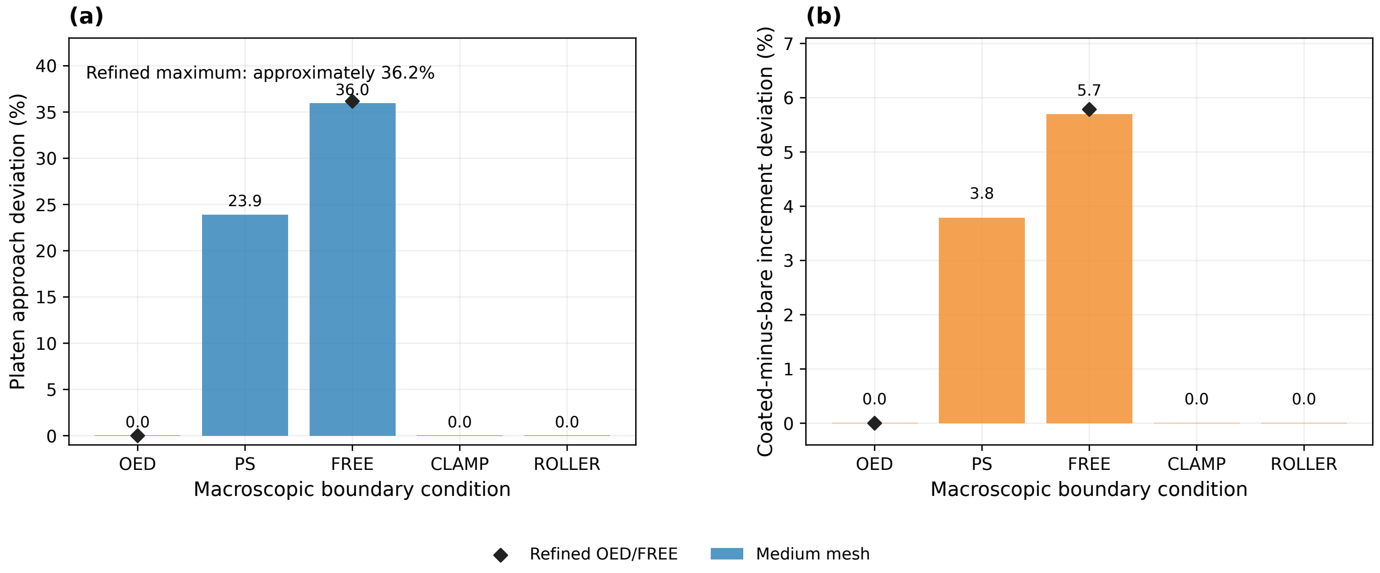

Figure S4. Sensitivity to macroscopic boundary conditions for the deterministic class A coating/substrate cell at $q_{nom} = 1$ MPa in the instantaneous state. (a) Downward platen displacement relative to the bottom reference and (b) the matched coated-minus-bare platen-displacement increment. Deviations are $D_R = 100(R_{BC}/R_{OED} - 1)$, using the same mesh, material state and load in each comparison. Bars show five BC variants on the medium mesh; diamonds show the fine-mesh OED/FREE pair. The largest fine-mesh platen deviation is approximately 36.2%. The boundary-condition definitions specify OED, PS, FREE, CLAMP and ROLLER. The deviations quantify the sensitivity of these deterministic-cell observables to lateral constraints.

S10.3. Coupling Diagnostics and Limits

The two-way homogenized calculation divides the compliance spectrum between a local FT/GW operator and a macroscopic finite-element response. Iteration enforces pressure compatibility and load balance for the selected split. Adding the low-frequency part to its algebraically defined complement recovers the full compliance by construction. Selecting a physically justified spectral division remains an additional modeling requirement.

For the selected fine-mesh calculation, numerical compatibility and the sampled cavity-rim gap conditions are satisfied. Independent modal comparisons with the homogeneous-layer compliance cover selected axial Fourier modes. The responses depend on the cutoff, and other tested splits fail the cavity-rim or short-wavelength-resolution conditions. The

restricted admissibility of the tested splits and the limited modal coverage preclude assigning a general coupling-error bound to the difference between the split-operator and additive results or to the isolated pressure-feedback change.

Table S29 summarizes four complementary diagnostics: a prescribed Fourier-term multiplier range, one-way pressure redistribution, a two-way split-operator calculation and a macroscopic boundary-condition matrix. Each evaluates a specified mechanism or numerical property. Together they support comparative interpretation within the stated additive model. A fully coupled reference for assessing the additive approximation would enforce equilibrium and normal displacement compatibility with common contact pressure, the same geometry and constitutive history, and matched interface, boundary and displacement-reference conditions. For nonadhesive frictionless contact, pressure and gap satisfy $p \geq 0$, $g \geq 0$ and $pg = 0$. The reference response must be resolved for the specified physical topography and independent of an arbitrary partition between component operators. Scale separation is not required by that physical problem; scale division and limited overlap instead require justification when using the additive approximation or a split-operator representation.

Table S29. Scope of complementary diagnostics for the additive formulation. Each diagnostic addresses a specified mechanism or numerical property.

Diagnostic	Scope	Remaining limitations
Fourier-term multiplier	Sensitivity of the additive response to a prescribed Fourier-term multiplier with the other terms fixed	Omitted roughness–texture feedback and its physical error remain unquantified
One-way pressure redistribution	One-way response to a prescribed spatially redistributed load	Self-consistent feedback requires a two-way equilibrium solution; a general additive-model correction remains undetermined
Two-way split-operator calculation	Force balance, compatibility and selected numerical checks within a specified separation of spatial scales	Physical scale separation and full-spectrum resolution remain to be established; the split-dependent discrepancy does not provide a general coupling-error bound
Macroscopic BC matrix	Changes of explicitly defined deterministic-cell observables under matched alternative constraints	The comparison covers the specified deterministic-cell observables, boundary conditions and elastic state

S10.4. Component Ratios and Their Denominators

The component ratios are defined as $\chi_{rmr} = \delta_{rmr}/\delta_b$ and $\Phi_l = \delta_l/\delta_{tot}$. For class A at 1 MPa, the instantaneous/relaxed HS Fourier shares of the base are approximately 0.847%/0.915%; the corresponding FT shares are 1.213%/1.334%. The instantaneous/relaxed coated-minus-bare shares of the total are approximately 0.581%/0.585% in HS and 0.830%/0.852% in FT. Table S30 reports the ratios with their respective denominators. The separate ratio $\Delta\delta_b/\Delta\delta_l$ compares the component endpoint changes. These definitions distinguish component shares at a given state from relative contributions to the transition.

Table S30. Class A component partition at 1 MPa: $\chi_{\mathrm{rmr}} = \delta_{\mathrm{rmr}}/\delta_b$, $\Phi_l = \delta_l/\delta_{\mathrm{tot}}$. The base combines the statistical land-roughness term and deterministic Fourier displacement increment; these ratios quantify the Fourier share of the base and the coated-minus-bare share of total approach.

State	Branch	$\delta_{\mathrm{plateau}}$ (μm)	δ_{rmr} (μm)	δ_l (μm)	χ_{rmr} (%)	Φ_l (%)
Inst.	HS	1.603	0.0137	0.00945	0.847	0.581
Inst.	FT	1.116	0.0137	0.00945	1.21	0.83
Rel.	HS	1.654	0.0153	0.00983	0.915	0.585
Rel.	FT	1.128	0.0153	0.00983	1.33	0.852

S11. Numerical verification and literature comparisons

S11.1. Numerical verification

Table S31 brings together the elastic contact comparisons, periodic-cell and Fourier discretization checks, prescribed-traction layer responses, local hereditary contact and finite-ramp convolution. Each assessment has a specified observable, reference and error definition.

Elastic layered literature comparison.

The elastic single-asperity comparison in Appendix C.2 of the main text and Section S8 uses the frictionless single-coating spherical-contact cases of Yu et al. (2014) [6], with $h = a_0$, $R = 200a_0$, $\nu = 0.3$ and $E_l/E_s = 0.25$–4. Table S25 compares normalized contact radius and peak pressure with approximate digitizations of the source's Figure 3; the largest tabulated differences are 1.42% and 0.67%, respectively. Numerical refinement and substrate-depth variation change these quantities by less than 0.024%. This 1.5% agreement is specific to the two scalar contact observables; Section S8 reports the pointwise profile differences and the comparison domain.

Periodic cells and class B stripe.

The periodic-cell mesh comparison covers six configurations and five class-specific pressures for each texture class (Appendix A and Figure 4 of the main text). Table S31 summarizes the mean-approach changes, normalized by the fine-mesh values. For the textured uncoated class A cells, the mesh differences in p_{95}/q_{ref} are 10.86–11.02%, with the fine-mesh ratio as denominator and q_{ref} the mean active-contact pressure on each mesh; they exceed the 3% criterion at every sampled pressure. Thus the integral-response comparison and local-pressure convergence have separate criteria. Appendix A also reports mesh differences in the area-weighted mean local coating compression over active contact. This through-thickness compression is distinct from the coated-minus-bare cell increment. Under matched periodic conditions at 1 MPa in the instantaneous state, the class B section/stripe comparison gives a relative difference of 0.00686% $<$ 0.01%, with the section value as denominator (Appendix B of the main text; Table S5).

Fourier cutoff.

The finite-cutoff comparison for the prescribed elliptical aperture is reported in Section S1.1 and Table S1. It assesses the deterministic uniform-pressure displacement increment over paired square cutoffs $N = 6$–192 with fixed geometry and normalization. The contact calculations use $N = 6$.

Hereditary unloading.

Section S10.1 and Table S27 report local hereditary FT/GW loading and prescribed withdrawal with material memory and unilateral contact conditions. They give the spatial and time-step comparisons as absolute differences in surface displacement and reaction. The flat-punch pulse isolates memory integration at fixed area, while elastic receding-contact and active-set comparisons assess contact-set evolution. These are local quasistatic component checks under prescribed plane motion.

Fixed-aperture pressure compatibility.

A separate deterministic control uses the class A 1×1 mm cell and a permanently traction-free elliptical aperture with semi-axes 0.246627 and 0.262905 mm. The one-sided homogeneous half-space operator is $\widehat{w} = 2\widehat{p}/(E^*k)$ for $k > 0$, with zero mean displacement. At fixed nominal load, pressure satisfying constant normal displacement on the smooth

lands is compared with uniform land pressure on the same raster mask. On the 1536×1536 Fourier grid, the respective normalized compliance lengths are 0.0455931 and 0.0607703 mm, a reduction of 24.97%. The last grid refinement changes the compatible value by 0.07786%; the increasing edge-pressure maximum remains unresolved. Relative to the adopted $N = 6$ uniform-pressure length, the reduction is 24.46%. Scaling only δ_{rmr} by the compatible-to-adopted compliance ratio in the 1 MPa elastic endpoints, with the other contributions fixed, increases K_s by 0.206–0.223% for HS and 0.295–0.325% for FT. These conditional stiffness changes are component-substitution estimates for deterministic pressure redistribution on fixed smooth lands. The model uses a permanently traction-free aperture and a homogeneous half-space operator, with the roughness and coated-minus-bare responses held fixed.

Prescribed-traction layer response.

The periodic bonded-layer benchmark in Section S1.3 uses a 0.50×0.50 mm cell, a centered circular opening with area fraction 0.392 and a 50 μm layer. The bottom is fully fixed, the lateral boundaries are periodic, and the upper surface has zero tangential traction, uniform normal traction on the land and zero traction over the opening. The bulk modulus is $K = 5000$ MPa and the shear modulus is 1380 or 1210 MPa. At Fourier cutoff $|m|, |n| \leq 128$, the instantaneous and relaxed land-averaged compliances per nominal pressure are 0.0122608 and 0.0128813 μm/MPa. Their ratios to $C_{1D} = t_l/[\phi E/(1-\nu^2)]$, where ϕ is the land-area fraction, are 0.656942 and 0.619410. The reference ratios are 0.657251 and 0.619186 (Table S4), giving signed differences of -0.0470% and $+0.0362\%$ relative to those references. Increasing the cutoff from 96 to 128 changes the ratios by 0.00172% and 0.00182%. This benchmark uses a contact-modulus reference C_{1D}, distinct from the local oedometric normalization in Figure 2(a) of the main text.

A separate modal benchmark compares the Navier transfer-matrix solution with the closed-form homogeneous-layer compliance [7] in Equation (S21), with h_s replaced by h and material properties E, ν. The bottom has zero normal and tangential displacement; the upper surface carries sinusoidal normal traction and zero tangential traction. The dimensionless response is EC_{zz}/h. The 30 cases combine $kh = 0.01, 0.03, 0.1, 0.3, 1, 3$ with $\nu = 0.30, 0.40, 0.45, 0.49, 0.499$, using $E = h = 1$ in the numerical evaluation. The maximum $|C_{Navier}/C_{closed} - 1|$ is 2.32×10^{-11}. This comparison assesses the displacement operator under prescribed traction. Table S14 complements it with sampled spectral modes for removal of the coating at fixed substrate depth; Sections S8 and S10.1 assess the elastic and hereditary contact discretizations.

Hereditary contact and compliance approximation.

The local hereditary calculation uses the FT stack and Gaussian summit population defined in Section S10.1. Table S31 gives its instantaneous and relaxed plateau approaches alongside the elastic pressure-basis values. The relative endpoint difference is $100(\delta_{hereditary\ endpoint}/\delta_{elastic} - 1)$, using the corresponding elastic endpoint as denominator. At $t_c = 0.509272$ ms, the plateau approach on a grid of 384 annuli and 480 summit-height nodes is 1.126512266 μm, corresponding to 84.0724% of its instantaneous-to-relaxed plateau change. The grid of 192 annuli and 240 summit-height nodes gives 1.126511223 μm, an absolute difference of 1.04×10^{-6} μm at the common time step $\Delta t/\tau = 0.0125$. The local contact, Fourier and coated-minus-bare contributions retain their defined displacement references.

The finite-stack compliance is represented by nonnegative retardation modes on 768 wavenumber quadrature nodes with ten rates. To assess the dependence on the dimensionless Laplace argument $x = s\tau$, separate weights are fitted at each of nine additional

wavenumbers using 28 real arguments, $x = 0$ and 27 logarithmically spaced values from 2×10^{-4} to 5×10^{3}. The resulting approximation is evaluated at seven other positive real arguments and four purely imaginary arguments, giving 99 comparisons. These arguments are excluded from the fit. The reference is a separate complex transfer-matrix evaluation with a bonded interface, a fixed lower boundary and zero upper tangential traction. For each layer of nominal thickness h (h_s for the substrate and t_l for the coating), both the training compliance and reference evaluation use the effective propagation thickness $\min(h, 18/k)$ at $k > 0$. This numerical truncation replaces h in the matrix exponential when $kh > 18$. The 99 comparisons assess the retardation approximation for this common operator; error introduced by the propagation-depth cap is outside their scope.

For the approximated and reference compliances C_{fit} and C_{ref}, the errors are $e_C = |C_{fit} - C_{ref}|/|C_{ref}|$ and $e_{\Delta C} = |C_{fit} - C_{ref}|/(C_\infty - C_0)$, where C_0 and C_∞ are the instantaneous and relaxed elastic compliances. The maxima over the 99 test comparisons are 5.39×10^{-8} and 7.03×10^{-6}, respectively, both at $k = 0.003\ \mathrm{mm}^{-1}$ and $x = 1.7i$. Both errors are dimensionless. The Supplementary Archive provides all 99 test coordinates and both normalized errors, together with a script that reproduces their aggregate statistics. This assessment concerns the Laplace-argument dependence of the spectral compliance approximation with weights fitted independently at each additional wavenumber. The accuracy of interpolation between the 768 wavenumber quadrature nodes requires a separate assessment. Contact observables and their discretization differences are evaluated in Sections S8 and S10.1.

Finite loading ramp.

For a unit linear load ramp of duration T, followed by a hold, the delayed response of a unit compliance mode $1 - \exp(-t/\tau_i)$ is

$$\begin{aligned} R_i(t) &= \frac{1}{T}\int_0^{\min(t,T)} [1 - e^{-(t-s)/\tau_i}]\,\mathrm{d}s \\ &= \begin{cases} [t - \tau_i(1 - e^{-t/\tau_i})]/T, & 0 \le t \le T, \\ 1 - A_i e^{-(t-T)/\tau_i}, & t > T, \end{cases} \\ A_i &= \frac{\tau_i}{T}(1 - e^{-T/\tau_i}). \end{aligned}$$

Time is measured from the start of the ramp. The retardation times, 7.9 and 109 s, are the published PL-1 values [8]; the ramp durations are 20, 50, 100 and 200 s. Each pair (T, τ_i) is evaluated at $t = T/4, T, T + 1\,\mathrm{s}, T + \tau_i, T + 120\,\mathrm{s}$, giving 40 cases. Adaptive quadrature with requested absolute and relative tolerances of 2×10^{-13} agrees with the analytical expression to a maximum absolute dimensionless difference of 2.23×10^{-16}. Unit final load and unit delayed-mode amplitude define the normalization. A complete linear compliance law adds its instantaneous contribution separately. This benchmark assesses finite-ramp convolution, including the hold-onset factor A_i; a material-specific indentation prediction also requires the modal amplitudes, geometry and actual loading history. For spherical linear-viscoelastic indentation under the applicable monotonic-contact assumptions, the hereditary observable is proportional to indentation depth raised to the power 3/2. Fixed-area contact instead has a linear displacement observable.

Observable used in Figure 2(a) of the main text.

For the fine instantaneous results, the bins centered near $\xi = 0.105$ and 0.145 have mean local compression of 0.010505 and 0.010304 μm, mean pressure of 1.3761 and 1.2984 MPa, and mean pointwise normalized compression of 1.0443 and 1.0863. Thus mean compression decreases by 1.91%, mean pressure by 5.64%, and the plotted normalized ordinate

increases by 4.02%. The ordinate is the area-weighted mean of pointwise ratios, with pointwise denominator $t_l p_{loc}/M_{oed}$. These data explain how the intermediate normalized rise can coexist with decreasing absolute compression. The physical contributions to the complete decrease–increase–decrease profile remain unresolved, and its extrema remain mesh sensitive. The percentages describe bin-to-bin changes.

Table S31. Numerical verification of the component responses. Annular-grid sizes are reported as numbers of annuli/summit-height nodes. Relative differences use the specified reference; percentages are identified explicitly. Displacement and reaction differences are absolute, with the units shown. Endpoint progress describes a normalized response, and the fixed-aperture stiffness change is a conditional component-substitution estimate.

Assessment	Response or reference	Result
Elastic layered contact [6]	Table S25: normalized radius and peak pressure; approximate graphical reference	Maximum differences 1.42% and 0.67%, respectively; pointwise profiles assessed separately
Periodic-cell mean approach	Appendix A of the main text: medium-to-fine comparison, normalized by the fine result	Maxima 0.217% (A) and 0.0496% (B); local pressure has a separate convergence criterion
Class B section and 3D stripe	Table S5: 0.03325999 and 0.03326227 μm, respectively	0.00686%, relative to the section value; below 0.01% for the matched case
Periodic Navier/Fourier response: instantaneous	C_{bonded}/C_{1D} = 0.656942; reference 0.657251 (Table S4)	−0.0470%, relative to the reference ratio
Periodic Navier/Fourier response: relaxed	C_{bonded}/C_{1D} = 0.619410; reference 0.619186 (Table S4)	+0.0362%, relative to the reference ratio
Homogeneous-layer modes [7]	30 transfer-matrix/closed-form comparisons; Equation (S21)	Maximum $\lvert C_{Navier}/C_{closed} - 1\rvert = 2.32 \times 10^{-11}$
FT compliance approximation	99 tests at Laplace arguments excluded from the fit, for nine additional wavenumbers; weights fitted separately at each wavenumber	$\max e_C = 5.39 \times 10^{-8}$; $\max e_{\Delta C} = 7.03 \times 10^{-6}$; dimensionless errors defined in the text
FT instantaneous endpoint	1.115967835 versus 1.115913513 μm	+0.00487%, relative to the elastic pressure-basis endpoint
FT relaxed endpoint	1.128509917 versus 1.128450868 μm	+0.00523%, relative to the elastic pressure-basis endpoint
Held FT contact: spatial refinement	192/240 to 384/480 at t_c; $\Delta t/\tau$ = 0.0125	Absolute plateau-approach difference 1.04×10^{-6} μm
Held FT contact: plateau progress	384/480 grid; t_c = 0.509272 ms	84.0724% of the plateau endpoint change on that grid; normalized plateau response
Hereditary withdrawal: spatial refinement	Table S27: 192/240 to 384/480; common time step	Maximum absolute history differences: surface displacement 3.83×10^{-5} μm; reaction 7.25×10^{-6} MPa
Fourier cutoff	Table S1: Λ_6 relative to Λ_{192}	−0.6756%; finite-cutoff comparison for prescribed uniform land pressure

Continued on the next page

Table S31 (continued)

Assessment	Response or reference	Result
Fixed-aperture pressure compatibility	Same 1536 × 1536 Fourier grid, raster mask and load: compatible pressure versus uniform land pressure	Deterministic compliance −24.97%; conditional total K_s changes +0.206–+0.325% across HS/FT endpoints
Finite-ramp convolution	40 analytical/quadrature comparisons for unit delayed modes	Maximum absolute dimensionless difference 2.23 × 10^{-16}

S11.2. Indentation-derived and comparison moduli

Oyen [8] compared indentation-derived shear moduli with values obtained from other measurements. Table S32 reports all four pairs from the source's Table I, including their units. PL-1, cured Epon and dry gelatin form the stiff-material class; urethane is a physically distinct soft rubber.

For each pair, the signed relative difference is $e_G = 100(G_{ind} - G_{comp})/G_{comp}$. The mean absolute relative difference is $n^{-1}\sum|e_G|$, and the maximum is max $|e_G|$. For all four materials, the mean and maximum are 37.12% and 114.29%; for the three stiff materials, they are 11.40% and 18.18%. These statistics use the printed modulus values.

The comparison values have different origins. The urethane value uses dynamic data and an assumed Poisson ratio; PL-1 and Epon use independently reported elastic properties; dry gelatin uses an assumed Poisson ratio of 0.35. The aggregate summarizes the reported modulus pairs from these different measurement methods. The PL-1 retardation times, 7.9 and 109 s, enter the unit-mode ramp benchmark in Section S11.1. A material-specific indentation history additionally requires the modal amplitudes, geometry and actual load history.

Table S32. Shear-modulus pairs reported by Oyen [8], Table I. All four materials are reported; the stiff-material class comprises PL-1, cured Epon and dry gelatin. Signed differences use the comparison modulus G_{comp} as denominator.

Material	G_{ind}	G_{comp}	e_G	Class
Urethane	1.5 MPa	0.7 MPa	+114.29%	Soft rubber
PL-1	1.3 GPa	1.1 GPa	+18.18%	Stiff
Epon	1.2 GPa	1.3 GPa	−7.69%	Stiff
Dry gelatin	1.3 GPa	1.2 GPa	+8.33%	Stiff

S11.3. Constrained modulus of a confined film

Gacoin et al. [9] considered frictionless spherical contact with a film bonded to an elastic substrate. In the sufficiently confined, compressible-film regime, the local thickness change follows $u_{top} - u_{bottom} = t\sigma/M$. Here u_{top} and u_{bottom} are normal displacements positive into the substrate, σ is the compressive normal stress taken positive in compression, t is the film thickness, and

$$M = \frac{E(1-\nu)}{(1+\nu)(1-2\nu)}.$$

The published glassy-film properties $E = 1200$ MPa and $\nu = 0.4$ give $M_{bulk} = 2571.43$ MPa, compared with $M_{contact} = 2800$ MPa inferred from contact. The descriptive difference $100(M_{contact} - M_{bulk})/M_{bulk}$ is 8.89% (Table S33). The source also gives the rounded bulk-based value 2500 MPa, which would yield 12%; the 8.89% comparison consistently uses the printed E, ν pair.

The approximation is illustrated near $a/t = 10$ for film $\nu = 0.4$ and substrate $\nu = 0.2$. Near incompressibility, shear deformation and the full layered solution become important,

making applicability dependent on compressibility as well as a/t. The local thickness-change relation concerns film compression. The present δ_l instead compares coated and bare periodic cells and also includes the induced change in substrate displacement.

Table S33. Constrained-modulus comparison for the compressible film studied by Gacoin et al. [9]. M_{bulk} follows from the published E, ν pair; the descriptive relative difference uses M_{bulk} as denominator.

Quantity	Value	Basis
E; ν	1200 MPa; 0.4	Bulk properties
M_{bulk}	2571.43 MPa	Constrained-strain expression
$M_{contact}$	2800 MPa	Contact-based estimate
Relative difference	8.89%	$100(M_{contact} - M_{bulk})/M_{bulk}$

S11.4. Confinement and interface conditions

Cheneler et al. [10] measured spherical indentation of polydimethylsiloxane (PDMS) layers with base-to-curing-agent volume ratios of 5:1, 10:1 and 20:1. The tests used a 28.5 mm diameter ball, a nominal approach speed of 1 mm/s and a 120 s hold at 18 °C and 40% relative humidity. At matched indentation depth and composition, thinner layers required larger forces. This trend supports the direction of finite-thickness stiffening; quantitative comparison additionally requires the experimental support condition to match the assumed bonded interface. The source's one- and three-mode fits characterize its material representation.

For the present class A calculation at $q_{nom} = 1$ MPa, the instantaneous total approaches are 1.626 and 1.139 μm for the half-space and finite-thickness branches. The 29.95% reduction is a model-to-model difference. It has the same direction as the thickness trend, while the different materials and loading protocols limit the comparison to that trend.

Johnson and Stafford [11] studied photopolymerized cross-linked methacrylate films on silane-treated glass with a steel sphere of radius 6.35 mm loaded by gravity at 22 °C. Their Supporting Information, Table S1, reports film thicknesses, contact radii and contact-inferred compliance. Table S34 reports all 15 points at 120 s for lauryl methacrylate (LMA) with 75% by mass of 1,6-hexanediol dimethacrylate (HDDMA). The reported bulk compliance is $(4.14 \pm 0.05) \times 10^{-9}$ Pa^{-1}.

The tabulated geometry spans $1.63 \leq a/t \leq 6.75$. The different confinement range and interface conditions require a separate applicability assessment from the compressible-film example near $a/t = 10$ in Section S11.3. The fluorinated interface has reduced adhesion and may separate; its tangential constraint requires its own interface description. The reported compliances are inverse estimates based on measured radii and the authors' contact model. Their dependence on confinement and interface condition is useful evidence for the importance of boundary conditions. A matched forward prediction requires the actual force, loading history and interface condition.

Table S34. Film geometry and contact-inferred compliance at 120 s for LMA with 75% by mass HDDMA, from Johnson and Stafford [11], Supporting Information, Table S1. The ± values for J are the reported standard errors. The ratios a/t follow from the reported geometry.

Interface	t, μm	a, μm	J, 10^{-9} Pa^{-1}	a/t
Fluorinated	7.3	39.7	10.6 ± 0.3	5.438
Fluorinated	11.6	46.2	9.5 ± 0.3	3.983
Fluorinated	13.9	47.5	8.0 ± 0.2	3.417
Fluorinated	15.1	48.4	7.7 ± 0.2	3.205
Fluorinated	20.1	51.2	6.3 ± 0.2	2.547
Fluorinated	26.8	52.0	4.5 ± 0.1	1.940
Fluorinated	30.5	52.7	4.1 ± 0.1	1.728
Fluorinated	33.3	54.3	4.2 ± 0.1	1.631
Methacrylate	5.2	35.1	11.2 ± 0.4	6.750
Methacrylate	6.2	36.7	9.9 ± 0.3	5.919
Methacrylate	8.9	38.7	6.5 ± 0.2	4.348
Methacrylate	11.6	41.0	5.5 ± 0.2	3.534
Methacrylate	15.1	43.2	4.5 ± 0.1	2.861
Methacrylate	20.3	46.8	4.2 ± 0.1	2.305
Methacrylate	31.2	53.3	4.2 ± 0.1	1.708

S11.5. Transfer between loading histories

Herbert et al. [12] combined oscillatory flat-punch indentation and finite-time creep measurements on plasticized polyvinyl chloride (PVC). Their four-mode creep representation is

$$D(t) = J_0 + \sum_{i=1}^{4} J_i[1 - \exp(-t/\tau_i)]. \tag{S23}$$

Table S35 reproduces the coefficients from their Figure 11, with the original mode numbering. Their Figure 12 compares the representation identified in the frequency domain with independently measured creep. The frequency-domain data span 0.01–50 Hz, and agreement with the separately measured creep is reported over approximately 0.1–10 s.

This comparison demonstrates transfer of a linear viscoelastic representation between loading histories over a stated experimental window. The coefficients characterize the tested PVC specimen. Application to the present coating requires its material spectrum and the relevant displacement reference, particularly at submillisecond times. The finite-ramp benchmark in Section S11.1 addresses the convolution operation separately from the experimental transfer of material parameters.

Table S35. Creep coefficients for plasticized PVC reported by Herbert et al. [12], Figure 11. J_0 is the instantaneous compliance; J_i, τ_i for $i = 1, \ldots, 4$ are delayed-mode amplitudes and retardation times. Mode indices follow the source.

Index	J_i, Pa^{-1}	τ_i, s
0	3.6960×10^{-8}	–
1	8.2830×10^{-8}	0.0075198
2	4.6922×10^{-8}	3.8620
3	8.8235×10^{-8}	0.041034
4	7.5425×10^{-8}	0.29883

S11.6. Load-dependent contact stiffness

Fuadi [13] determined normal contact stiffness from local slopes of force–displacement hysteresis loops at 30, 60 and 100 mN for four soft-material surfaces. Table S36 reports all 12 values. Each surface becomes stiffer as load increases, while the ranking depends on load: at 100 mN, S2 is stiffer than S1.

The loop-slope observable is a force stiffness in N/m. In contrast, the present $K_s = q_{nom}/\delta_{tot}$ and $K_t = \mathrm{d}q_{nom}/\mathrm{d}\delta_{tot}$ are secant and tangent normal stiffnesses per nominal area. Here "tangent" denotes the derivative along a normal loading curve. The class A and B elastic loading branches show increasing K_s and K_t over the evaluated states. The differences in material, geometry, loading history and area normalization make comparison with Fuadi's measurements qualitative.

Table S36. Normal contact stiffnesses reported by Fuadi [13] for four soft-material surfaces. Stiffness is the local slope of a force–displacement loop and is reported in N/m, without nominal-area normalization. All 12 load–stiffness pairs are shown.

Surface	Force, mN	Stiffness, N/m
S1	30	859
S1	60	906
S1	100	1080
S2	30	581
S2	60	890
S2	100	1200
S3	30	493
S3	60	839
S3	100	1080
S4	30	586
S4	60	815
S4	100	976

Dynamic rough-joint stiffness.

Yu et al. [14] developed a friction-dependent fractal model spanning elastic, elasto-plastic and plastic asperity deformation. Their Figure 14(a,b) compares model predictions with the theoretical Hertz and Miao references. Figure 14(c,d) reproduces previously published measurements by Jiang and co-workers on ground and milled cast-iron joints. The experimental comparison here uses Jiang and co-workers' measurements as reported by Yu et al.

The specimens have nominal contact area 10 000 mm^2, Young's modulus 100 GPa, Poisson's ratio 0.25 and Brinell hardness 220. Four symmetrically arranged bolts apply the static load. Impact excitation and accelerometer measurements provide the first two resonance frequencies, from which normal joint stiffness is identified by fitting the dynamic response. With the notation of Yu et al., the plotted quantities are $K^* = K/(E\sqrt{A_a})$ and $F^* = F/(EA_a)$, where A_a is nominal area and E is the equivalent modulus used in that normalization. In this source-specific notation, K denotes joint stiffness and G in Table S37 denotes the fractal roughness parameter; the coating bulk and shear moduli use these symbols in their own constitutive context.

The experimental markers increase with load for both machining conditions; the model curves have a similar trend and depend on the assumed friction and deformation regimes. Table S37 gives the published specimen and topography inputs. This is a metallic rough-joint comparison based on modal identification. Quantitative transfer to the present quasistatic K_t requires matching the observation operator, material response and geometry. The separate layered pressure-profile comparison in Appendix C.2 of the main text and Section S8 uses Yu et al. [6] and concerns the elastic single-asperity kernel.

Table S37. Cast-iron joint and topography inputs reported by Yu et al. [14], Section 5 and Figure 14(c,d), for experiments attributed there to Jiang and co-workers. G is the source's fractal roughness parameter, distinct from the shear modulus used in the present coating model.

Quantity	Ground interface	Milled interface
Material	Cast iron	Cast iron
Nominal area, mm^2	10 000	10 000
Young's modulus, GPa	100	100
Poisson's ratio	0.25	0.25
Brinell hardness	220	220
R_a, μm	1.02	2.47
Fractal dimension D	1.4058	1.2183
Fractal roughness G, m	2.2826×10^{-10}	5.9036×10^{-14}
Stiffness observable	Modal joint stiffness	Modal joint stiffness
Load trend in Figure 14	Increasing	Increasing

S11.7. Two-body patterned-interface comparison

Perris et al. [15] report saturated tangent normal stiffness $K = |\mathrm{d}P/\mathrm{d}(\Delta d)|$ in Table 2. Digital image correlation (DIC) uses five target pairs separated by approximately $d = 200\,\mu$m, with points about 100 μm on either side of the interface. The measured stiffness therefore includes deformation within both specimens. The as-produced ridge widths and heights are given in the source's Table 1, and its Figure 2 gives $E = 2633$ MPa and $\nu = 0.415$. The present reduction uses period $L = 500\,\mu$m, nominal area $A_n = 100\ \mathrm{mm}^2$ and rectangular ridges with $(w, h) = (80.6, 52.3)$, $(171.4, 52.8)$, $(275.4, 53.9)$ and $(387.8, 51.1)$ μm. Their width fractions are $\rho = w/L = 0.1612$, 0.3428, 0.5508 and 0.7756.

Elastic model and displacement observation.

The present finite-element reduction solves isotropic linear plane-strain elasticity for two deformable periodic bodies. The upper body is flat and 1 mm deep; the lower body extends 2 mm below the interface and carries the ridge. Uniform normal pressure is applied to the remote upper boundary, and the remote lower boundary has zero normal displacement. Periodic displacement fluctuations are combined with an independently free mean lateral strain in each body. Horizontal constraints remove rigid translations. Across the ridge top, normal displacements are continuous and tangential displacements remain independent, representing frictionless contact. Ridge walls, groove floor and the noncontacting face are traction free. Four-node isoparametric elements with full 2×2 integration resolve the ridge and both gauge levels. The linear response is evaluated at $p_0 = 1$ MPa.

The numerical observation is the spatial phase average

$$C_{g,\mathrm{FE}}(\rho) = \frac{1}{p_0 L} \int_{-L/2}^{L/2} [u_y^{\mathrm{lower}}(x, -d/2) - u_y^{\mathrm{upper}}(x, d/2)]\,\mathrm{d}x.$$

The continuous phase average approximates the displacement sampled by the five experimental target pairs, whose exact coordinates are unavailable. The range obtained by varying the sampling position is reported separately.

Calibration and comparison.

One flat-interface measurement determines the additive compliance baseline:

$$C_{g,\mathrm{pred}}(\rho) = C_{\mathrm{flat,exp}} + C_{g,\mathrm{FE}}(\rho) - C_{g,\mathrm{FE}}(1),$$
$$C_{\mathrm{flat,exp}} = \frac{A_n}{K_{\mathrm{flat}}}, \qquad K_{\mathrm{pred}} = \frac{A_n}{C_{g,\mathrm{pred}}}.$$

The central value $K_{\text{flat}} = 226.2$ kN/mm gives $C_{\text{flat,exp}} = 0.442086649\,\mu$m/MPa. The periodic flat solution gives $C_{g,\text{FE}}(1) = d(1-\nu^2)/E = 0.062876946\,\mu$m/MPa, leaving a common additive background of $0.379209703\,\mu$m/MPa. The reported flat measurement is 226.2 ± 10.3 kN/mm. The material properties and ridge dimensions are fixed by the source's Figure 2 and Table 1, respectively, and the flat measurement determines the common baseline. The prediction-error statistics use the four patterned specimens only.

For each patterned specimen, $e_i = 100(K_{i,\text{pred}} - K_{i,\text{exp}})/K_{i,\text{exp}}$. The mean absolute relative error is $n^{-1}\sum_i |e_i|$, and the maximum is $\max_i |e_i|$, with $n = 4$. All four specimens give a mean of 20.05% and a maximum of 58.88%. Table S38 reports experimental uncertainties alongside stiffness predictions to 0.1 kN/mm. The predicted stiffnesses are 124.0876, 164.0583, 195.2470 and 217.3080 kN/mm, with signed errors of +58.8829%, +15.9422%, −1.3406% and +4.0249%, respectively. The corresponding phase limits are 109.58–158.13, 141.84–199.90, 171.04–217.82 and 202.42–224.66 kN/mm.

Stiffness in kN/mm is numerically equal to N/μm; dividing by $A_n = 100$ mm^2 gives nominal pressure stiffness in MPa/μm. The errors use central experimental values. The phase ranges quantify deterministic position-dependent variation; experimental uncertainty is reported separately.

Narrow-ridge discrepancy and numerical sensitivity.

For the 80.6 μm ridge, the predicted stiffness is 124.1 kN/mm and the minimum over sampling position is 109.58 kN/mm, compared with the experimental 78.1 ± 4.0 kN/mm. A prescribed taper retaining the measured top width and height gives 133.14 kN/mm. Its base width is prescribed from the design geometry. These sensitivities identify influential geometric and observation assumptions; the cause of the narrow-ridge discrepancy remains unresolved. The error statistics include all four specimens. A physical applicability threshold for the narrow ridge remains undetermined for this finite-height elastic problem.

Four meshes use 16, 32, 64 and 128 elements across the ridge top. Final refinement changes the calibrated stiffness by at most 0.058%, whereas the relative change in the small ridge compliance increment reaches approximately 1.02%. Doubling the remote depths changes the stiffnesses for the two extreme widths by less than 0.005%. For the four final rectangular cases, relative force and energy residuals are below 1.4×10^{-11} and all contact reactions are compressive. The flat solution agrees with $d(1-\nu^2)/E$ to approximately 4×10^{-14} relative difference. These results establish numerical consistency of the specified reduction, separately from its experimental discrepancies. The comparison concerns saturated, uncoated, two-body elastic normal stiffness. A coated rough interface with hereditary deformation requires its corresponding geometry, displacement observation and loading history.

Table S38. Saturated normal stiffnesses measured by Perris et al. [15], Table 2, and the present two-body periodic elastic predictions. The flat datum $K_{flat} = 226.2 \pm 10.3$ kN/mm calibrates the common compliance baseline; the error statistics use the four patterned specimens. Experimental ± values are reported as given in the source. The phase range is a deterministic position-dependent range. Stiffnesses are in kN/mm and dimensions in μm; errors use experimental central values as denominator.

w	h	ρ	K_{exp}	K_{pred}	e_i, %	Phase range
80.6	52.3	0.1612	78.1 ± 4.0	124.1	+58.88	109.6–158.1
171.4	52.8	0.3428	141.5 ± 5.9	164.1	+15.94	141.8–199.9
275.4	53.9	0.5508	197.9 ± 5.1	195.2	-1.34	171.0–217.8
387.8	51.1	0.7756	208.9 ± 2.2	217.3	+4.02	202.4–224.7

References

1. Greenwood, J.A.; Williamson, J.B.P. Contact of nominally flat surfaces. *Proc. R. Soc. Lond. A* **1966**, *295*, 300–319. https://doi.org/10.1098/rspa.1966.0242.
2. Morris, M.D. Factorial sampling plans for preliminary computational experiments. *Technometrics* **1991**, *33*, 161–174. https://doi.org/10.1080/00401706.1991.10484804.
3. Almqvist, A.; Campaña, C.; Prodanov, N.; Persson, B.N.J. Interfacial separation between elastic solids with randomly rough surfaces: Comparison between theory and numerical techniques. *J. Mech. Phys. Solids* **2011**, *59*, 2355–2369. https://doi.org/10.1016/j.jmps.2011.08.004.
4. McCool, J.I. Extending the capability of the Greenwood Williamson microcontact model. *J. Tribol.* **2000**, *122*, 496–502.
5. Ciavarella, M.; Greenwood, J.A.; Paggi, M. Inclusion of interaction in the Greenwood and Williamson contact theory. *Wear* **2008**, *265*, 729–734. https://doi.org/10.1016/j.wear.2008.01.019.
6. Yu, C.; Wang, Z.; Wang, Q.J. Analytical frequency response functions for contact of multilayered materials. *Mech. Mater.* **2014**, *76*, 102–120. https://doi.org/10.1016/j.mechmat.2014.06.006.
7. Putignano, C.; Carbone, G.; Dini, D. Mechanics of rough contacts in elastic and viscoelastic thin layers. *Int. J. Solids Struct.* **2015**, *69–70*, 507–517. https://doi.org/10.1016/j.ijsolstr.2015.04.034.
8. Oyen, M.L. Spherical Indentation Creep Following Ramp Loading. *J. Mater. Res.* **2005**, *20*(8), 2094–2100. https://doi.org/10.1557/jmr.2005.0259.
9. Gacoin, E.; Fretigny, C.; Chateauminois, A.; Perriot, A.; Barthel, E. Measurement of the mechanical properties of thin films mechanically confined within contacts. *Tribol. Lett.* **2006**, *21*(3), 245–252. https://doi.org/10.1007/s11249-006-9030-y.
10. Cheneler, D.; Mehrban, N.; Bowen, J. Spherical indentation analysis of stress relaxation for thin film viscoelastic materials. *Rheol. Acta* **2013**, *52*(7), 695–706. https://doi.org/10.1007/s00397-013-0707-5.
11. Johnson, P.M.; Stafford, C.M. Effect of Interfacial Adhesion on Viscoelastic Relaxation Processes in Thin Polymer Film Indentation. *ACS Appl. Mater. Interfaces* **2010**, *2*(7), 2108–2115. https://doi.org/10.1021/am100369z.
12. Herbert, E.G.; Oliver, W.C.; Lumsdaine, A.; Pharr, G.M. Measuring the constitutive behavior of viscoelastic solids in the time and frequency domain using flat punch nanoindentation. *J. Mater. Res.* **2009**, *24*(3), 626–637. https://doi.org/10.1557/jmr.2009.0089.
13. Fuadi, Z. An Experimental Study on the Effect of Surface Texture on Contact Stiffness Using Soft Material. *MATEC Web Conf.* **2018**, *217*, 02005. https://doi.org/10.1051/matecconf/201821702005.
14. Yu, X.; Sun, Y.; Zhao, D.; Wu, S. A revised contact stiffness model of rough curved surfaces based on the length scale. *Tribol. Int.* **2021**, *164*, 107206. https://doi.org/10.1016/j.triboint.2021.107206.
15. Perris, J.; Xu, Y.; Kartal, M.E.; Gadegaard, N.; Mulvihill, D.M. Tailorable and Repeatable Normal Contact Stiffness via Micropatterned Interfaces. *Tribol. Lett.* **2021**, *69*(3), 106. https://doi.org/10.1007/s11249-021-01473-3.

Disclaimer/Publisher's Note: The statements, opinions and data contained in all publications are solely those of the individual author(s) and contributor(s) and not of MDPI and/or the editor(s). MDPI and/or the editor(s) disclaim responsibility for any injury to people or property resulting from any ideas, methods, instructions or products referred to in the content.
